# Supplementary material for: Identification of AGR2 Gene-Specific Expression Patterns Associated with Epithelial-Mesenchymal Transition
Source: Int J Mol Sci. 2022 Sep 16;23(18):10845. doi: 10.3390/ijms231810845 (PMC9504245; doi:10.3390/ijms231810845)
Supplement: Supplementary file 1 [file ijms-23-10845-s001.zip › SI FIGURES together_220830.pdf]

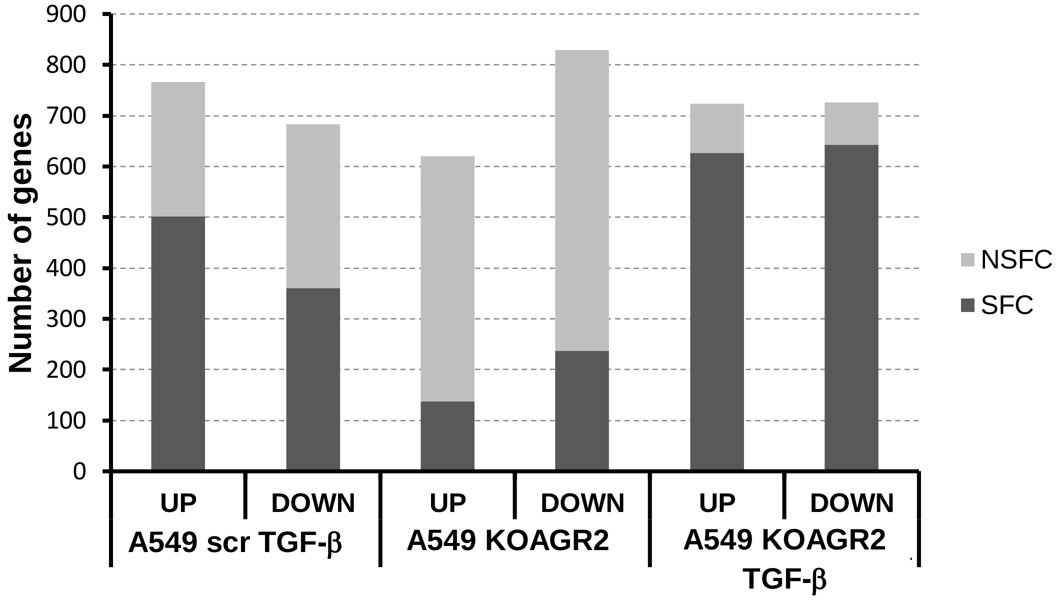

Figure S1: Transcriptome analysis. Number of genes with adjusted P-level ≤ 0.05 in both replicates showing similar expression profiles that were identified by RNAseq. NSFC = genes with nonsignificant expression FC, SFC = genes with significant expression FC (outside the range 0.67>FC>1.5) in relation to untreated A549 scr cells.

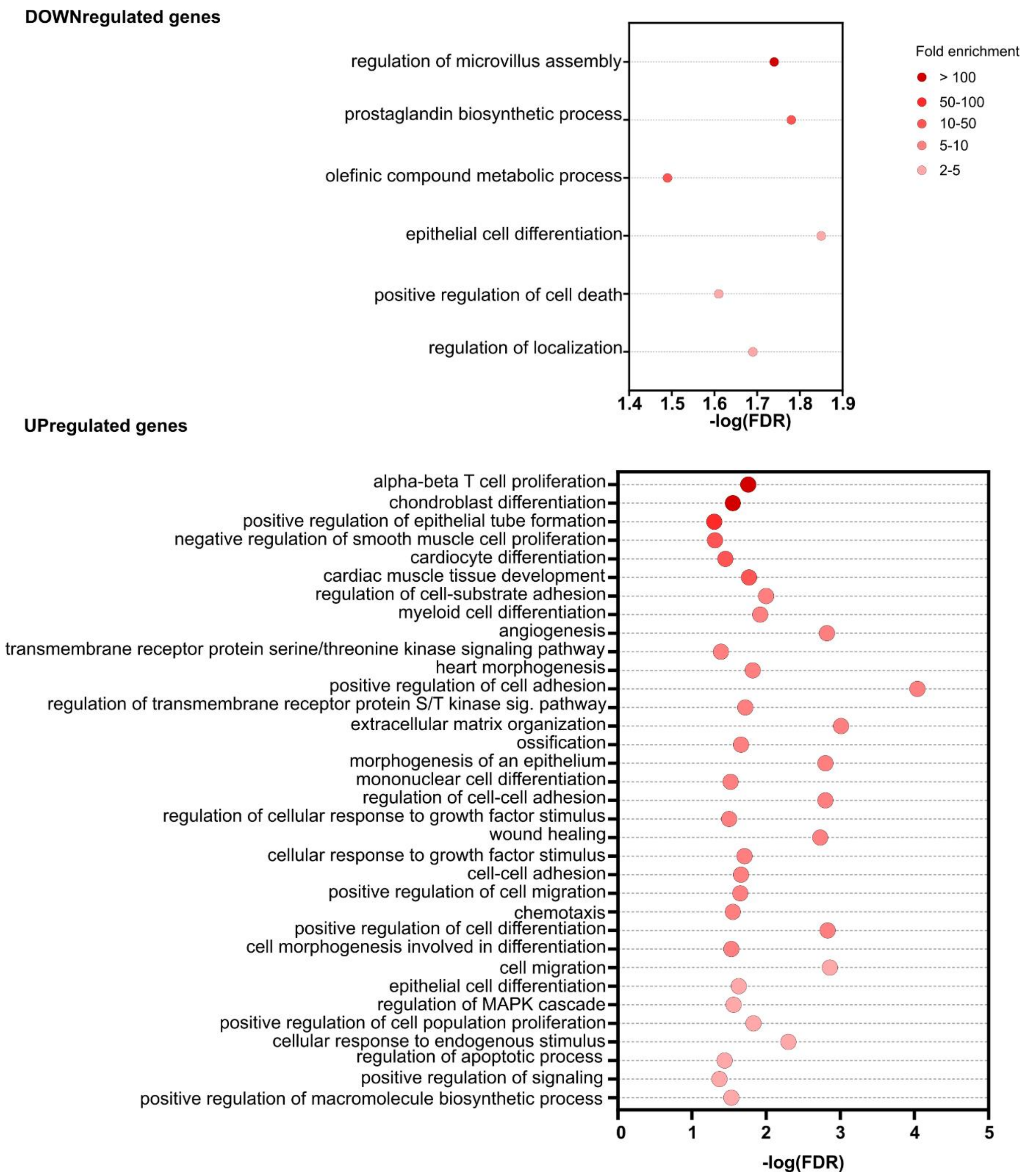

Figure S2: Functional enrichment analysis of differentially expressed genes based on GO biological process terms. The colour of circle represents the fold enrichment as indicated. Related to the Figure1

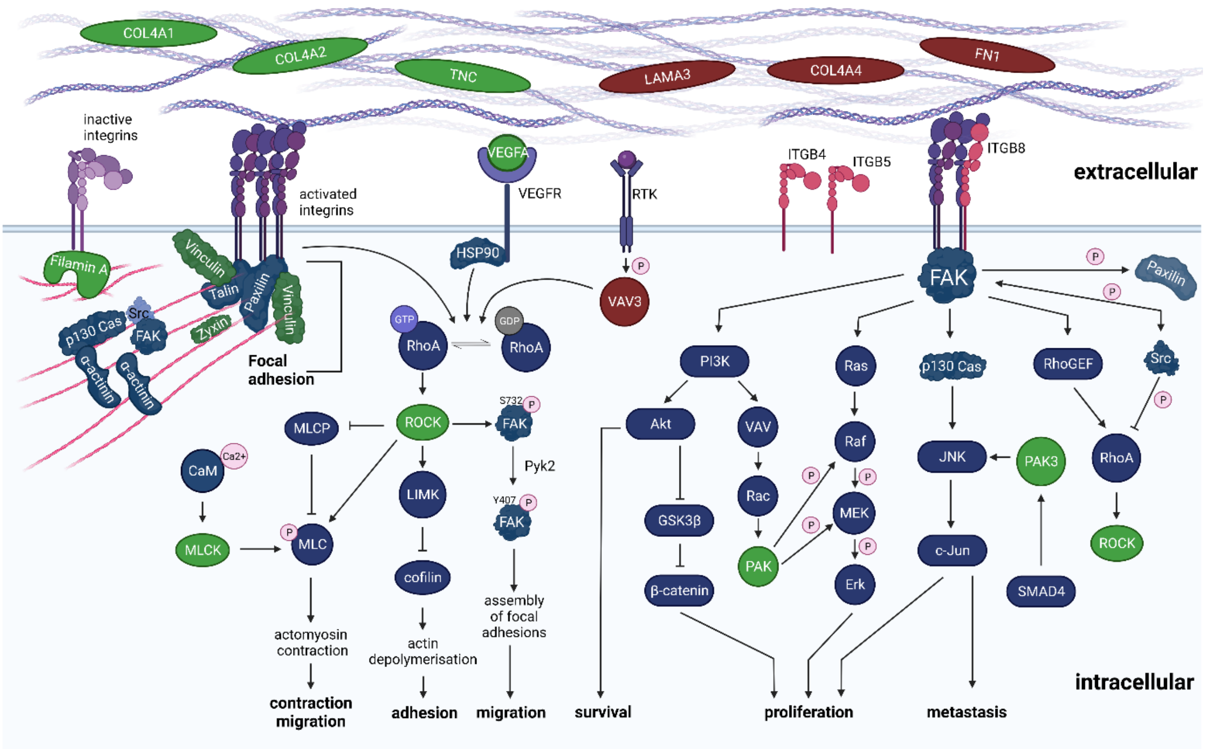

Figure S3: Focal adhesion. The green colour represents upregulated, and red downregulated proteins based on our transcriptomic profile in A549 with *AGR2* gene knockout. Focal adhesion is depicted as containing only the most important proteins as it is a huge multiprotein complex structure containing more than thirty adaptor proteins that provide a binding for hundreds of other intracellular FA proteins. Filamin A acts as an actin crosslinking protein. Vinculin and Zyxin are parts of the Focal adhesion foci. Rock is a kinase with wide downstream effects. It inhibits MLCP, which increases the levels of p-MLC, meanwhile it also directly phosphorylates MLC. MLC can also be phosphorylated by MLCK (MYLK). Phosphorylated MLC leads to contractile movement. Rock also phosphorylates LIMK, which leads to adhesion, and FAK, which results in FA formation. Upstream of Rock, there are RhoA whose activity is regulated by VAV3 (belongs to RhoGEF), which RTKs activate. HSP90 also regulates RhoA through activated VEGFR (with a bound ligand such as VEGFA) or by signals from activated integrins. The most prominent effector of activated integrins is FAK which can phosphorylate Src and vice versa. Src can inhibit RhoA, or FAK can activate RhoGEF (which also includes the VAV family), and RhoGEFs then activate RhoA pathways. Furthermore, FAK can activate the JNK/cJun pathway, leading to proliferation and metastasis. JNK is also activated by PAK3, a downstream effector of SMAD4. FAK also activates the RAS pathway, leading to proliferation and PI3K/Akt pathways leading to proliferation and survival. PI3K can also activate VAVs, which are already mentioned RhoGEF (mainly VAV1 and VAV2). They further activate PAK(1-6), which are connected with the Ras pathway through phosphorylation of Raf and MEK. We also identified changes in transcripts corresponding to several ITGBs – out of them, ITGB8 was described to interact with FAK. Our RNAseq data also described transcripts corresponding to ECM proteins such as collagens, Tenascin C (glycoprotein), LAMA3 (part of laminins), and Fibronectin 1, which all were described to bind to various classes of integrins. FA, focal adhesion; MLCP, Myosin Light Chain Phosphatase; p-MLC, phosphorylated Myosin Light Chain; MLCK/MYLK, Myosin Light Chain Kinase; LIMK, LIM domain kinase; FAK, Focal Adhesion Kinase; RhoA, Ras homolog family member A; RhoGEF, Rho guanine nucleotide exchange factor; RTKs, receptor tyrosine kinases; VEGFR, Vascular endothelial growth factor receptor;

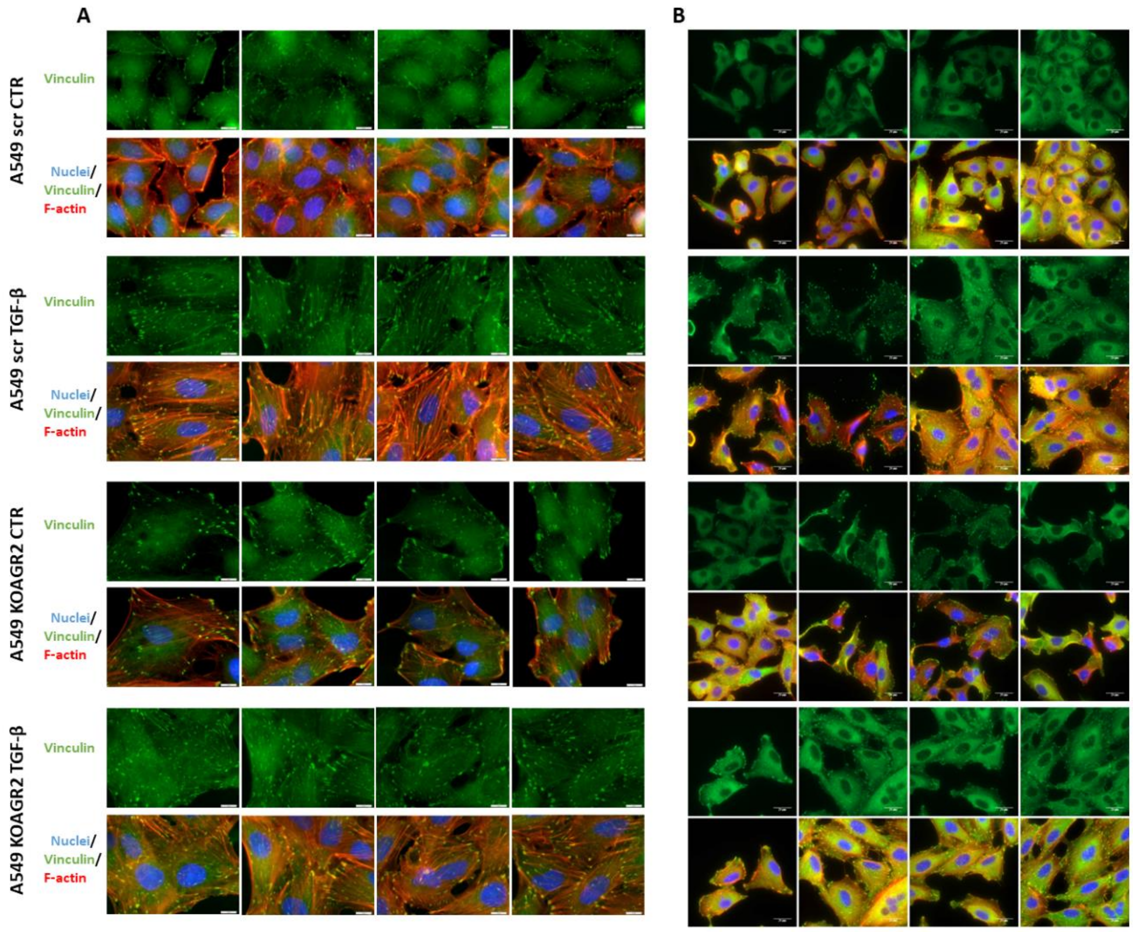

Figure S4: Additional IFC slides. F-actin stress fibres (red), vinculin foci (green) and nuclei (blue) by Hoechst. Additional slides were captured at (**A**) 100× magnification, scale bar represents 10 μm and (**B**) 60× magnification, scale bar represents 20 μm. Scrambled control cells show smaller and less frequent foci in contrast to AGR2 knockout or TGF-β treatment..

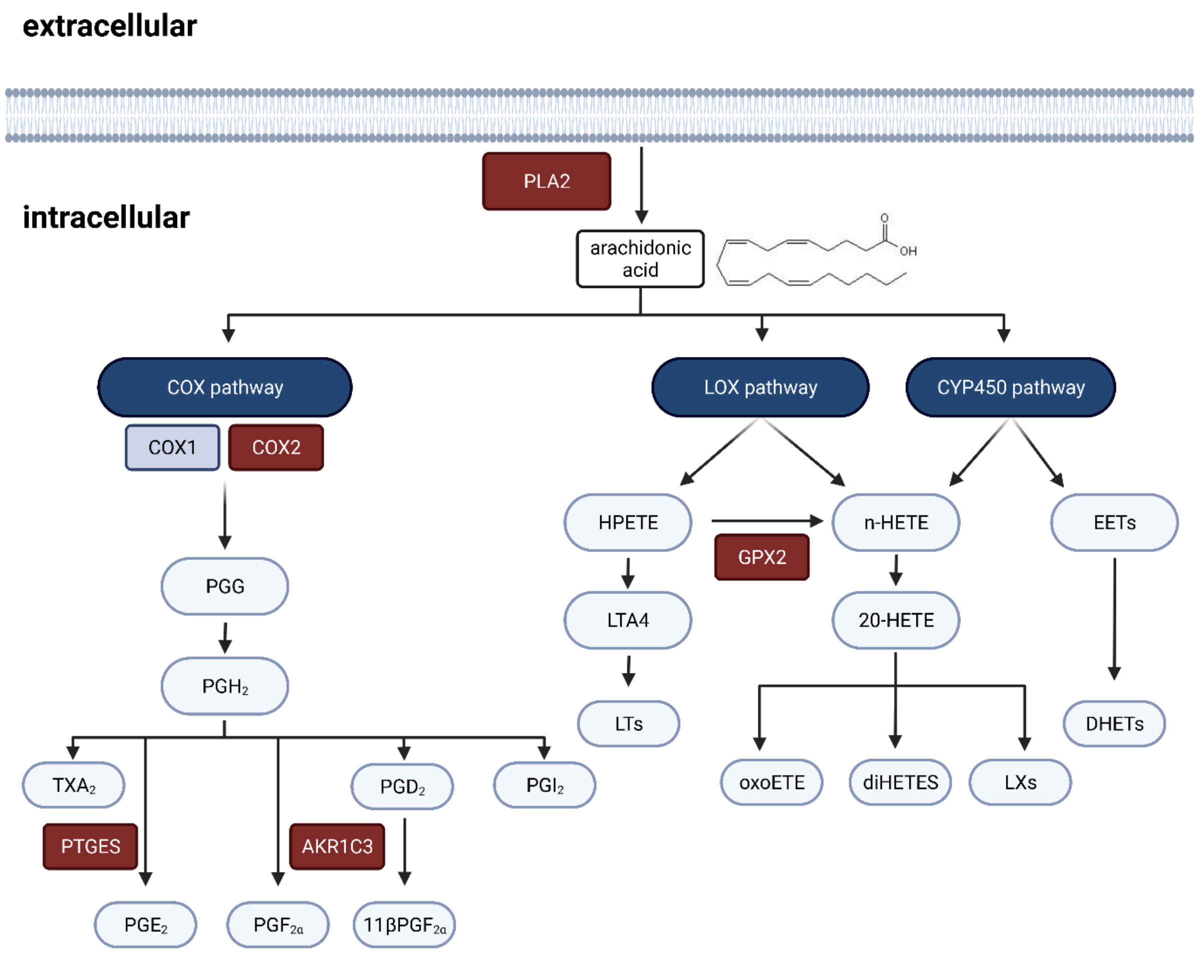

Figure S5: Arachidonic acid metabolism. The red colour represents downregulated proteins based on our transcriptomic profile in A549 where the transcripts were significant for both AGR2KO and scr cells after TGF-β. Arachidonic acid is hydrolysed and released from cell membrane phospholipids by phospholipases (PLA2 family, such as PLA2G4A). Afterward, it is further metabolised through different downstream pathways – one of which is the COX pathway (including enzyme COX2 or PTGS2) leading to the production of PGH_2 which is further metabolised to thromboxanes, prostacyclin, or prostaglandins (PGE_2 by PTGES, $PGF_{2\alpha}$ by AKR1C3 which also metabolises PGD_2 further to $11\beta PGF_{2\alpha}$). LOX pathways represent another possibility of arachidonic acid metabolism, and its metabolite HPETE can be further processed to n-HETE by GPX2, with n-HETE also being part of the CYP450 pathway. PLA2, phospholipase A2; COX, cyclooxygenase; PGG, prostaglandin G; PGH_2, prostaglandin H2; TXA_2, thromboxane A2; PGE_2, prostaglandin E2; $PGF_{2\alpha}$, prostaglandin F2α; PGD_2, prostaglandin G2; $11\beta PGF_{2\alpha}$, 11β-prostaglandin F2 α; PGI_2, prostacyclin I2; LOX, lipooxygenase; HPETE, hydroperoxyeicosatetraenoic acid; HETE, Hydroxyeicosatetraenoic acid; LTA4, leukotriene A4; LTs, leukotrienes; oxoETE, oxo-eicosatetraenoic acid; LXs, lipoxins; diHETES, dihydroxyeicosatetraenoic acid; CYP450, cytochrome P450; EETs, epoxyeicosatrienoic acids; DHETs, dihydroxyeicosatrienoic acids.

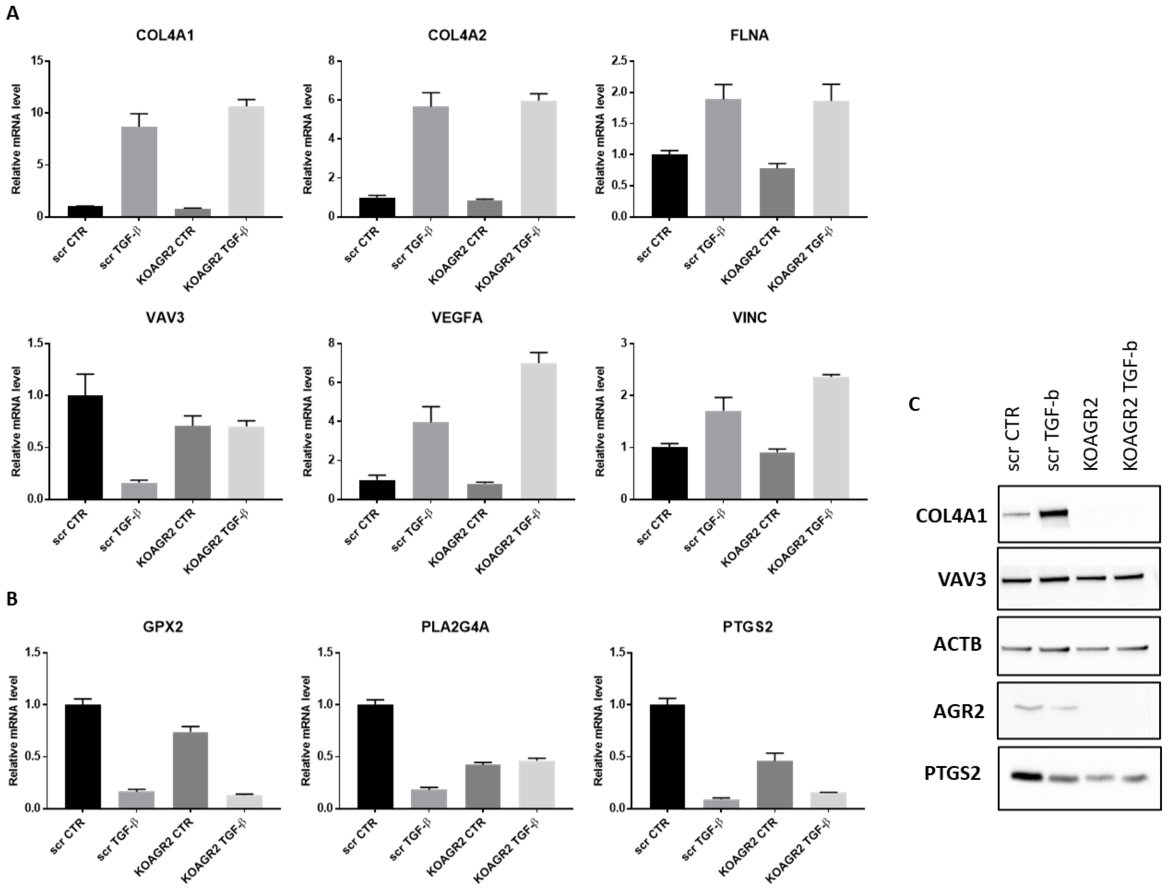

Figure S6: Validation of mRNA levels of representative genes by RT-qPCR for (A) Focal adhesion pathway and (B) arachidonic acid metabolism pathway. Graphs show gene expression normalized to GAPDH that was used as an endogenous control. In parallel endogenous control, 18S rRNA, was used with similar outputs (data not shown). (C) Representative immunochemical analysis of COL4A1, VAV3, PTGS2 and AGR2. Beta-actin served as a loading control.

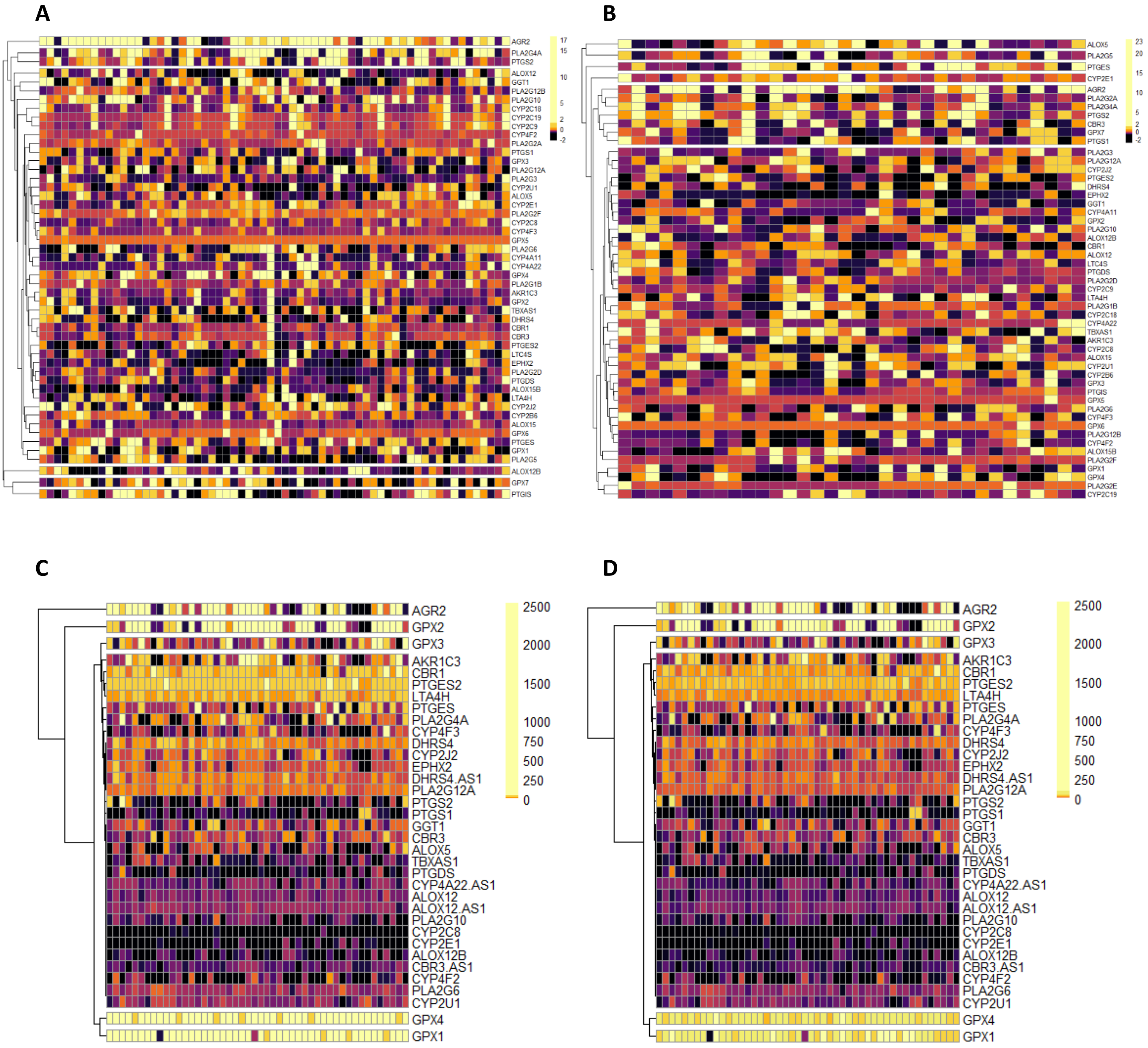

Figure S7. Correlation between *AGR2* and genes of prostaglandin biosynthesis. Heat maps represent the involvement of AGR2 in arachidonic acid metabolism in (A) lung tumours and (B) large intestine tumours expression datasets using COSMIC and (C) lung cell lines and (D) colorectal cell lines using the Cancer Cell Line Encyclopedia. Values are expressed as z-score for Cancer Cell Line Encyclopedia and FKPM for COSMIC.
