# Supplementary material for: Identification of AGR2 Gene-Specific Expression Patterns Associated with Epithelial-Mesenchymal Transition
Source: Int J Mol Sci. 2022 Sep 16;23(18):10845. doi: 10.3390/ijms231810845 (PMC9504245; doi:10.3390/ijms231810845)
Supplement: Supplementary file 1 [file ijms-23-10845-s001.zip › Table S1.pdf]

Table S1: Transcripts with their corresponding log fold change with adjusted P-value < 0.05

	scr vs TGFbeta		scr vs KO		scr vs KO+TGFbeta		TGFbeta vs KO+TGFbeta			TGFbeta vs KO			KO+TGFbeta vs KO		
**Row.names**	**X[1]**	**Log2FC**	**X**	**Log2FC**	**X**	**Log2FC**	**X**	**sign[2]**	**Log2FC**	**X**	**sign**	**Log2FC**	**X**	**sign**	**Log2FC**
RP11-395B7.7	46	0,9775	152	-0,3194	303	-0,7095	2	-1	-1,6870	21	-1	-1,2969	802	1	0,3901
PXDN	20051	0,0100	5	-1,0451	22	-1,6078	3	-1	-1,6178	41	-1	-1,0552	412	1	0,5627
HPGD	2932	0,1553	194	-0,2914	49	-1,2734	4	-1	-1,4287	557	-1	-0,4467	77	1	0,9820
HIST1H4L	3623	0,1348	14	-0,6787	44	-1,2916	5	-1	-1,4264	117	-1	-0,8136	330	1	0,6128
TP63	409	0,4766	113	-0,3635	238	-0,7787	8	-1	-1,2553	104	-1	-0,8401	730	1	0,4152
TNC	10907	-0,0400	10	0,7451	84	1,0763	12	-1	1,1163	134	-1	0,7851	1075	1	-0,3311
HNF4A	77	0,8211	2116	-0,1028	1972	-0,2607	13	-1	-1,0818	74	-1	-0,9238	3089	1	0,1579
ABCA1	1121	0,2811	246	-0,2620	229	-0,7865	14	-1	-1,0676	362	-1	-0,5431	471	1	0,5245
PAK3	11866	-0,0348	74	0,4093	160	0,9068	23	-1	0,9416	566	-1	0,4442	521	1	-0,4974
GREM1	134	0,7221	63	-0,4418	3053	-0,1927	26	-1	-0,9148	27	-1	-1,1639	1648	1	-0,2491
F11R	1246	0,2640	47	-0,4721	407	-0,6260	29	-1	-0,8900	165	-1	-0,7360	3199	1	0,1539
UACA	74	0,8310	223	-0,2727	14062	-0,0282	34	-1	-0,8592	30	-1	-1,1038	1694	1	-0,2446
DMBT1	25405	-0,0051	54	0,4622	204	0,8274	38	-1	0,8325	510	-1	0,4673	916	1	-0,3652
SEMA3E	360	0,5115	112	-0,3645	1497	-0,3111	39	-1	-0,8226	89	-1	-0,8760	9178	1	-0,0534
F2RL1	1505	0,2371	22	-0,5752	494	-0,5748	41	-1	-0,8119	119	-1	-0,8123	29102	1	-0,0004
TM4SF1	4695	0,1102	30	-0,5182	337	-0,6768	47	-1	-0,7870	243	-1	-0,6284	3078	1	0,1586
ITGB4	254	0,5830	229	-0,2701	2997	-0,1954	51	-1	-0,7785	99	-1	-0,8532	7152	1	-0,0747
FST	183	0,6642	238	-0,2655	5762	-0,1125	53	-1	-0,7767	68	-1	-0,9297	3231	1	-0,1530
SLC16A4	919	0,3161	296	-0,2451	801	-0,4546	55	-1	-0,7707	335	-1	-0,5612	2095	1	0,2094
BCL2A1	25402	-0,0051	13302	0,0106	251	0,7655	56	-1	0,7706	16985	-1	0,0157	198	1	-0,7549
STXBP6	8270	0,0615	145	-0,3270	326	-0,6878	61	-1	-0,7493	738	-1	-0,3884	941	1	0,3609
ACSF2	1677	0,2199	95	-0,3791	636	-0,5133	64	-1	-0,7332	278	-1	-0,5990	3836	1	0,1342
GLUL	2013	0,1947	108	-0,3710	587	-0,5349	65	-1	-0,7296	325	-1	-0,5656	2954	1	0,1639
ADD3	15814	0,0196	142	-0,3279	308	-0,7074	66	-1	-0,7270	898	-1	-0,3475	850	1	0,3794
COL4A4	404	0,4804	359	-0,2268	2391	-0,2289	72	-1	-0,7094	190	-1	-0,7072	28333	1	0,0022
PTPRD	19330	-0,0114	65	0,4348	316	0,6979	73	-1	0,7093	559	-1	0,4461	1532	1	-0,2631
CELSR3	14095	0,0250	50	-0,4661	344	-0,6716	76	-1	-0,6966	456	-1	-0,4911	2153	1	0,2055
SCD	762	0,3502	31	-0,5104	1292	-0,3412	77	-1	-0,6914	95	-1	-0,8606	2841	1	-0,1692
GBP1	6333	-0,0842	168	0,3084	434	0,6062	78	-1	0,6904	723	-1	0,3925	1273	1	-0,2979

CAPG	4027	0,1245	283	-0,2484	565	-0,5444	86	-1	-0,6689	786	-1	-0,3729	1282	1	0,2960
ARAP2	7516	0,0697	264	-0,2559	444	-0,5985	87	-1	-0,6682	1014	-1	-0,3256	1024	1	0,3426
PLEKHA6	6806	0,0777	100	-0,3758	472	-0,5889	88	-1	-0,6666	541	-1	-0,4536	2052	1	0,2130
HOXB9	19135	-0,0123	488	0,2027	368	0,6513	91	-1	0,6635	2130	-1	0,2149	637	1	-0,4486
SORT1	5214	0,1012	131	-0,3385	522	-0,5585	92	-1	-0,6597	581	-1	-0,4397	1976	1	0,2200
ATP9A	6788	0,0779	105	-0,3718	514	-0,5632	103	-1	-0,6411	549	-1	-0,4497	2387	1	0,1914
FLRT3	1173	0,2736	252	-0,2601	1234	-0,3509	114	-1	-0,6245	376	-1	-0,5337	5998	1	0,0907
CCL2	1104	-0,2837	302	0,2443	1325	0,3350	116	-1	0,6187	384	-1	0,5280	5999	1	-0,0907
CTD-2377D24.8	18519	-0,0131	9803	0,0210	441	0,5995	120	-1	0,6126	11907,5	-1	0,0341	381	1	-0,5785
MAP7	15174	0,0208	35	-0,4931	474	-0,5871	125	-1	-0,6080	407	-1	-0,5139	5797	1	0,0940
THY1	8210	0,0622	226	-0,2723	574	-0,5414	126	-1	-0,6036	966	-1	-0,3345	1473	1	0,2691
RPL23A	6427	0,0827	796	-0,1645	734	-0,4795	151	-1	-0,5622	1676	-1	-0,2472	1156	1	0,3150
DCLK1	2384	-0,1767	26	0,5528	1075	0,3829	153	-1	0,5596	170	-1	0,7295	2827	1	0,1699
IL8	9326	-0,0516	45	0,4745	678	0,4958	162	-1	0,5474	388	-1	0,5262	14984	1	-0,0213
RGS3	12107	-0,0337	3889	0,0679	640	0,5117	164	-1	0,5454	5654	-1	0,1015	652	1	-0,4438
GPRC5B	2068	0,1921	5074	-0,0534	1465	-0,3149	200	-1	-0,5070	1702	-1	-0,2455	1543	1	0,2615
APOBEC3C	6322	0,0843	1317	-0,1316	987	-0,4083	212	-1	-0,4927	2107	-1	-0,2159	1406	1	0,2767
ITGB5	8761	0,0566	99	-0,3761	909	-0,4240	230	-1	-0,4806	601	-1	-0,4327	9861	1	0,0479
TUSC3	14880	-0,0221	70	0,4138	793	0,4565	235	-1	0,4786	593	-1	0,4359	10568	1	-0,0427
CTD-2354A18.1	817	-0,3406	197	0,2855	4868	0,1325	241	-1	0,4731	247	-1	0,6262	3230	1	0,1531
SLC8A1	6198	-0,0862	1280	0,1336	1101	0,3777	251	-1	0,4640	2045	-1	0,2198	1699	1	-0,2442
NDRG4	2304	0,1802	509	-0,1989	1832	-0,2738	266	-1	-0,4540	763	-1	-0,3791	7125	1	0,0749
TSPAN15	1228	0,2663	642	-0,1808	3441	-0,1774	281	-1	-0,4437	555	-1	-0,4471	24928	1	-0,0034
ANK3	3358	0,1425	1884	-0,1096	1666	-0,2918	297	-1	-0,4343	1628	-1	-0,2521	2560,5	1	0,1822
FLCN	4596	0,1120	370	-0,2237	1427	-0,3213	300	-1	-0,4332	958	-1	-0,3357	5567	1	0,0975
BNIP3L	900	0,3200	326	-0,2375	5819	-0,1115	303	-1	-0,4315	342	-1	-0,5575	4136	1	-0,1260
HNRNPA1P33	5766	-0,0924	269	0,2537	1400	0,3255	331	-1	0,4179	902	-1	0,3462	7369	1	-0,0718
STC2	1756	-0,2138	15	0,6693	3014	0,1950	351	-1	0,4088	84	-1	0,8831	566	1	0,4743
C15orf52	1832	-0,2081	2483	0,0934	2958	0,1971	359	-1	0,4053	1161	-1	0,3015	5229	1	-0,1037
KIRREL	5536	-0,0959	153	0,3194	1516	0,3085	362	-1	0,4044	645	-1	0,4153	19935	1	0,0109
MPP2	6483	0,0821	1491	-0,1243	1431	-0,3209	365	-1	-0,4030	2277	-1	-0,2065	2284	1	0,1965
STX8	3489	0,1384	659	-0,1785	1981	-0,2599	371	-1	-0,3983	1061	-1	-0,3169	6611	1	0,0814
SUPT4H1	10140	0,0454	794	-0,1647	1238	-0,3506	372	-1	-0,3960	2226	-1	-0,2101	2488	1	0,1859

ELOVL4	5377	0,0986	1908	-0,1091	1612	-0,2971	374	-1	-0,3957	2263	-1	-0,2076	2447	1	0,1880
TMEM154	4585	0,1123	1485	-0,1246	1795	-0,2784	387	-1	-0,3907	1804	-1	-0,2369	3202	1	0,1538
AC005682.5	26910	-0,0022	7159	0,0353	1063	0,3850	398	-1	0,3872	11318	-1	0,0375	985	1	-0,3497
OGFRL1	7162	-0,0734	373	0,2234	1531	0,3063	422	-1	0,3797	1196	-1	0,2968	6495	1	-0,0829
RSPO3	929	-0,3134	13	0,6800	9044	0,0644	427	-1	0,3778	54	-1	0,9934	327	1	0,6156
LGALS3	19455	0,0111	397	-0,2186	1210	-0,3564	463	-1	-0,3675	1901	-1	-0,2297	3717	1	0,1378
TRIP12	25778	-0,0042	270	0,2537	1221	0,3541	491	-1	0,3582	1565	-1	0,2579	5410	1	-0,1003
GCA	24629	0,0063	1103	-0,1431	1241	-0,3496	501	-1	-0,3559	3636	-1	-0,1494	2133	1	0,2065
BDKRB2	13081	-0,0289	627	0,1824	1395	0,3261	505	-1	0,3550	2204	-1	0,2113	3507	1	-0,1437
SAT1	9216	0,0526	27	-0,5391	1584	-0,3005	512	-1	-0,3532	287	-1	-0,5917	1760	1	-0,2385
AFAP1L2	7839	0,0661	1131	-0,1417	1815	-0,2754	552	-1	-0,3414	2261	-1	-0,2078	3861	1	0,1337
RAP1GAP2	1857	0,2061	492	-0,2016	5090	-0,1271	584	-1	-0,3332	667	-1	-0,4077	7166	1	-0,0745
SEC14L4	3136	0,1489	4881	-0,0556	3355	-0,1806	600	-1	-0,3295	2324	-1	-0,2045	4181	1	0,1250
SPOCD1	6563	-0,0812	1407	0,1278	2148	0,2464	608	-1	0,3276	2246	-1	0,2090	4471	1	-0,1186
TRABD2A	1441	0,2442	351	-0,2289	7660	-0,0820	611	-1	-0,3261	490	-1	-0,4731	3406	1	-0,1469
PARP8	5157	0,1019	3328	-0,0764	2611	-0,2153	668	-1	-0,3172	2837	-1	-0,1783	3676	1	0,1389
PCTP	8403	0,0602	593	-0,1862	2018	-0,2563	672	-1	-0,3165	1688	-1	-0,2464	7504	1	0,0701
CALCOCO2	5271	0,1002	662	-0,1781	2597	-0,2160	676	-1	-0,3162	1364	-1	-0,2783	11284	1	0,0379
HID1	14094	0,0250	377	-0,2219	1714	-0,2869	694	-1	-0,3119	1680	-1	-0,2469	7946	1	0,0651
MYH14	3896	0,1277	1035	-0,1464	3324	-0,1822	707	-1	-0,3098	1403	-1	-0,2741	11605	1	0,0357
NFIB	3231	-0,1453	288	0,2467	3957	0,1590	738	-1	0,3044	725	-1	0,3920	6184	1	0,0876
CCND3	11198	0,0382	3285	-0,0771	1924	-0,2649	743	-1	-0,3031	4968	-1	-0,1153	2452	1	0,1878
CLU	11793	0,0355	997	-0,1490	1977	-0,2602	793	-1	-0,2957	2697	-1	-0,1846	4840	1	0,1111
NARF	5170	0,1017	436	-0,2109	3038	-0,1936	794	-1	-0,2954	1093	-1	-0,3127	16457	1	-0,0173
DHX40	27953	0,0014	176	-0,3038	1677	-0,2911	817	-1	-0,2925	1140	-1	-0,3052	18258	1	-0,0127
TRHDE-AS1	24452	-0,0066	5295	0,0513	1766	0,2816	850	-1	0,2882	8854	-1	0,0579	1860	1	-0,2303
GLOD4	26944	0,0019	434	-0,2111	1729	-0,2855	859	-1	-0,2874	2175	-1	-0,2130	7172	1	0,0744
FDFT1	13005	0,0293	150	-0,3209	2024	-0,2556	876	-1	-0,2849	883	-1	-0,3502	7933	1	-0,0653
BICD1	17094	-0,0158	2393	0,0953	1900	0,2671	885	-1	0,2830	5159	-1	0,1112	2780	1	-0,1718
CNTROB	20241	0,0095	2645	-0,0893	1835	-0,2734	886	-1	-0,2829	5791	-1	-0,0989	2530	1	0,1841
BECN1	4328	0,1179	581	-0,1880	3780	-0,1648	889	-1	-0,2827	1137	-1	-0,3058	14453	1	-0,0231
WNT5A	5213	0,1012	3643	-0,0717	3459	-0,1769	929	-1	-0,2781	2970	-1	-0,1729	5144	1	0,1052
BAIAP2-AS1	26584	0,0031	16097	-0,0070	1821	-0,2746	934	-1	-0,2777	19649	-1	-0,0100	1491	1	0,2677

MOCS1	9657	0,0489	2091	-0,1033	2400	-0,2281	937	-1	-0,2771	3546	-1	-0,1523	4192	1	0,1248
ZBTB18	1953	-0,1994	107	0,3711	8026	0,0769	943	-1	0,2763	319	-1	0,5706	1292	1	0,2943
ABCG1	11990	0,0345	3211	-0,0786	2223	-0,2416	944	-1	-0,2761	5086	-1	-0,1131	2973	1	0,1630
MAP3K8	26679	0,0024	367	-0,2241	1875	-0,2696	980	-1	-0,2720	1952	-1	-0,2266	10167	1	0,0454
ANKRD29	10304	0,0444	12964	-0,0114	2466	-0,2244	1007	-1	-0,2687	9049	-1	-0,0558	2054	1	0,2130
PDK2	15235	0,0205	1548	-0,1214	2206	-0,2426	1063	-1	-0,2630	3900	-1	-0,1418	4353	1	0,1212
ADAMTS16	9304	-0,0518	735	0,1704	2776	0,2070	1099	-1	0,2588	2007	-1	0,2222	11499	1	-0,0366
LINC00592	7924	0,0650	3327	-0,0764	3148	-0,1890	1156	-1	-0,2540	3913	-1	-0,1415	4776	1	0,1125
ASPSCR1	24707	0,0061	2987	-0,0827	2243	-0,2402	1253	-1	-0,2463	6412	-1	-0,0888	3100	1	0,1575
SLC16A13	25389	0,0053	3288	-0,0770	2355	-0,2320	1366	-1	-0,2374	6822	-1	-0,0824	3167	1	0,1550
CACNG8	11997	0,0344	4860	-0,0558	2903	-0,1997	1411	-1	-0,2341	6326	-1	-0,0903	3503	1	0,1439
MAOA	19967	0,0107	2848	-0,0852	2563	-0,2187	1483	-1	-0,2294	5982	-1	-0,0959	3865	1	0,1335
ZNF711	7761	0,0668	4238	-0,0629	4137	-0,1531	1642	-1	-0,2199	4324	-1	-0,1298	6030	1	0,0902
FMN1	2992	0,1534	410	-0,2161	9088	-0,0640	1682	-1	-0,2175	800	-1	-0,3695	3261	1	-0,1520
NUDT14	16746	0,0175	3145	-0,0798	2964	-0,1969	1741	-1	-0,2145	5873	-1	-0,0973	4537	1	0,1171
MKI67	2097	-0,1908	327	0,2371	15449	0,0232	1750	-1	0,2140	613	-1	0,4279	2044	1	0,2139
HOGA1	13973	0,0255	5741	-0,0471	3184	-0,1872	1781	-1	-0,2127	7536	-1	-0,0726	3624	1	0,1402
SLC16A7	5918	-0,0904	1330	0,1310	5370	0,1208	1802	-1	0,2113	2021	-1	0,2214	20216	1	0,0101
ANKRD36C	18153	-0,0141	25684	0,0015	2975	0,1964	1813	-1	0,2104	17000	-1	0,0156	2319	1	-0,1948
ADA	2836	0,1586	2556	-0,0916	10769	-0,0485	1869	-1	-0,2071	1645	-1	-0,2502	10515	1	-0,0431
FHL1	26072	0,0039	129	-0,3418	2865	-0,2018	1898	-1	-0,2057	907	-1	-0,3457	3628	1	-0,1400
FRMD4B	20018	0,0103	5463	-0,0498	3129	-0,1897	2028	-1	-0,2000	8649	-1	-0,0601	3632	1	0,1399
BASP1	6622	0,0804	261	-0,2570	5744	-0,1129	2183	-1	-0,1933	952	-1	-0,3374	3498	1	-0,1440
SAP30L-AS1	14185	-0,0241	6023	0,0447	3724	0,1665	2242	-1	0,1907	7859	-1	0,0688	4326	1	-0,1219
GRB2	9388	0,0513	1286	-0,1333	4683	-0,1374	2287	-1	-0,1887	2698	-1	-0,1845	24532	1	0,0041
STXBP5L	22387	-0,0071	4763	0,0569	3430	0,1780	2372	-1	0,1850	8290	-1	0,0640	4361	1	-0,1211
MST4	7304	0,0720	839	-0,1616	5886	-0,1100	2436	-1	-0,1820	1844	-1	-0,2336	9388	1	-0,0516
RP11-443A13.5	10226	-0,0450	9199	0,0235	4731	0,1362	2450	-1	0,1813	7885	-1	0,0685	4765	1	-0,1127
ZNF468	25314	-0,0058	3294	0,0770	3560	0,1730	2513	-1	0,1788	6795	-1	0,0828	5657	1	-0,0960
RP11-214O1.2	25954	-0,0041	10632	0,0178	3521	0,1744	2519	-1	0,1785	14719,5	-1	0,0219	3123	1	-0,1566
PXDNL	13577	-0,0275	3653	0,0715	4304	0,1477	2589	-1	0,1753	5781	-1	0,0991	7028	1	-0,0762
TNFAIP3	2763	-0,1620	668	0,1775	20895	0,0120	2631	-1	0,1740	938	-1	0,3395	2915	1	0,1655
NR2F2	4030	-0,1244	383	0,2212	11347	0,0433	2815	-1	0,1677	909	-1	0,3456	2659	1	0,1779

P2RY6	28257	0,0003	835	-0,1620	3701	-0,1674	2817	-1	-0,1677	3256	-1	-0,1623	24040	1	0,0054
DOK4	6689	0,0794	3917	-0,0675	7197	-0,0881	2822	-1	-0,1675	3725	-1	-0,1469	15305	1	0,0205
RUNX2	7134	-0,0738	328	0,2367	7481	0,0843	3109	-1	0,1581	1110	-1	0,3105	3254	1	0,1523
BLID	19201	-0,0116	12876	0,0115	4726	0,1364	3467	-1	0,1479	14346,5	-1	0,0231	4187,5	1	-0,1248
NABP1	28259	-0,0003	210	0,2780	4741	0,1360	3908	-1	0,1363	1363	-1	0,2783	3564	1	0,1420
ZNF736	18079	-0,0149	4971	0,0547	5381	0,1207	3950	-1	0,1355	7794	-1	0,0695	7862	1	-0,0660
HNRNPU-AS1	4769	-0,1086	5504	0,0493	15596	0,0224	4150	-1	0,1310	3386,5	-1	0,1579	13395	1	0,0269
RPL9P28	24323	-0,0066	19976	0,0039	5484	0,1184	4400	-1	0,1250	19298,5	-1	0,0105	4674	1	-0,1145
RAB42	25791	0,0041	4790	-0,0566	5415	-0,1199	4449	-1	-0,1240	8592	-1	-0,0607	8117	1	0,0633
ANKRD36	12557	-0,0310	9425	0,0226	7062	0,0902	4589	-1	0,1212	9296	-1	0,0536	7742	1	-0,0675
B3GNT3	11451	0,0370	6769	-0,0384	7756	-0,0806	4776	-1	-0,1176	7299	-1	-0,0753	10631,5	1	0,0422
NTRK3-AS1	16840	-0,0168	4568	0,0590	7216	0,0878	5457,5	-1	0,1045	7275	-1	0,0758	13033	1	-0,0287
SDK1	5428	-0,0977	1109	0,1429	27310	0,0013	5796	-1	0,0990	1757	-1	0,2406	3573	1	0,1417
PID1	4923	-0,1058	3111	-0,0805	561	0,5454	97	-1	0,6512	13796	1	0,0254	317	-1	-0,6259
GALNT16	14154	-0,0244	15592	-0,0074	569	0,5438	147	-1	0,5682	16458	1	0,0171	430	-1	-0,5512
OLFML2A	12380	0,0320	12355	0,0129	915	-0,4222	264	-1	-0,4542	15544	1	-0,0191	671	-1	0,4351
RARB	5303	0,0997	2345	0,0966	8272	-0,0737	2645	-1	-0,1735	27899	1	-0,0031	2815	-1	0,1704
CLDN2	6175	0,0865	40	0,4855	7967	-0,0776	2913	-1	-0,1641	702	1	0,3991	409	-1	0,5632
RP11-115C10.1	11756	-0,0358	27827	-0,0004	5971	0,1082	3623	-1	0,1440	11708	1	0,0353	4959	-1	-0,1087
MEGF6	8594	0,0580	254	0,2595	16734	-0,0190	7419	-1	-0,0770	2366	1	0,2015	1400	-1	0,2785
CNR1	11414	0,0372	1285	0,1333	20931	-0,0116	10247	-1	-0,0488	5971	1	0,0961	3475	-1	0,1449
CXCL5	25	-1,1584	24	0,5609	1616	-0,2968	33	1	0,8616	2	-1	1,7193	127	-1	0,8577
CD300C	5357	0,0989	17635	-0,0052	175	0,8760	52	1	0,7770	5498	-1	-0,1042	117	-1	-0,8812
JUP	115	0,7492	4	-1,0602	13049	0,0328	68	1	-0,7164	1	-1	-1,8095	55	-1	-1,0930
SEMA7A	204	0,6386	8958	-0,0247	40	1,3275	79	1	0,6889	219	-1	-0,6633	25	-1	-1,3522
RET	6433	0,0827	13887	-0,0098	296	0,7163	106	1	0,6336	6192	-1	-0,0925	223	-1	-0,7261
SLFN5	130	0,7261	845	-0,1609	6450	0,1000	112	1	-0,6261	82	-1	-0,8870	1548	-1	-0,2609
HAPLN3	189	0,6602	810	-0,1635	8574	0,0702	134	1	-0,5900	111	-1	-0,8237	1824	-1	-0,2337
SYT1	216	0,6271	56	-0,4542	6635	0,0973	178	1	-0,5298	38	-1	-1,0812	428	-1	-0,5514
WNT7A	170	0,6824	1376	-0,1293	2829	0,2034	234	1	-0,4790	120	-1	-0,8117	1067	-1	-0,3327
THBS1	52	0,9248	5331	-0,0510	38	1,3676	283	1	0,4427	57	-1	-0,9758	18	-1	-1,4185
SPHK1	50	0,9543	6584	-0,0399	35	1,3954	285	1	0,4411	53	-1	-0,9943	17	-1	-1,4353
XYLT1	13	1,3472	2446	-0,0942	157	0,9131	299	1	-0,4340	16	-1	-1,4414	70	-1	-1,0074

GPAM	3572	0,1362	10316	-0,0191	532	0,5556	327	1	0,4194	3474	-1	-0,1552	386	-1	-0,5746
ANGPTL4	15	1,3162	3608	-0,0723	11	1,7263	347	1	0,4101	17	-1	-1,3885	6	-1	-1,7986
KLHDC8A	4450	0,1155	25805	-0,0014	615	0,5210	356	1	0,4055	4882	-1	-0,1169	475	-1	-0,5224
NKD1	2100	0,1907	21976	-0,0032	481	0,5823	383	1	0,3916	2516	-1	-0,1939	366	-1	-0,5855
PRDM1	234	0,6079	935	-0,1535	2345	0,2326	434	1	-0,3753	151	-1	-0,7614	821	-1	-0,3862
FOXS1	369	0,5041	20217	-0,0034	174	0,8786	440	1	0,3745	425	-1	-0,5075	116	-1	-0,8820
AKR1C1	64	-0,8596	3979	0,0666	57	-1,2317	446	1	-0,3721	71	-1	0,9261	32	-1	1,2982
SLC19A2	608	0,3940	8498	-0,0271	253	0,7639	454	1	0,3699	630	-1	-0,4211	171	-1	-0,7910
RHOBTB3	645	-0,3826	357	0,2269	16125	-0,0218	486	1	0,3609	271	-1	0,6096	1651	-1	0,2487
GFRA1	308	-0,5469	678	0,1765	3066	-0,1922	508	1	0,3548	178	-1	0,7234	901	-1	0,3687
STRA6	332	0,5250	116	-0,3546	3543	0,1736	516	1	-0,3514	87	-1	-0,8796	465	-1	-0,5282
JAG1	107	0,7629	1450	-0,1261	71	1,1123	528	1	0,3494	81	-1	-0,8890	35	-1	-1,2384
KBTBD11	1006	0,2991	8092	-0,0294	381	0,6425	549	1	0,3435	1002	-1	-0,3284	275	-1	-0,6719
MEG3	42	0,9882	2178	-0,1011	376	0,6487	556	1	-0,3395	37	-1	-1,0893	202	-1	-0,7499
FN1	40	0,9963	12	-0,7143	358	0,6592	569	1	-0,3371	4	-1	-1,7107	24	-1	-1,3735
TCF4	1417	0,2466	8267	-0,0285	510	0,5677	643	1	0,3211	1395	-1	-0,2750	351	-1	-0,5962
GSN	292	0,5543	251	-0,2602	2222	0,2417	690	1	-0,3126	115	-1	-0,8145	511	-1	-0,5019
LINC00704	197	0,6493	7201	-0,0350	1312	0,3372	691	1	-0,3121	206	-1	-0,6843	882	-1	-0,3722
RP11-572C15.6	16	1,2885	5872	-0,0459	23	1,5946	731	1	0,3061	19	-1	-1,3344	10	-1	-1,6406
CEBPD	2276	-0,1818	5863	0,0459	702	-0,4878	732	1	-0,3060	1930	-1	0,2278	458	-1	0,5337
STARD4	442	0,4636	165	-0,3100	3987	0,1580	734	1	-0,3056	144	-1	-0,7736	579	-1	-0,4680
AKR1B15	908	-0,3185	6032	0,0446	422	-0,6150	787	1	-0,2965	826	-1	0,3631	287	-1	0,6596
GRAMD1B	513	0,4269	59	-0,4492	4926	0,1310	790	1	-0,2958	88	-1	-0,8760	375	-1	-0,5802
SOGA2	2497	-0,1719	2919	0,0840	774	-0,4613	837	1	-0,2895	1589	-1	0,2558	435	-1	0,5453
PIK3AP1	195	0,6529	4646	-0,0581	1175	0,3648	854	1	-0,2880	188	-1	-0,7110	710	-1	-0,4229
LDLRAD4	133	0,7226	14698	-0,0086	111	1,0093	863	1	0,2867	169	-1	-0,7311	68	-1	-1,0179
PTK7	1157	0,2756	14699	-0,0086	517	0,5610	871	1	0,2854	1306	-1	-0,2842	398	-1	-0,5696
TNFAIP6	32	1,0605	1198	-0,1382	244	0,7754	874	1	-0,2851	25	-1	-1,1987	106	-1	-0,9136
MOK	435	-0,4673	43	0,4810	3284	-0,1832	880	1	0,2841	64	-1	0,9483	281	-1	0,6642
DGKA	692	0,3689	738	-0,1701	7147	0,0889	908	1	-0,2801	370	-1	-0,5390	1563	-1	-0,2590
ARID5A	3295	0,1438	14425	-0,0091	911	0,4228	918	1	0,2790	3537	-1	-0,1528	686	-1	-0,4318
IGFBP7	83	0,8137	201	-0,2828	586	0,5356	928	1	-0,2781	31	-1	-1,0965	155	-1	-0,8183
WNT7B	94	-0,7896	16830	0,0065	641	-0,5115	930	1	0,2781	126	-1	0,7962	487	-1	0,5181

SEL1L3	239	0,6024	7551	-0,0327	1361	0,3312	987	1	-0,2712	234	-1	-0,6351	923	-1	-0,3639
EFHD2	90	-0,7974	1800	0,1120	593	-0,5311	1034	1	0,2663	78	-1	0,9094	304	-1	0,6432
CYP1B1	179	-0,6678	120	0,3534	1004	-0,4023	1039	1	0,2655	47	-1	1,0212	196	-1	0,7556
ITGA1	78	0,8185	17336	-0,0056	523	0,5585	1091	1	-0,2600	110	-1	-0,8241	408	-1	-0,5641
PGM2L1	178	0,6702	126	-0,3441	956	0,4141	1127	1	-0,2561	49	-1	-1,0143	191	-1	-0,7582
KCNJ6	1044	0,2927	13019	-0,0112	11996	0,0391	1163	1	-0,2535	1145	-1	-0,3039	9567,5	-1	-0,0504
PLEK2	98	0,7819	3749	-0,0702	102	1,0327	1191	1	0,2508	101	-1	-0,8521	53	-1	-1,1029
NLRP1	106	0,7634	20048	-0,0038	637	0,5131	1202	1	-0,2502	149	-1	-0,7671	489	-1	-0,5169
AHR	275	-0,5649	22508	0,0023	211	-0,8126	1229	1	-0,2477	322	-1	0,5673	157	-1	0,8150
GPR115	159	0,6923	2708	-0,0878	842	0,4451	1238	1	-0,2471	140	-1	-0,7801	460	-1	-0,5329
RNF152	6588	0,0809	24997	-0,0020	1390	0,3271	1256	1	0,2462	6785,5	-1	-0,0829	1090	-1	-0,3291
NDRG1	713	0,3620	563	-0,1907	5368	0,1209	1309	1	-0,2412	351	-1	-0,5527	1183	-1	-0,3115
BMF	633	0,3870	12755	-0,0119	408	0,6253	1357	1	0,2383	703	-1	-0,3989	306	-1	-0,6371
PCSK9	6338	-0,0842	12972	0,0114	1428	-0,3213	1370	1	-0,2371	6002	-1	0,0955	1068	-1	0,3326
CSF1	212	-0,6310	1050	0,1457	1032	-0,3954	1387	1	0,2356	141	-1	0,7767	445	-1	0,5411
DOCK4	81	0,8145	1788	-0,1123	95	1,0479	1424	1	0,2334	70	-1	-0,9268	43	-1	-1,1602
STC1	38	1,0163	1145	-0,1410	234	0,7834	1433	1	-0,2329	28	-1	-1,1573	98	-1	-0,9244
APBB2	463	0,4473	28102	0,0000	336	0,6773	1476	1	0,2300	554	-1	-0,4473	270	-1	-0,6773
NFKBIZ	158	0,6948	1755	-0,1136	150	0,9242	1484	1	0,2294	122	-1	-0,8084	63	-1	-1,0378
NEK7	367	0,5083	10452	-0,0185	276	0,7362	1509	1	0,2279	386	-1	-0,5268	200	-1	-0,7547
SH3RF2	1374	-0,2503	301	0,2446	15425	-0,0234	1527	1	0,2269	444	-1	0,4949	1488	-1	0,2680
CLDN1	174	-0,6768	128	0,3418	812	-0,4519	1559	1	0,2249	48	-1	1,0187	168	-1	0,7937
MLXIP	75	0,8225	5963	-0,0451	97	1,0472	1568	1	0,2246	91	-1	-0,8676	56	-1	-1,0923
MAF	236	0,6049	11307	-0,0156	1080	0,3818	1588	1	-0,2231	257	-1	-0,6205	782	-1	-0,3974
LGMN	164	0,6865	1467	-0,1251	766	0,4639	1599	1	-0,2225	121	-1	-0,8116	361	-1	-0,5891
LMCD1	499	0,4351	2696	-0,0881	361	0,6574	1608	1	0,2223	393	-1	-0,5233	206	-1	-0,7456
AJUBA	70	-0,8409	186	0,2971	419	-0,6187	1610	1	0,2222	29	-1	1,1380	102	-1	0,9158
PARD6B	573	-0,4073	4543	0,0594	402	-0,6294	1615	1	-0,2220	513	-1	0,4668	257	-1	0,6888
TRNP1	66	-0,8468	3185	0,0790	409	-0,6249	1620	1	0,2219	72	-1	0,9258	247	-1	0,7039
GAL3ST1	5887	-0,0908	10129	0,0197	1489	-0,3121	1625	1	-0,2213	5188	-1	0,1106	1073	-1	0,3318
PHF15	439	-0,4658	4218	0,0631	333	-0,6829	1688	1	-0,2170	383	-1	0,5289	205	-1	0,7460
WNT5B	2	1,6842	24690	-0,0022	29	1,4692	1731	1	-0,2150	5	-1	-1,6864	15	-1	-1,4715
LMNA	4221	-0,1202	6325	0,0419	1332	-0,3348	1739	1	-0,2146	3261	-1	0,1621	861	-1	0,3767

AL132709.5	173	0,6786	8349	-0,0280	758	0,4676	1805	1	-0,2110	191	-1	-0,7066	522	-1	-0,4956
RP11-401P9.4	7577	0,0691	11731	-0,0144	1782	0,2800	1808	1	0,2110	6751	-1	-0,0834	1290	-1	-0,2944
RAB11FIP1	1019	-0,2973	1922	0,1086	650	-0,5058	1845	1	-0,2086	674	-1	0,4058	328	-1	0,6144
CCDC93	1419	0,2465	21965	-0,0032	805	0,4538	1863	1	0,2073	1649	-1	-0,2497	613	-1	-0,4570
COTL1	630	-0,3877	684	0,1756	3252	-0,1843	1935	1	0,2034	330	-1	0,5634	945	-1	0,3600
PEG10	131	-0,7255	11997	0,0137	146	-0,9275	1973	1	-0,2020	162	-1	0,7392	92	-1	0,9412
ITPKB	357	0,5137	4204	-0,0634	297	0,7152	1987	1	0,2015	310	-1	-0,5770	178	-1	-0,7786
RHOU	20	1,2378	241	-0,2644	100	1,0370	2003	1	-0,2008	13	-1	-1,5022	31	-1	-1,3015
BAZ2B	501	0,4349	2015	-0,1054	2291	0,2364	2065	1	-0,1985	366	-1	-0,5403	1028	-1	-0,3418
CEP250	3197	-0,1462	6185	0,0431	1276	-0,3444	2071	1	-0,1982	2598	-1	0,1894	812	-1	0,3875
TOM1	585	0,4015	4251	-0,0628	446	0,5979	2106	1	0,1964	520	-1	-0,4643	284	-1	-0,6607
SLC4A7	359	0,5124	2843	-0,0853	1450	0,3182	2165	1	-0,1941	279	-1	-0,5977	762	-1	-0,4036
GADD45B	337	0,5227	8654	-0,0263	295	0,7166	2170	1	0,1939	355	-1	-0,5489	210	-1	-0,7428
RP4-555D20.2	200	-0,6444	236	0,2662	817	-0,4510	2180	1	0,1934	77	-1	0,9106	230	-1	0,7172
CACNA1I	485	0,4398	8969	-0,0247	398	0,6324	2196	1	0,1926	519	-1	-0,4645	289	-1	-0,6570
ZSWIM6	4569	0,1126	11180	-0,0160	1559	0,3041	2223	1	0,1915	4365	-1	-0,1286	1130	-1	-0,3201
CTPS1	994	0,3014	8146	-0,0291	694	0,4909	2269	1	0,1895	987	-1	-0,3305	483	-1	-0,5200
MORC4	448	-0,4594	1900	0,1092	375	-0,6487	2271	1	-0,1894	320	-1	0,5686	192	-1	0,7579
N4BP2	4856	0,1072	27811	-0,0005	1638	0,2947	2316	1	0,1875	5334	-1	-0,1077	1289	-1	-0,2952
FOXP1-IT1	2067	0,1921	11137	-0,0162	1095	0,3789	2336	1	0,1867	2254	-1	-0,2083	789	-1	-0,3951
FARP1	1066	0,2901	14411	-0,0092	742	0,4741	2396	1	0,1840	1177	-1	-0,2992	554	-1	-0,4832
SH3BP5L	416	0,4748	17136	-0,0059	362	0,6574	2422	1	0,1826	476	-1	-0,4808	283	-1	-0,6633
NPY4R	637	-0,3859	3159	0,0795	508	-0,5681	2431	1	-0,1821	515	-1	0,4654	299	-1	0,6476
FAM43A	1168	0,2743	1863	-0,1102	6949	0,0921	2433	1	-0,1821	748	-1	-0,3845	2205	-1	-0,2024
DDB2	723	0,3600	955	-0,1519	573	0,5417	2440	1	0,1817	411	-1	-0,5118	255	-1	-0,6936
PLCXD2	116	0,7490	8791	-0,0256	507	0,5683	2458	1	-0,1807	143	-1	-0,7746	355	-1	-0,5938
IMPA2	3468	-0,1390	3538	0,0734	1449	-0,3184	2488	1	-0,1794	2186	-1	0,2124	797	-1	0,3917
FURIN	256	0,5829	313	-0,2403	258	0,7602	2546	1	0,1773	112	-1	-0,8232	72	-1	-1,0004
KLHDC7A	1204	-0,2698	5694	0,0475	840	-0,4453	2583	1	-0,1755	1059	-1	0,3173	528	-1	0,4928
IGFL3	296	0,5515	13443	-0,0104	1106	0,3765	2598	1	-0,1750	334	-1	-0,5619	814	-1	-0,3869
CYP26B1	669	0,3756	7932	-0,0303	553	0,5486	2651	1	0,1731	675	-1	-0,4058	378	-1	-0,5789
ARFGAP1	500	0,4350	11491	-0,0150	432	0,6080	2654	1	0,1730	548	-1	-0,4500	319	-1	-0,6230
PDGFB	51	0,9429	9440	-0,0226	69	1,1154	2669	1	0,1725	59	-1	-0,9655	46	-1	-1,1380

CCDC80	14	1,3311	660	-0,1782	27	1,5031	2682	1	0,1721	11	-1	-1,5093	8	-1	-1,6814
CSRNP1	330	0,5263	4733	-0,0572	318	0,6959	2752	1	0,1696	299	-1	-0,5835	201	-1	-0,7531
OCIAD2	808	0,3421	1092	-0,1437	642	0,5114	2762	1	0,1693	467	-1	-0,4858	293	-1	-0,6551
TCEAL1	190	0,6588	8306	-0,0282	693	0,4909	2807	1	-0,1679	202	-1	-0,6871	485	-1	-0,5192
BIRC3	59	-0,8858	3019	0,0821	290	-0,7192	2849	1	0,1666	58	-1	0,9679	165	-1	0,8013
RP1-27K12.4	3210	0,1459	18546	-0,0048	1533	0,3062	3033	1	0,1603	3596	-1	-0,1507	1188	-1	-0,3110
SERPINE2	27	1,1284	2138	-0,1020	128	0,9682	3034	1	-0,1602	24	-1	-1,2304	59	-1	-1,0702
UBA6	352	0,5158	14985	-0,0079	339	0,6752	3070	1	0,1593	391	-1	-0,5238	265	-1	-0,6831
DHRS2	252	0,5846	4500	-0,0599	272	0,7433	3095	1	0,1586	231	-1	-0,6445	163	-1	-0,8032
SMTN	208	0,6362	5621	-0,0482	224	0,7938	3123	1	0,1576	205	-1	-0,6844	138	-1	-0,8420
UAP1	1242	0,2646	9424	-0,0226	930	0,4192	3220	1	0,1546	1283	-1	-0,2872	654	-1	-0,4418
ABHD2	127	0,7299	4969	-0,0547	491	0,5754	3225	1	-0,1544	136	-1	-0,7846	313	-1	-0,6302
MYH16	349	0,5163	17469	-0,0054	347	0,6686	3298	1	0,1523	397	-1	-0,5217	272	-1	-0,6739
MYOF	187	-0,6605	2994	0,0826	212	-0,8115	3353	1	-0,1511	158	-1	0,7430	110	-1	0,8941
RFTN1	400	0,4820	6557	-0,0402	399	0,6314	3410	1	0,1494	396	-1	-0,5222	276	-1	-0,6716
RP1-60N8.1	10511	0,0429	27939	-0,0003	3071	0,1919	3425	1	0,1490	10508	-1	-0,0432	2370	-1	-0,1922
SIRPA	102	0,7692	9313	-0,0231	155	0,9177	3451	1	0,1485	128	-1	-0,7923	93	-1	-0,9408
FNDC3A	262	0,5745	1993	-0,1060	899	0,4267	3473	1	-0,1478	210	-1	-0,6805	462	-1	-0,5327
LPAR5	491	0,4382	24926	-0,0020	1681	0,2905	3476	1	-0,1477	580	-1	-0,4403	1300	-1	-0,2926
ACSL4	814	0,3407	3698	-0,0708	704	0,4870	3529	1	0,1463	656	-1	-0,4115	421	-1	-0,5579
RP1-86D1.5	5692	0,0935	14290	-0,0093	2258	0,2388	3568	1	0,1453	5584	-1	-0,1028	1659	-1	-0,2482
MRC2	84	0,8116	26469	-0,0011	133	0,9565	3593	1	0,1448	118	-1	-0,8127	87	-1	-0,9576
RFFL	6680	-0,0796	10056	0,0200	2477	-0,2236	3619	1	-0,1440	5753	-1	0,0996	1708	-1	0,2436
STXBP5	302	0,5489	7342	-0,0341	994	0,4052	3637	1	-0,1437	300	-1	-0,5830	659	-1	-0,4393
FAT4	2803	-0,1601	615	0,1836	17220	-0,0173	3670	1	0,1427	918	-1	0,3436	2227	-1	0,2009
MCTP1	1789	-0,2118	1272	0,1339	8670	-0,0691	3672	1	0,1427	906	-1	0,3457	2188	-1	0,2031
RP11-154H23.3	2726	0,1632	9761	-0,0212	1550	0,3053	3694	1	0,1421	2705	-1	-0,1843	1099	-1	-0,3264
CGB5	7318	0,0720	22895	-0,0023	2665	0,2124	3746	1	0,1404	7405,5	-1	-0,0743	2035	-1	-0,2147
CHRNA9	214	0,6287	4292	-0,0623	247	0,7690	3753	1	0,1403	197	-1	-0,6910	144	-1	-0,8314
DIRC3	18945	0,0123	17700	-0,0052	4165	0,1523	3774	1	0,1400	16283,5	-1	-0,0176	3097	-1	-0,1576
CGB8	2369	0,1774	15677	-0,0073	1459	0,3165	3801	1	0,1391	2695	-1	-0,1847	1107	-1	-0,3238
TGFBR1	147	0,7109	472	-0,2044	496	0,5739	3874	1	-0,1370	76	-1	-0,9154	179	-1	-0,7784
IGFBP6	756	-0,3525	3403	0,0753	703	-0,4872	3984	1	-0,1346	614	-1	0,4278	413	-1	0,5625

SNHG14	3931	0,1266	8980	-0,0246	1971	0,2608	4010	1	0,1341	3581	-1	-0,1513	1348	-1	-0,2854
MTSS1L	639	-0,3846	1540	0,1218	2089	-0,2507	4019	1	0,1339	428	-1	0,5064	880	-1	0,3725
SLC39A10	826	0,3389	1391	-0,1285	2806	0,2052	4022	1	-0,1338	509	-1	-0,4675	1063	-1	-0,3337
PCDH1	436	0,4671	10386	-0,0188	442	0,5994	4091	1	0,1323	465	-1	-0,4859	322	-1	-0,6182
PPP1R15A	3807	-0,1304	4756	0,0570	1961	-0,2617	4137	1	-0,1314	2647	-1	0,1873	1139	-1	0,3187
ELFN2	942	-0,3095	628	0,1824	3402	-0,1792	4181	1	0,1303	451	-1	0,4918	938	-1	0,3615
PWAR6	3987	0,1254	11998	-0,0137	2036	0,2551	4199	1	0,1297	3993	-1	-0,1391	1475	-1	-0,2688
CHD9	776	0,3471	475	-0,2042	2573	0,2178	4221	1	-0,1293	353	-1	-0,5513	715	-1	-0,4220
MIR31HG	897	0,3201	6571	-0,0401	827	0,4486	4258	1	0,1285	837	-1	-0,3602	542	-1	-0,4887
PAQR5	185	-0,6613	2373	0,0959	589	-0,5333	4272	1	0,1280	153	-1	0,7572	314	-1	0,6292
TREH	570	0,4077	17450	-0,0054	1784	0,2799	4284	1	-0,1278	652	-1	-0,4131	1350	-1	-0,2853
CES2	3320	-0,1432	20098	0,0036	1856	-0,2710	4285	1	-0,1278	3735	-1	0,1468	1425	-1	0,2745
MAFG-AS1	8227	-0,0621	5748	0,0470	3183	-0,1874	4388	1	-0,1254	5265	-1	0,1091	1812	-1	0,2345
PLXND1	1122	0,2810	92	-0,3820	3865	0,1618	4674	1	-0,1192	221	-1	-0,6630	441	-1	-0,5438
JHDM1D	775	0,3471	3716	-0,0706	759	0,4662	4678	1	0,1191	638	-1	-0,4177	450	-1	-0,5368
SRC	739	-0,3566	2358	0,0963	2267	-0,2380	4710	1	0,1186	543	-1	0,4529	1062	-1	0,3343
RP11-383H13.1	4277	0,1189	14748	-0,0083	2283	0,2372	4729	1	0,1183	4428	-1	-0,1272	1679	-1	-0,2455
BHLHE40	427	0,4706	72	-0,4118	1223	0,3535	4799	1	-0,1170	85	-1	-0,8824	186	-1	-0,7654
SQSTM1	1304	-0,2575	7111	0,0357	1125	-0,3732	4869	1	-0,1157	1223	-1	0,2932	751	-1	0,4089
MPV17L	977	-0,3039	10235	0,0194	931	-0,4190	4896	1	-0,1151	1025	-1	0,3233	663	-1	0,4384
TRIM16	540	-0,4173	8497	0,0271	1567	-0,3029	4928	1	0,1144	563	-1	0,4444	1086	-1	0,3300
LINC00473	2339	-0,1786	2786	0,0862	8976	-0,0652	4976	1	0,1135	1498	-1	0,2648	3286	-1	0,1513
CGB7	950	0,3076	25764	-0,0014	924	0,4195	5062	1	0,1119	1121	-1	-0,3091	719	-1	-0,4210
MEG8	297	0,5506	6107	-0,0438	849	0,4419	5243	1	-0,1087	284	-1	-0,5944	551	-1	-0,4858
GAL	153	0,7006	6112	-0,0438	218	0,8055	5440	1	0,1049	156	-1	-0,7444	133	-1	-0,8493
DOCK4-AS1	1561	0,2316	9302	-0,0231	1338	0,3337	5602	1	0,1021	1598	-1	-0,2547	958	-1	-0,3568
ZNF185	29	1,1076	848	-0,1608	112	1,0062	5642	1	-0,1015	23	-1	-1,2684	41	-1	-1,1670
WSCD1	5155	0,1019	2072	-0,1040	2834	0,2031	5655	1	0,1012	2289	-1	-0,2059	1211	-1	-0,3072
PRR5L	326	0,5294	6890	-0,0375	897	0,4291	5712	1	-0,1003	324	-1	-0,5669	582	-1	-0,4666
AC003080.4	8442	0,0595	22175	-0,0028	3938	0,1595	5733	1	0,1000	8446	-1	-0,0624	2985	-1	-0,1623
LINC00312	5073	0,1031	11494	-0,0150	2837	0,2030	5736	1	0,1000	4830	-1	-0,1181	2004	-1	-0,2180
BMPER	1681	-0,2197	1218	0,1371	5363	-0,1210	5818	1	0,0986	855	-1	0,3568	1570	-1	0,2581
KANK4	145	0,7115	8999	-0,0245	214	0,8095	5853	1	0,0981	166	-1	-0,7360	142	-1	-0,8340

MBOAT2	62	0,8730	7795	-0,0311	125	0,9706	5888	1	0,0976	79	-1	-0,9041	71	-1	-1,0017
PLXNA4	314	0,5418	17007	-0,0062	388	0,6389	5921	1	0,0971	357	-1	-0,5479	303	-1	-0,6450
DUSP5	2583	-0,1686	7270	0,0346	8312	-0,0729	6008	1	0,0957	2342	-1	0,2032	5025	-1	0,1075
GPR64	3726	-0,1323	17377	0,0055	2411	-0,2273	6053	1	-0,0950	4037	-1	0,1378	1835	-1	0,2328
MFAP3L	160	0,6908	1017	-0,1478	451	0,5964	6094	1	-0,0944	105	-1	-0,8387	209	-1	-0,7443
RIPK4	1695	-0,2187	3336	0,0763	1488	-0,3121	6160	1	-0,0934	1209	-1	0,2950	808	-1	0,3885
AIFM2	3423	-0,1403	8089	0,0294	2347	-0,2325	6226	1	-0,0922	3068	-1	0,1697	1536	-1	0,2619
ADAMTS15	512	0,4272	5132	-0,0529	1324	0,3351	6231	1	-0,0921	479	-1	-0,4801	811	-1	-0,3879
DRAP1	2724	-0,1632	5769	0,0468	2033	-0,2551	6250	1	-0,0919	2227	-1	0,2100	1242	-1	0,3019
PYGB	682	-0,3724	7335	0,0342	1776	-0,2810	6288	1	0,0914	671	-1	0,4065	1154	-1	0,3151
ACSS1	842	-0,3341	1033	0,1466	2201	-0,2427	6290	1	0,0914	477	-1	0,4807	805	-1	0,3893
DIXDC1	171	0,6804	2145	-0,1017	246	0,7715	6306	1	0,0911	137	-1	-0,7821	120	-1	-0,8732
GSE1	261	-0,5751	964	0,1515	713	-0,4843	6314	1	0,0909	174	-1	0,7267	308	-1	0,6358
PLEKHG4	697	-0,3659	1459	0,1255	802	-0,4543	6513	1	-0,0884	452	-1	0,4914	376	-1	0,5798
CTC-340I23.2	9344	0,0514	14336	-0,0092	4587	0,1398	6517	1	0,0884	8598,5	-1	-0,0606	3342	-1	-0,1490
IGSF9	2512	0,1713	8901	-0,0251	1991	0,2586	6597	1	0,0873	2455	-1	-0,1964	1367	-1	-0,2837
SMAD6	741	-0,3566	5977	0,0450	1874	-0,2697	6624	1	0,0869	691	-1	0,4016	1159	-1	0,3147
MIR181A1HG	13335	0,0277	12663	-0,0120	5673	0,1144	6645	1	0,0867	10975	-1	-0,0397	4114	-1	-0,1265
OLFM2	454	0,4531	9303	-0,0231	576	0,5397	6649	1	0,0866	484	-1	-0,4762	410	-1	-0,5628
RP11-554I8.2	730	-0,3587	10413	0,0187	841	-0,4453	6656	1	-0,0865	770	-1	0,3774	591	-1	0,4640
SKIL	321	0,5330	449	-0,2086	416	0,6191	6688	1	0,0861	159	-1	-0,7417	147	-1	-0,8277
AMIGO2	273	0,5659	6070	-0,0441	731	0,4802	6715	1	-0,0857	268	-1	-0,6100	472	-1	-0,5244
R3HDML	347	0,5183	23546	-0,0023	880	0,4327	6719	1	-0,0856	400	-1	-0,5206	672	-1	-0,4350
ETV4	2741	0,1628	529	-0,1960	7992	0,0774	6727	1	-0,0854	846	-1	-0,3588	1433	-1	-0,2734
P2RY2	415	0,4753	300	-0,2447	1050	0,3901	6753	1	-0,0852	180	-1	-0,7200	309	-1	-0,6349
INSIG1	1069	0,2900	2868	-0,0848	1120	0,3740	6846	1	0,0840	780	-1	-0,3748	608	-1	-0,4588
TENC1	683	-0,3723	1243	0,1353	1700	-0,2888	6884	1	0,0835	424	-1	0,5076	707	-1	0,4241
TP53I3	232	0,6081	1254	-0,1350	603	0,5249	6906	1	-0,0831	157	-1	-0,7430	286	-1	-0,6599
LSAMP	1816	0,2093	9484	-0,0224	1678	0,2909	7031	1	0,0816	1874	-1	-0,2316	1170	-1	-0,3133
FSTL3	291	0,5546	13703	-0,0101	395	0,6354	7099	1	0,0809	328	-1	-0,5646	301	-1	-0,6455
LRRC8C	285	0,5594	3417	-0,0751	389	0,6382	7266	1	0,0788	235	-1	-0,6345	234	-1	-0,7133
GABRE	1542	-0,2332	487	0,2027	1505	-0,3106	7387	1	-0,0774	592	-1	0,4359	496	-1	0,5133
SMAD3	63	-0,8629	549	0,1929	230	-0,7858	7413	1	0,0771	40	-1	1,0558	79	-1	0,9787

KLF13	990	0,3021	945	-0,1530	2421	0,2265	7536	1	-0,0756	538	-1	-0,4551	849	-1	-0,3795
EXTL2	1426	0,2455	11580	-0,0148	1435	0,3204	7570	1	0,0750	1542	-1	-0,2603	1057	-1	-0,3353
PLIN2	737	-0,3571	1055	0,1456	885	-0,4318	7591	1	-0,0747	433	-1	0,5027	382	-1	0,5773
LOX	953	0,3072	6907	-0,0373	2346	0,2326	7595	1	-0,0746	913	-1	-0,3445	1465	-1	-0,2699
CTD-2194F4.2	9725	0,0484	25773	-0,0014	5274	0,1229	7610,5	1	0,0745	9716,5	-1	-0,0499	4209	-1	-0,1244
FTL	601	-0,3956	2967	0,0831	1423	-0,3220	7690	1	0,0736	480	-1	0,4787	758	-1	0,4051
FAM83G	324	0,5297	1278	-0,1336	438	0,6016	7815	1	0,0719	220	-1	-0,6633	217	-1	-0,7352
LAMA5	1333	-0,2546	7385	0,0339	1412	-0,3235	8060	1	-0,0690	1271	-1	0,2884	956	-1	0,3574
SERPINE1	8	1,4941	387	-0,2200	32	1,4274	8276	1	-0,0667	3	-1	-1,7141	9	-1	-1,6474
CPT1A	1906	-0,2026	460	0,2063	1879	-0,2692	8291	1	-0,0666	663	-1	0,4089	563	-1	0,4755
CHRNB4	2566	0,1693	13844	-0,0098	2324	0,2344	8401	1	0,0652	2829	-1	-0,1791	1697	-1	-0,2443
NOVA2	999	0,3006	11963	-0,0137	2282	0,2373	8565	1	-0,0633	1079	-1	-0,3144	1627	-1	-0,2511
RP11-74C1.2	9886	0,0474	25167	-0,0020	5890	0,1099	8645,5	1	0,0625	9765	-1	-0,0494	4798,5	-1	-0,1119
RP11-215G15.5	738	-0,3567	2736	0,0872	1634	-0,2951	8746	1	0,0616	568	-1	0,4439	837	-1	0,3823
SCG5	748	0,3538	5897	-0,0457	950	0,4150	8797	1	0,0613	698	-1	-0,3994	600	-1	-0,4607
RP11-218E20.3	473	0,4445	10995	-0,0167	651	0,5053	8839	1	0,0608	527	-1	-0,4612	476	-1	-0,5220
P4HA3	34	1,0537	6290	-0,0423	116	0,9947	9063	1	-0,0590	32	-1	-1,0960	64	-1	-1,0370
TRIM16L	420	-0,4739	2608	0,0903	949	-0,4151	9087	1	0,0587	329	-1	0,5642	504	-1	0,5054
PKP2	288	-0,5589	1302	0,1324	657	-0,5011	9173	1	0,0577	196	-1	0,6913	310	-1	0,6336
HMOX1	3441	0,1398	374	-0,2232	7573	0,0833	9284	1	-0,0565	827	-1	-0,3630	1214	-1	-0,3065
FLOT1	831	-0,3372	26450	0,0012	1040	-0,3936	9299	1	-0,0564	946	-1	0,3384	792	-1	0,3948
SH3TC1	399	-0,4821	6101	0,0439	901	-0,4262	9348	1	0,0559	389	-1	0,5260	573	-1	0,4701
DDC8	2135	0,1891	13263	-0,0107	2168	0,2449	9354	1	0,0559	2394	-1	-0,1998	1590	-1	-0,2556
PLXNA2	301	0,5495	473	-0,2044	684	0,4938	9370	1	-0,0557	154	-1	-0,7539	250	-1	-0,6982
MAML3	313	0,5418	1601	-0,1193	706	0,4862	9380	1	-0,0556	223	-1	-0,6611	339	-1	-0,6055
UNC13D	11193	-0,0383	4453	0,0604	6866	-0,0936	9413	1	-0,0553	5809	-1	0,0986	3197	-1	0,1540
PDE11A	258	0,5800	8023	-0,0298	602	0,5251	9481	1	-0,0548	269	-1	-0,6098	426	-1	-0,5549
CNKSR3	1000	-0,2999	2627	0,0899	1219	-0,3546	9494	1	-0,0547	733	-1	0,3898	650	-1	0,4445
CTD-2066L21.3	198	0,6485	6505	-0,0406	452	0,5955	9683	1	-0,0530	201	-1	-0,6891	307	-1	-0,6361
JMJD1C	1137	0,2783	3449	-0,0747	2419	0,2266	9871	1	-0,0517	869	-1	-0,3531	1247	-1	-0,3014
KLF4	4045	-0,1242	12475	0,0125	3496	-0,1756	9906	1	-0,0514	4079	-1	0,1367	2444	-1	0,1881
RP11-519G16.5	2305	0,1801	24160	-0,0023	4999	0,1292	9964	1	-0,0509	2756	-1	-0,1824	3929	-1	-0,1315
VGLL3	55	0,9035	90	-0,3831	137	0,9531	10146	1	0,0496	22	-1	-1,2867	27	-1	-1,3363

KDM6B	37	1,0235	5637	-0,0481	87	1,0725	10220	1	0,0490	39	-1	-1,0716	49	-1	-1,1206
TSPAN9	372	0,5005	6248	-0,0426	552	0,5487	10326	1	0,0482	361	-1	-0,5432	358	-1	-0,5913
LMO4	1068	-0,2900	1642	0,1175	1305	-0,3380	10354	1	-0,0480	668	-1	0,4075	623	-1	0,4555
COL15A1	1036	0,2937	10003	-0,0201	2154	0,2460	10395	1	-0,0476	1082	-1	-0,3138	1507	-1	-0,2661
RP11-597K23.2	493	0,4378	20267	-0,0034	1048	0,3913	10554	1	-0,0464	576	-1	-0,4412	790	-1	-0,3948
PRKAB2	871	0,3269	11893	-0,0139	1128	0,3725	10660	1	0,0456	928	-1	-0,3408	818	-1	-0,3864
AP002954.3	1831	0,2082	25744	-0,0014	3793	0,1643	10890	1	-0,0439	2234	-1	-0,2096	2912	-1	-0,1657
RP11-460N11.2	15103	0,0213	18088	-0,0052	8989	0,0650	10908	1	0,0438	13528,5	-1	-0,0265	7493	-1	-0,0703
SPOCK1	60	0,8819	11170	-0,0160	151	0,9240	11153	1	0,0420	80	-1	-0,8979	94	-1	-0,9400
RELL1	91	0,7969	7198	-0,0350	262	0,7551	11198	1	-0,0418	107	-1	-0,8319	173	-1	-0,7901
OSGIN1	389	-0,4873	3442	0,0748	839	-0,4458	11263	1	0,0415	333	-1	0,5621	480	-1	0,5206
MT2A	269	0,5717	24627	-0,0023	596	0,5306	11323	1	-0,0411	313	-1	-0,5740	461	-1	-0,5329
TPCN1	868	-0,3274	25338	0,0018	1709	-0,2875	11479	1	0,0399	997	-1	0,3292	1316	-1	0,2893
BCL2L1	453	-0,4559	5145	0,0527	938	-0,4179	11760	1	0,0380	421	-1	0,5086	572	-1	0,4706
KLHL4	949	-0,3082	220	0,2735	1857	-0,2710	11897	1	0,0373	302	-1	0,5817	439	-1	0,5445
TNFAIP2	57	-0,8917	1948	0,1077	186	-0,8568	12407	1	0,0349	52	-1	0,9995	82	-1	0,9645
RPL9P16	8641	0,0578	19541	-0,0040	6939	0,0922	12526,5	1	0,0345	8498	-1	-0,0618	5642	-1	-0,0963
SEMA6B	351	-0,5162	125	0,3494	547	-0,5498	12711	1	-0,0336	92	-1	0,8656	109	-1	0,8992
C3	350	-0,5162	217	0,2752	719	-0,4836	12933	1	0,0325	130	-1	0,7914	189	-1	0,7589
MYLK	264	-0,5740	299	0,2449	433	-0,6064	12956	1	-0,0324	113	-1	0,8189	131	-1	0,8513
SGCD	167	0,6853	253	-0,2598	294	0,7171	13086	1	0,0318	65	-1	-0,9451	81	-1	-0,9769
FOXQ1	1167	-0,2743	7566	0,0326	2185	-0,2435	13187	1	0,0308	1131	-1	0,3069	1415	-1	0,2761
PAX7	879	-0,3252	28097	0,0000	1215	-0,3557	13231	1	-0,0305	1017	-1	0,3252	962	-1	0,3557
P4HA2	583	0,4033	3042	-0,0817	881	0,4324	13506	1	0,0291	468	-1	-0,4849	494	-1	-0,5140
RP11-103C16.2	3001	0,1532	25594	-0,0016	3329	0,1819	13548	1	0,0287	3483	-1	-0,1548	2539	-1	-0,1836
RP11-408N14.1	1588	0,2274	6054	-0,0443	2020	0,2560	13556	1	0,0287	1425	-1	-0,2717	1256	-1	-0,3004
ZDHHC17	830	0,3376	22271	-0,0028	1170	0,3656	13729	1	0,0280	931	-1	-0,3404	907	-1	-0,3684
CTD-2066L21.2	515	0,4263	13851	-0,0098	803	0,4542	13741	1	0,0279	590	-1	-0,4361	590	-1	-0,4640
SLC36A1	930	0,3134	8339	-0,0280	1725	0,2860	13864	1	-0,0274	924	-1	-0,3414	1165	-1	-0,3140
MAPKAPK3	1296	-0,2584	6141	0,0435	1728	-0,2858	13869	1	-0,0273	1155	-1	0,3020	1089	-1	0,3293
PIK3IP1	441	0,4639	8994	-0,0246	856	0,4396	14728	1	-0,0243	458	-1	-0,4885	589	-1	-0,4642
RP1-86D1.3	2468	0,1729	22372	-0,0024	2970	0,1966	15150	1	0,0237	2903	-1	-0,1753	2246	-1	-0,1990
GSR	948	-0,3083	7980	0,0300	1360	-0,3315	15397	1	-0,0232	947	-1	0,3384	937	-1	0,3615

CD59	434	0,4676	1000	-0,1488	843	0,4449	15557	1	-0,0226	263	-1	-0,6164	356	-1	-0,5937
RN7SKP97	6422	-0,0827	5954	0,0452	9376	-0,0610	15646	1	0,0218	4393	-1	0,1279	5097	-1	0,1061
CDC25B	411	-0,4762	1978	0,1066	800	-0,4547	15740	1	0,0215	301	-1	0,5829	416	-1	0,5614
CHST3	335	0,5235	7611	-0,0323	562	0,5449	15803	1	0,0213	346	-1	-0,5558	383	-1	-0,5772
TIMP2	35	1,0426	15360	-0,0078	89	1,0634	15937	1	0,0208	42	-1	-1,0504	58	-1	-1,0712
COL1A1	9	1,4803	8776	-0,0256	30	1,4605	16042	1	-0,0198	12	-1	-1,5060	14	-1	-1,4861
SGK2	277	-0,5627	8018	0,0298	567	-0,5440	16258	1	0,0187	285	-1	0,5925	388	-1	0,5738
MBP	358	-0,5131	10301	0,0192	594	-0,5307	17067	1	-0,0176	378	-1	0,5322	431	-1	0,5498
TAPT1	788	0,3443	2396	-0,0953	1188	0,3617	17142	1	0,0174	582	-1	-0,4396	614	-1	-0,4570
TMEM88	3161	0,1478	25273	-0,0020	4913	0,1313	17466	1	-0,0165	3620	-1	-0,1498	3872	-1	-0,1333
AHNAK	2292	-0,1811	93	0,3800	3785	-0,1647	17470	1	0,0164	336	-1	0,5611	438	-1	0,5446
ANKRD33B	1812	-0,2095	13295	0,0106	2436	-0,2258	17479	1	-0,0164	2041	-1	0,2201	1784	-1	0,2364
RP11-342M21.2	6810	-0,0777	8569	0,0268	6962	-0,0919	18752,5	1	-0,0142	5478	-1	0,1045	4469	-1	0,1186
TPST1	494	0,4375	6039	-0,0445	908	0,4242	18851	1	-0,0133	473	-1	-0,4821	576	-1	-0,4687
SLCO5A1	53	0,9178	7762	-0,0314	161	0,9050	18940	1	-0,0128	62	-1	-0,9492	95	-1	-0,9364
CRLF1	31	1,0792	11747	-0,0144	76	1,0916	19146	1	0,0124	34	-1	-1,0935	51	-1	-1,1059
RASGRP3	5	1,6240	5532	-0,0490	20	1,6351	20534	1	0,0111	6	-1	-1,6731	7	-1	-1,6841
TBC1D2	163	-0,6877	13647	0,0103	338	-0,6766	20538	1	0,0111	194	-1	0,6980	261	-1	0,6870
MED12L	1149	0,2765	9114	-0,0239	1712	0,2872	20939	1	0,0107	1166	-1	-0,3005	1187	-1	-0,3111
TESC	1636	-0,2234	3712	0,0707	2653	-0,2132	21090	1	0,0102	1216	-1	0,2941	1363	-1	0,2839
JUNB	140	0,7164	450	-0,2085	310	0,7065	21131	1	-0,0099	73	-1	-0,9249	104	-1	-0,9150
HIST1H2BI	707	-0,3634	1030	0,1470	1222	-0,3537	21200	1	0,0097	417	-1	0,5104	512	-1	0,5007
TGFBI	28	1,1101	3381	-0,0757	68	1,1198	21220	1	0,0096	26	-1	-1,1858	38	-1	-1,1954
BLOC1S2	85	0,8050	4598	-0,0586	222	0,7960	22538	1	-0,0090	93	-1	-0,8636	130	-1	-0,8546
PTPRK	71	0,8400	1906	-0,1091	192	0,8487	22953	1	0,0087	63	-1	-0,9491	86	-1	-0,9578
RP11-217E22.5	5906	0,0906	25057	-0,0020	6515	0,0990	23078	1	0,0085	6185,5	-1	-0,0926	5369,5	-1	-0,1011
SLAMF9	668	0,3758	19550	-0,0040	1158	0,3681	23381	1	-0,0077	759	-1	-0,3799	883	-1	-0,3722
CABLES1	957	-0,3068	1130	0,1417	1474	-0,3144	23398	1	-0,0076	550	-1	0,4485	618	-1	0,4561
CREM	96	0,7847	6240	-0,0427	239	0,7772	23425	1	-0,0075	108	-1	-0,8273	151	-1	-0,8199
NFATC2	638	0,3846	9831	-0,0208	1100	0,3777	24063	1	-0,0069	677	-1	-0,4054	775	-1	-0,3985
RP11-54F2.1	4602	-0,1119	11983	0,0137	6113	-0,1058	24633	1	0,0061	4494	-1	0,1256	4426,5	-1	0,1195
FLRT2	270	0,5696	225	-0,2725	495	0,5744	25115	1	0,0048	103	-1	-0,8421	134	-1	-0,8469
RP11-284F21.10	1164	-0,2746	1982	0,1065	1867	-0,2701	25169	1	0,0045	755	-1	0,3811	862	-1	0,3766

RXRA	1792	-0,2116	2099	0,1032	2744	-0,2086	25748	1	0,0030	1074	-1	0,3148	1179	-1	0,3118
ARHGAP26	354	-0,5141	3006	0,0823	625	-0,5170	25783	1	-0,0029	281	-1	0,5965	349	-1	0,5994
LRRC15	916	0,3171	17807	-0,0052	1452	0,3181	28510	1	0,0010	1029	-1	-0,3223	1111	-1	-0,3233
SERPINB9	1247	-0,2639	995	0,1491	1942	-0,2633	28625	1	0,0006	653	-1	0,4130	739	-1	0,4124
MTUS1	417	-0,4747	3	-1,0989	1	-2,2610	1	1	-1,7863	250	1	-0,6242	42	1	1,1621
IGFBP5	142	0,7127	6218	0,0428	2	2,1022	6	1	1,3895	215	1	-0,6699	1	1	-2,0594
TNS4	607	-0,3942	2	-1,1178	10	-1,7362	7	1	-1,3420	177	1	-0,7236	321	1	0,6184
KRT19	356	-0,5138	8	-0,8111	9	-1,7547	9	1	-1,2409	1193	1	-0,2973	91	1	0,9436
CTD-2377D24.6	12300	0,0325	1408	0,1278	63	1,1535	10	1	1,1210	6017	1	0,0953	66	1	-1,0257
ELF3	295	-0,5525	66	-0,4261	17	-1,6702	11	1	-1,1177	4456	1	0,1265	34	1	1,2441
SLFN11	1699	-0,2182	16	-0,6683	46	-1,2787	15	1	-1,0606	547	1	-0,4501	333	1	0,6104
GPRIN3	3437	-0,1398	20	-0,6080	60	-1,1738	17	1	-1,0339	504	1	-0,4682	404	1	0,5658
IFI44L	8843	0,0559	3129	0,0801	91	1,0595	20	1	1,0035	14060	1	0,0241	78	1	-0,9794
DHRS3	4674	-0,1107	71	-0,4124	75	-1,0989	21	1	-0,9882	1158	1	-0,3017	262	1	0,6865
VCAN	119	0,7459	6	0,9608	16	1,6952	22	1	0,9493	2132	1	0,2149	218	1	-0,7344
SH2B3	823	0,3393	25	0,5569	51	1,2653	24	1	0,9260	2084	1	0,2176	241	1	-0,7084
LAMA3	4163	-0,1215	639	-0,1814	98	-1,0406	25	1	-0,9192	8656	1	-0,0600	126	1	0,8592
EPHA5	4366	0,1169	132	0,3358	106	1,0245	27	1	0,9075	2062	1	0,2189	258	1	-0,6886
CXCL1	4939	0,1056	18	0,6348	114	1,0011	28	1	0,8955	382	1	0,5292	914	1	-0,3663
POF1B	9562	-0,0497	94	-0,3798	191	-0,8494	46	1	-0,7997	991	1	-0,3300	575	1	0,4696
ANTXR2	6571	0,0811	1375	0,1293	183	0,8620	49	1	0,7809	9914	1	0,0482	219	1	-0,7327
AKR1C2	54	-0,9153	67	-0,4240	14	-1,6960	50	1	-0,7807	454	1	0,4913	33	1	1,2720
SPINT2	2124	-0,1895	17	-0,6588	130	-0,9614	54	1	-0,7718	497	1	-0,4693	1237	1	0,3026
BCAT1	2224	0,1844	62	0,4439	135	0,9544	57	1	0,7699	1546	1	0,2595	497	1	-0,5104
DNER	13850	0,0259	609	0,1837	231	0,7858	58	1	0,7598	3395	1	0,1578	344	1	-0,6020
CFH	2450	-0,1733	134	-0,3353	149	-0,9250	59	1	-0,7516	3268	1	-0,1619	360	1	0,5897
SLC4A4	828	0,3381	260	0,2575	78	1,0896	60	1	0,7515	6942	1	-0,0806	143	1	-0,8321
HS3ST3B1	787	0,3444	2478	0,0934	80	1,0857	62	1	0,7413	1639	1	-0,2510	75	1	-0,9922
MCAM	6320	0,0843	49	0,4670	205	0,8246	63	1	0,7403	751	1	0,3827	954	1	-0,3576
PTGS2	597	-0,3968	23	-0,5675	70	-1,1147	67	1	-0,7179	3036	1	-0,1707	433	1	0,5472
LLGL2	710	-0,3623	51	-0,4644	85	-1,0757	69	1	-0,7134	5627	1	-0,1021	332	1	0,6113
GPX2	41	-0,9933	7	-0,8948	12	-1,7066	70	1	-0,7133	5818	1	0,0985	159	1	0,8118
EPS8	9234	-0,0524	205	-0,2803	261	-0,7551	75	1	-0,7027	1929	1	-0,2278	564	1	0,4749

AGR2	17	-1,2625	1	-1,2256	6	-1,9427	82	1	-0,6801	11407	1	0,0369	231	1	0,7170
SCARNA6	144	0,7119	7901	0,0305	11898	0,0395	83	1	-0,6725	209	1	-0,6815	21324	1	-0,0090
IL6	11099	0,0388	3293	0,0770	302	0,7096	84	1	0,6708	11215	1	0,0382	311	1	-0,6326
FSTL1	226	0,6159	97	0,3772	45	1,2814	89	1	0,6655	1783	1	-0,2387	107	1	-0,9042
PDK4	720	-0,3604	68	-0,4151	105	-1,0253	90	1	-0,6649	9160	1	-0,0547	335	1	0,6103
TBC1D8	504	-0,4319	166	-0,3093	77	-1,0903	93	1	-0,6584	4624	1	0,1226	177	1	0,7811
SLC27A2	184	-0,6632	79	-0,3970	42	-1,3151	96	1	-0,6519	1480	1	0,2662	101	1	0,9181
RP11-184M15.1	4407	0,1163	7944	0,0302	256	0,7607	99	1	0,6444	6600	1	-0,0861	221	1	-0,7305
PLA2G4A	1893	-0,2035	19	-0,6279	195	-0,8457	101	1	-0,6422	622	1	-0,4244	2007	1	0,2178
ADAMTS9	509	0,4299	1403	0,1279	92	1,0588	108	1	0,6289	1153	1	-0,3020	96	1	-0,9309
TNFSF9	3869	-0,1284	148	-0,3222	269	-0,7470	117	1	-0,6186	2517	1	-0,1938	700	1	0,4248
SREBF1	11138	-0,0386	103	-0,3728	365	-0,6548	118	1	-0,6161	969	1	-0,3341	1380	1	0,2820
S1PR5	309	0,5454	7421	0,0336	62	1,1609	119	1	0,6155	412	1	-0,5117	48	1	-1,1273
ADAMTS12	5977	-0,0896	180	-0,2999	313	-0,7011	121	1	-0,6115	2221	1	-0,2103	770	1	0,4012
PBX3	938	0,3110	1325	0,1312	153	0,9221	122	1	0,6111	2812	1	-0,1798	172	1	-0,7909
SHROOM3	438	-0,4663	140	-0,3332	86	-1,0757	124	1	-0,6094	4205	1	0,1331	212	1	0,7425
HIST1H3I	11132	-0,0387	42	-0,4830	392	-0,6370	128	1	-0,5983	564	1	-0,4443	3195	1	0,1540
PCLO	10470	-0,0432	173	-0,3043	391	-0,6373	129	1	-0,5940	1530	1	-0,2611	1066	1	0,3330
IL18	1343	-0,2536	844	-0,1609	194	-0,8469	130	1	-0,5934	6187	1	0,0926	263	1	0,6860
GALNT9	11061	0,0390	2005	0,1056	400	0,6299	132	1	0,5909	8063	1	0,0666	473	1	-0,5243
SHC3	4079	0,1236	1412	0,1277	299	0,7117	135	1	0,5881	25880	1	0,0041	367	1	-0,5840
CD70	4292	-0,1186	117	-0,3545	311	-0,7062	136	1	-0,5876	1811	1	-0,2359	976	1	0,3517
NRIP3	1215	0,2680	5832	0,0463	189	0,8531	137	1	0,5851	2017	1	-0,2217	162	1	-0,8068
ALDH3A1	36	-1,0388	89	-0,3835	21	-1,6216	139	1	-0,5829	225	1	0,6553	36	1	1,2382
AC006262.5	4153	-0,1218	34	-0,5006	319	-0,6952	143	1	-0,5735	764	1	-0,3789	2324	1	0,1946
NKX3-1	753	0,3526	2328	0,0971	156	0,9161	150	1	0,5635	1593	1	-0,2555	152	1	-0,8190
CORO2B	79	0,8158	7334	0,0342	37	1,3770	152	1	0,5612	138	1	-0,7816	26	1	-1,3428
SYT12	14452	-0,0229	11440	-0,0152	483	-0,5797	154	1	-0,5568	22071	1	0,0077	406	1	0,5646
KRT8	1680	-0,2197	41	-0,4846	241	-0,7762	155	1	-0,5565	1496	1	-0,2649	1307	1	0,2916
CNN2	5829	0,0916	36	0,4917	378	0,6433	157	1	0,5517	695	1	0,4001	3274	1	-0,1516
TRIM8	2094	0,1910	293	0,2461	274	0,7412	158	1	0,5502	9118	1	0,0551	523	1	-0,4951
LRRK2	4955	-0,1054	169	-0,3084	364	-0,6550	160	1	-0,5496	2344	1	-0,2030	1001	1	0,3466
ZNF724P	14536	0,0226	219	0,2736	500	0,5716	161	1	0,5490	1641	1	0,2509	1271	1	-0,2981

GPRC5C	796	-0,3437	3718	-0,0706	169	-0,8892	163	1	-0,5455	1413	1	0,2731	154	1	0,8186
COL27A1	398	0,4823	1954	0,1076	104	1,0271	166	1	0,5447	781	1	-0,3748	99	1	-0,9195
NLRC5	7891	0,0654	491	0,2017	429	0,6097	167	1	0,5442	4089	1	0,1362	754	1	-0,4080
TPD52L1	883	-0,3245	182	-0,2992	180	-0,8685	168	1	-0,5440	13830	1	0,0253	399	1	0,5693
KIRREL3	881	0,3246	418	0,2145	181	0,8676	169	1	0,5430	5212	1	-0,1101	295	1	-0,6531
DYSF	49	0,9546	9779	0,0211	958	0,4136	170	1	-0,5410	67	1	-0,9335	795	1	-0,3925
SLFN13	4637	-0,1113	249	-0,2609	373	-0,6499	172	1	-0,5386	3628	1	-0,1496	806	1	0,3890
FGF5	18950	0,0123	13169	0,0109	557	0,5470	173	1	0,5347	28656,5	1	-0,0014	454	1	-0,5361
TAGLN	24	1,1659	167	0,3092	13	1,6976	174	1	0,5317	98	1	-0,8567	20	1	-1,3884
KRT18	968	-0,3051	110	-0,3698	197	-0,8364	175	1	-0,5313	8224	1	-0,0647	583	1	0,4666
MYO15B	1445	-0,2435	500	-0,2000	245	-0,7728	179	1	-0,5293	10470	1	0,0435	391	1	0,5728
SORCS2	205	0,6382	1083	0,1441	61	1,1658	180	1	0,5276	446	1	-0,4941	67	1	-1,0217
TSPAN2	56	0,9004	6793	0,0382	33	1,4260	181	1	0,5256	94	1	-0,8622	21	1	-1,3877
PTPN14	9823	0,0478	76	0,4073	505	0,5696	184	1	0,5219	841	1	0,3596	2986	1	-0,1623
LTBP4	126	0,7311	242	0,2642	55	1,2528	185	1	0,5217	512	1	-0,4668	76	1	-0,9886
RASGRP1	589	0,4006	3001	0,0824	152	0,9221	186	1	0,5215	1052	1	-0,3182	140	1	-0,8397
TNS3	221	-0,6224	1711	-0,1152	64	-1,1438	187	1	-0,5215	426	1	0,5072	65	1	1,0287
PDCD1LG2	2873	0,1571	692	0,1751	343	0,6727	189	1	0,5156	16124	1	0,0180	519	1	-0,4977
PLCB4	419	0,4739	1356	0,1301	119	0,9867	191	1	0,5128	917	1	-0,3438	128	1	-0,8566
LGSN	11449	-0,0370	1072	-0,1446	554	-0,5484	192	1	-0,5114	5332	1	-0,1077	761	1	0,4037
RP11-166D19.1	1911	0,2021	420	0,2143	298	0,7119	194	1	0,5098	18637	1	0,0122	520	1	-0,4976
GALNT10	11381	0,0376	912	0,1555	560	0,5465	195	1	0,5089	4833	1	0,1180	801	1	-0,3909
KIAA1024	887	0,3230	12510	0,0125	200	0,8312	196	1	0,5082	1109	1	-0,3106	153	1	-0,8187
DAPK1	114	-0,7496	178	-0,3027	53	-1,2568	198	1	-0,5072	556	1	0,4469	89	1	0,9541
PCYT2	5862	-0,0912	102	-0,3728	445	-0,5982	199	1	-0,5070	1333	1	-0,2816	1915	1	0,2254
CHRNA3	1288	0,2591	28055	0,0001	248	0,7657	201	1	0,5066	1551	1	-0,2589	185	1	-0,7656
CYP2S1	743	-0,3555	1086	-0,1441	184	-0,8619	202	1	-0,5064	2201	1	0,2115	229	1	0,7179
ZYX	576	0,4055	109	0,3707	159	0,9075	203	1	0,5019	11792	1	-0,0348	451	1	-0,5367
ST3GAL1	866	0,3276	7194	0,0351	203	0,8283	204	1	0,5007	1229	1	-0,2925	170	1	-0,7932
FMNL3	1572	0,2305	1216	0,1372	281	0,7286	207	1	0,4981	6146	1	-0,0933	357	1	-0,5914
LPXN	28267	0,0002	6712	0,0390	677	0,4958	210	1	0,4956	11141	1	0,0388	617	1	-0,4568
ITPRIP	449	0,4591	1831	0,1110	138	0,9505	215	1	0,4914	896	1	-0,3481	141	1	-0,8395
MAP7D2	1383	-0,2494	162	-0,3138	275	-0,7398	216	1	-0,4904	8244	1	-0,0644	697	1	0,4260

AKR1C3	257	-0,5821	308	-0,2418	88	-1,0698	219	1	-0,4876	932	1	0,3403	146	1	0,8280
KCNS3	5798	-0,0921	149	-0,3209	492	-0,5754	222	1	-0,4834	1914	1	-0,2289	1597	1	0,2545
ARSE	4494	-0,1144	1385	-0,1289	449	-0,5973	223	1	-0,4829	17401	1	-0,0145	577	1	0,4684
KIAA1644	10122	0,0456	218	0,2750	601	0,5268	229	1	0,4811	1904	1	0,2294	1618	1	-0,2518
GPRC5A	856	-0,3304	37	-0,4908	213	-0,8104	231	1	-0,4800	3315	1	-0,1605	1136	1	0,3196
F3	1154	0,2761	3024	0,0820	265	0,7533	236	1	0,4771	2507	1	-0,1941	277	1	-0,6713
DMKN	1397	-0,2484	281	-0,2505	287	-0,7232	238	1	-0,4748	28479	1	-0,0021	569	1	0,4727
SYT13	44	0,9863	60	0,4478	31	1,4595	240	1	0,4732	371	1	-0,5385	69	1	-1,0117
GNPNAT1	1689	0,2192	3470	0,0744	323	0,6916	242	1	0,4724	3799	1	-0,1448	323	1	-0,6172
NTN4	225	-0,6176	712	-0,1730	79	-1,0880	244	1	-0,4704	562	1	0,4446	105	1	0,9150
ABLIM1	506	-0,4310	584	-0,1875	162	-0,8995	247	1	-0,4684	1722	1	0,2436	237	1	0,7120
ANXA4	2260	-0,1824	484	-0,2032	371	-0,6503	248	1	-0,4679	15032	1	-0,0207	640	1	0,4471
RASA3	3253	0,1449	758	0,1677	424	0,6117	249	1	0,4668	14416	1	0,0228	651	1	-0,4440
ARHGAP29	294	-0,5527	354	-0,2273	110	-1,0145	254	1	-0,4617	1016	1	0,3254	175	1	0,7872
BVES	364	0,5094	607	0,1839	124	0,9711	255	1	0,4617	1015	1	-0,3255	174	1	-0,7872
KIF26B	1073	0,2895	22083	0,0030	267	0,7509	256	1	0,4614	1289	1	-0,2866	203	1	-0,7480
VASN	505	0,4316	26582	0,0009	166	0,8930	257	1	0,4613	605	1	-0,4307	111	1	-0,8921
ST3GAL5	346	-0,5185	481	-0,2036	121	-0,9781	258	1	-0,4596	1073	1	0,3149	182	1	0,7745
GLRX	2819	-0,1594	482	-0,2034	417	-0,6190	259	1	-0,4596	10422	1	-0,0440	728	1	0,4156
ATP1B1	12837	-0,0294	478	-0,2038	699	-0,4885	260	1	-0,4590	2928	1	-0,1744	1354	1	0,2847
DCDC2	521	-0,4245	86	-0,3889	173	-0,8829	261	1	-0,4584	11634	1	0,0356	525	1	0,4940
MITF	322	-0,5305	547	-0,1930	118	-0,9868	262	1	-0,4564	951	1	0,3375	167	1	0,7939
ARHGAP31	199	0,6468	365	0,2244	73	1,1010	265	1	0,4542	628	1	-0,4224	118	1	-0,8766
SSFA2	15002	-0,0216	136	-0,3343	740	-0,4744	268	1	-0,4528	1091	1	-0,3128	3626	1	0,1401
TOB1	215	-0,6279	80	-0,3961	83	-1,0784	272	1	-0,4505	1870	1	0,2318	267	1	0,6822
MYOM3	251	0,5850	192	0,2919	103	1,0289	279	1	0,4438	1222	1	-0,2932	216	1	-0,7370
ABCA3	4460	0,1153	141	0,3292	520	0,5591	280	1	0,4438	2153	1	0,2139	1869	1	-0,2299
CYR61	850	0,3323	154	0,3188	243	0,7756	282	1	0,4433	17869	1	-0,0135	616	1	-0,4568
SLC16A3	1145	0,2768	172	0,3052	293	0,7173	287	1	0,4405	13027	1	0,0283	741	1	-0,4121
JUN	804	0,3428	808	0,1636	237	0,7804	289	1	0,4375	2824	1	-0,1792	324	1	-0,6168
ARNTL2	2886	0,1567	278	0,2516	458	0,5937	290	1	0,4370	6033	1	0,0950	1026	1	-0,3420
POPDC3	2122	0,1896	1442	0,1265	405	0,6266	291	1	0,4369	8361	1	-0,0632	514	1	-0,5001
FAM46A	2862	-0,1575	515	-0,1978	457	-0,5942	292	1	-0,4366	10884	1	-0,0403	786	1	0,3963

LAMC2	6	1,5784	5168	0,0525	5	2,0137	294	1	0,4353	10	1	-1,5259	3	1	-1,9613
LRRC20	1658	-0,2214	1620	-0,1182	370	-0,6506	306	1	-0,4292	5563	1	0,1031	464	1	0,5324
CD9	6900	-0,0765	2425	-0,0948	653	-0,5043	309	1	-0,4277	16052	1	-0,0183	748	1	0,4095
GALNT5	3142	-0,1486	495	-0,2013	493	-0,5751	310	1	-0,4264	9391	1	-0,0526	877	1	0,3738
PDLIM7	375	0,4993	87	0,3870	148	0,9251	312	1	0,4258	5113	1	-0,1123	447	1	-0,5381
FHL3	2638	0,1665	2910	0,0842	461	0,5918	315	1	0,4253	6829	1	-0,0823	500	1	-0,5076
IGFBP3	188	0,6603	248	0,2613	81	1,0846	318	1	0,4243	701	1	-0,3991	149	1	-0,8234
SERPINB1	1244	-0,2644	655	-0,1791	330	-0,6866	322	1	-0,4222	6648	1	0,0852	501	1	0,5074
PABPC4L	676	0,3740	6651	0,0395	223	0,7958	323	1	0,4219	965	1	-0,3345	194	1	-0,7564
BMP2	7708	0,0675	12263	0,0131	697	0,4888	325	1	0,4213	9182,5	1	-0,0545	562	1	-0,4757
RAET1G	5874	0,0911	20127	0,0036	644	0,5103	328	1	0,4193	6499	1	-0,0875	503	1	-0,5068
DOCK2	67	0,8444	114	0,3597	52	1,2618	332	1	0,4174	470	1	-0,4847	108	1	-0,9021
CNNM1	486	-0,4394	156	-0,3167	187	-0,8563	334	1	-0,4169	4618	1	0,1227	446	1	0,5396
NCALD	9126	-0,0535	1530	-0,1224	752	-0,4700	335	1	-0,4166	7842	1	-0,0689	996	1	0,3476
ARHGEF40	135	0,7187	247	0,2614	66	1,1350	336	1	0,4163	533	1	-0,4574	119	1	-0,8737
FAT3	22339	-0,0074	360	-0,2264	913	-0,4224	337	1	-0,4150	2059	1	-0,2190	2299	1	0,1960
SLC29A1	233	0,6080	849	0,1605	107	1,0228	338	1	0,4149	553	1	-0,4475	123	1	-0,8623
B4GALT4	1273	-0,2610	85	-0,3900	340	-0,6739	340	1	-0,4128	4354	1	-0,1290	1364	1	0,2839
MMP2	4	1,6519	613	0,1836	4	2,0629	344	1	0,4110	15	1	-1,4682	4	1	-1,8792
LPP	20100	0,0098	77	0,4044	919	0,4202	346	1	0,4104	720	1	0,3946	16877	1	-0,0159
GLDC	14551	-0,0225	433	-0,2113	882	-0,4321	348	1	-0,4096	2608	1	-0,1888	1971	1	0,2208
S100A6	11380	-0,0376	316	-0,2399	836	-0,4467	349	1	-0,4092	2350	1	-0,2024	2125	1	0,2068
AUTS2	2809	0,1598	1938	0,1080	511	0,5675	353	1	0,4077	9489	1	-0,0518	603	1	-0,4595
FAM129A	22359	0,0073	376	0,2222	959	0,4134	354	1	0,4061	2129	1	0,2150	2394	1	-0,1911
ABCC3	1098	-0,2847	2222	-0,0998	325	-0,6881	364	1	-0,4034	2690	1	0,1849	363	1	0,5883
DUSP7	2118	0,1897	1148	0,1409	465	0,5905	369	1	0,4008	9838	1	-0,0488	632	1	-0,4496
ACO1	11064	0,0390	48	0,4717	860	0,4379	370	1	0,3989	600	1	0,4327	12001	1	0,0338
PTGES	991	-0,3019	430	-0,2126	317	-0,6973	376	1	-0,3955	6386	1	0,0892	552	1	0,4847
MMP7	5909	-0,0906	854	-0,1603	710	-0,4854	378	1	-0,3949	7781	1	-0,0697	1104	1	0,3251
LIPH	3084	-0,1504	1425	-0,1272	566	-0,5444	380	1	-0,3939	14305	1	0,0233	724	1	0,4172
PACSIN3	678	0,3729	2585	0,0908	254	0,7636	386	1	0,3907	1329	1	-0,2821	274	1	-0,6728
RGMB	8764	0,0566	2047	0,1046	835	0,4472	388	1	0,3906	9939	1	0,0479	1023	1	-0,3426
GXYLT2	235	0,6064	1980	0,1065	115	0,9968	389	1	0,3903	435	1	-0,4999	113	1	-0,8903

TSHZ3	489	0,4386	1986	0,1063	202	0,8287	390	1	0,3901	984	1	-0,3323	224	1	-0,7224
KLHL21	3985	0,1254	2052	0,1044	632	0,5152	391	1	0,3898	14898	1	-0,0210	745	1	-0,4108
KIAA1549L	112	0,7503	3051	0,0815	65	1,1391	393	1	0,3888	216	1	-0,6689	61	1	-1,0576
SAT2	9101	-0,0538	525	-0,1962	852	-0,4409	399	1	-0,3871	3872	1	-0,1424	1691	1	0,2447
PURB	14365	0,0235	724	0,1712	978	0,4100	402	1	0,3866	3692	1	0,1477	1758	1	-0,2388
TNS1	3	1,6781	2519	0,0925	3	2,0641	403	1	0,3860	9	1	-1,5856	2	1	-1,9716
COL4A1	12	1,3870	61	0,4466	8	1,7726	405	1	0,3855	66	1	-0,9405	29	1	-1,3260
IL7R	138	0,7170	603	0,1852	74	1,1009	410	1	0,3839	379	1	-0,5317	103	1	-0,9156
LFNG	310	0,5435	4201	0,0634	147	0,9272	412	1	0,3837	478	1	-0,4801	122	1	-0,8637
TMC7	6088	-0,0877	596	-0,1857	753	-0,4700	415	1	-0,3824	5829	1	-0,0981	1359	1	0,2843
FLNA	1269	0,2614	88	0,3836	380	0,6429	420	1	0,3815	4650	1	0,1222	1560	1	-0,2593
MGLL	12140	-0,0333	466	-0,2055	971	-0,4114	426	1	-0,3781	2988	1	-0,1722	2145	1	0,2059
POMT1	7027	-0,0749	600	-0,1854	821	-0,4501	435	1	-0,3752	5194	1	-0,1105	1517	1	0,2647
TRHDE	21928	0,0093	2981	0,0829	1070	0,3838	439	1	0,3745	7456	1	0,0736	1252	1	-0,3010
SERPINH1	15813	0,0196	159	0,3154	1036	0,3939	441	1	0,3743	1206	1	0,2959	6845	1	-0,0785
C11orf86	715	-0,3615	793	-0,1647	277	-0,7354	442	1	-0,3739	2449	1	0,1968	396	1	0,5706
RP11-438N16.1	26071	-0,0039	240	-0,2649	1107	-0,3762	445	1	-0,3723	1532	1	-0,2609	4829	1	0,1114
TOM1L2	8523	-0,0587	575	-0,1889	894	-0,4298	450	1	-0,3711	4311	1	-0,1302	1730	1	0,2409
PRR15	2328	-0,1791	284	-0,2478	548	-0,5498	452	1	-0,3707	7870	1	-0,0687	1241	1	0,3020
GRB7	2133	-0,1892	502	-0,1999	519	-0,5591	455	1	-0,3699	19150	1	-0,0106	946	1	0,3593
LURAP1L	3753	-0,1316	9250	-0,0232	661	-0,5004	459	1	-0,3689	5298	1	0,1083	559	1	0,4772
CDK6	677	0,3738	214	0,2756	273	0,7422	460	1	0,3685	5826	1	-0,0981	584	1	-0,4666
ST8SIA4	452	-0,4567	69	-0,4150	206	-0,8240	465	1	-0,3673	10710	1	0,0417	750	1	0,4090
RAB31	8778	-0,0565	938	-0,1534	916	-0,4220	469	1	-0,3655	5902	1	-0,0969	1477	1	0,2686
GLI3	25514	0,0049	341	0,2315	1150	0,3696	473	1	0,3647	1949	1	0,2266	3708	1	-0,1381
FRMD6	21	1,2038	1939	0,1080	26	1,5683	475	1	0,3645	33	1	-1,0958	16	1	-1,4603
SPATA20	1004	-0,2992	511	-0,1989	354	-0,6626	476	1	-0,3634	5701	1	0,1004	592	1	0,4638
ST6GAL2	14557	-0,0224	38	-0,4908	1072	-0,3832	487	1	-0,3608	503	1	-0,4684	5017	1	-0,1077
LACTB2	2813	-0,1596	2606	-0,0903	622	-0,5186	490	1	-0,3590	7818	1	0,0693	693	1	0,4283
PIEZO1	536	0,4188	381	0,2214	240	0,7770	492	1	0,3582	2435	1	-0,1975	422	1	-0,5557
VAV3	1507	-0,2370	334	-0,2352	456	-0,5944	494	1	-0,3574	28539	1	0,0018	947	1	0,3592
ABCA12	6568	-0,0811	681	-0,1761	861	-0,4377	498	1	-0,3565	6032	1	-0,0950	1542	1	0,2615
TSPAN13	1647	-0,2221	256	-0,2588	486	-0,5780	502	1	-0,3559	11432	1	-0,0368	1138	1	0,3191

MYO5C	440	-0,4640	1698	-0,1157	208	-0,8187	507	1	-0,3548	895	1	0,3483	248	1	0,7031
GAS6	1476	0,2397	2414	0,0951	459	0,5936	510	1	0,3538	3800	1	-0,1447	517	1	-0,4985
GULP1	1376	-0,2503	2410	-0,0951	437	-0,6023	513	1	-0,3520	3476	1	0,1552	502	1	0,5072
MARC1	2106	-0,1904	512	-0,1986	571	-0,5424	514	1	-0,3520	21951	1	-0,0083	1016	1	0,3438
TUFT1	502	0,4324	8379	0,0278	233	0,7837	518	1	0,3514	681	1	-0,4046	195	1	-0,7560
ALPK2	547	0,4147	57	0,4513	250	0,7656	521	1	0,3509	11452	1	0,0366	1164	1	-0,3143
KLHL29	734	0,3579	1887	0,1096	307	0,7086	524	1	0,3507	1664	1	-0,2484	350	1	-0,5990
ECH1	1926	-0,2014	1116	-0,1424	543	-0,5517	527	1	-0,3504	8751	1	0,0590	749	1	0,4093
ALCAM	3274	0,1441	198	0,2853	688	0,4922	530	1	0,3481	3922	1	0,1412	2124	1	-0,2069
HSD3B7	5101	-0,1027	2312	-0,0975	826	-0,4491	536	1	-0,3464	24164	1	0,0052	977	1	0,3516
NXPE3	1874	0,2050	81	0,3953	544	0,5513	537	1	0,3463	2583	1	0,1903	3137	1	-0,1560
PIK3CD	206	0,6382	8693	0,0261	120	0,9837	540	1	0,3455	264	1	-0,6121	88	1	-0,9575
NXPH4	237	0,6036	3108	0,0805	139	0,9479	542	1	0,3443	394	1	-0,5231	121	1	-0,8674
PLS1	241	-0,5953	340	-0,2319	143	-0,9389	547	1	-0,3437	825	1	0,3633	246	1	0,7070
COL6A3	3770	0,1311	19209	0,0044	746	0,4722	554	1	0,3411	4448	1	-0,1267	581	1	-0,4679
ADAM19	18	1,2528	486	0,2031	24	1,5920	558	1	0,3392	43	1	-1,0497	19	1	-1,3889
SGK223	306	-0,5479	2856	-0,0850	170	-0,8870	560	1	-0,3391	523	1	0,4629	164	1	0,8020
TNFSF15	622	-0,3888	158	-0,3157	282	-0,7277	561	1	-0,3389	7477	1	0,0731	743	1	0,4120
SLC7A1	742	0,3557	1230	0,1362	321	0,6945	562	1	0,3388	2052	1	-0,2195	419	1	-0,5583
PDE8B	366	-0,5086	1872	-0,1099	193	-0,8473	564	1	-0,3387	705	1	0,3987	214	1	0,7374
PC	2315	-0,1796	644	-0,1806	624	-0,5174	567	1	-0,3379	29078	1	-0,0010	1054	1	0,3369
ITGA5	168	0,6843	1327	0,1311	108	1,0218	568	1	0,3374	350	1	-0,5532	112	1	-0,8907
MKNK2	5744	-0,0928	3849	-0,0686	893	-0,4299	570	1	-0,3371	14055	1	0,0242	939	1	0,3613
MLLT11	889	0,3227	645	0,1804	359	0,6590	575	1	0,3363	3882	1	-0,1423	558	1	-0,4786
EXT1	5253	0,1005	401	0,2175	862	0,4363	576	1	0,3357	4880	1	0,1169	1993	1	-0,2188
C12orf80	21981	-0,0093	1146	-0,1410	1283	-0,3429	581	1	-0,3337	4241	1	-0,1317	2210	1	0,2019
HSPA2	3913	-0,1272	179	-0,3026	782	-0,4592	589	1	-0,3320	2899	1	-0,1754	3126	1	0,1566
GRAMD1A	5221	-0,1011	73	-0,4106	878	-0,4329	590	1	-0,3318	1118	1	-0,3096	14724	1	0,0223
S100A4	3144	-0,1485	694	-0,1750	730	-0,4803	591	1	-0,3317	13514	1	-0,0265	1222	1	0,3052
IQGAP2	1063	-0,2905	465	-0,2055	414	-0,6210	593	1	-0,3305	6667	1	0,0850	729	1	0,4154
CREB5	25486	0,0049	1011	0,1481	1326	0,3350	596	1	0,3301	3842	1	0,1432	2468	1	-0,1869
ISYNA1	4417	-0,1161	669	-0,1774	844	-0,4448	603	1	-0,3288	8535	1	-0,0613	1493	1	0,2675
PMP22	443	-0,4633	298	-0,2451	226	-0,7919	604	1	-0,3286	2068	1	0,2182	434	1	0,5468

FOSL2	7747	0,0671	82	0,3936	1031	0,3955	606	1	0,3283	1012	1	0,3264	28422	1	-0,0019
PRNP	1138	0,2781	518	0,1974	435	0,6059	607	1	0,3278	6940	1	-0,0807	752	1	-0,4084
MAP2K6	2919	-0,1557	1138	-0,1414	724	-0,4821	610	1	-0,3264	17629	1	0,0143	1033	1	0,3407
LIMA1	1427	0,2453	22383	0,0024	501	0,5712	612	1	0,3259	1730	1	-0,2429	400	1	-0,5689
CDC42EP2	670	0,3755	3843	0,0688	312	0,7011	614	1	0,3256	1134	1	-0,3067	312	1	-0,6323
RP11-244F12.3	488	0,4389	1662	0,1170	252	0,7645	615	1	0,3256	1031	1	-0,3219	300	1	-0,6475
RP11-688I9.2	4753	0,1089	5483	0,0495	871	0,4345	616	1	0,3256	8712	1	-0,0594	827	1	-0,3850
ARRB2	931	-0,3133	211	-0,2776	390	-0,6379	620	1	-0,3246	11626	1	0,0357	943	1	0,3603
BCOR	1282	0,2595	1398	0,1281	478	0,5840	621	1	0,3246	4253	1	-0,1314	619	1	-0,4560
ZIC2	732	0,3584	926	0,1546	334	0,6827	623	1	0,3243	2332	1	-0,2039	466	1	-0,5281
TRIM36	4608	0,1118	1806	0,1119	864	0,4359	624	1	0,3241	29639	1	0,0001	1106	1	-0,3240
RUNX1	228	0,6133	244	0,2634	144	0,9364	627	1	0,3231	887	1	-0,3499	273	1	-0,6730
FERMT1	2039	0,1934	2685	0,0884	631	0,5156	634	1	0,3222	5453	1	-0,1050	694	1	-0,4272
EFNA1	1176	0,2733	4692	0,0576	453	0,5954	636	1	0,3221	2115	1	-0,2157	449	1	-0,5378
C10orf54	154	-0,6986	853	-0,1604	109	-1,0206	637	1	-0,3219	372	1	0,5382	125	1	0,8601
B9D1	1942	-0,2003	1018	-0,1478	611	-0,5217	640	1	-0,3214	9414	1	0,0524	876	1	0,3739
MIEN1	11688	-0,0362	1952	-0,1076	1206	-0,3571	644	1	-0,3209	7632	1	-0,0714	1646	1	0,2494
PTPN6	5513	-0,0965	1060	-0,1453	941	-0,4168	647	1	-0,3204	9837	1	-0,0488	1450	1	0,2715
IQSEC2	3555	-0,1366	4595	-0,0587	792	-0,4566	649	1	-0,3200	7131	1	0,0779	780	1	0,3979
TRIML2	4523	-0,1137	2115	-0,1028	873	-0,4337	650	1	-0,3199	19095	1	0,0109	1078	1	0,3309
LZTS2	6598	-0,0807	923	-0,1548	1009	-0,4006	651	1	-0,3199	7419	1	-0,0741	1675	1	0,2458
LRP12	751	0,3530	2226	0,0997	345	0,6715	660	1	0,3186	1616	1	-0,2533	394	1	-0,5719
IL11	7	1,4991	39	0,4887	7	1,8171	663	1	0,3180	50	1	-1,0104	28	1	-1,3284
PSMD2	287	0,5590	5874	0,0459	177	0,8754	674	1	0,3163	409	1	-0,5131	145	1	-0,8295
HS3ST3A1	15058	0,0213	11824	0,0141	1320	0,3361	681	1	0,3148	22715,5	1	-0,0071	1118	1	-0,3220
SDC1	582	0,4037	9965	0,0203	291	0,7180	683	1	0,3142	749	1	-0,3834	252	1	-0,6977
RAB27B	391	-0,4866	25337	-0,0019	220	-0,8006	684	1	-0,3140	469	1	0,4847	166	1	0,7988
RAB3B	2420	0,1748	404	0,2171	698	0,4888	685	1	0,3139	10625	1	0,0423	1448	1	-0,2717
PDGFC	230	0,6088	3578	0,0726	154	0,9221	688	1	0,3132	374	1	-0,5363	132	1	-0,8495
FOXP1	39	1,0066	378	0,2218	41	1,3184	696	1	0,3118	135	1	-0,7848	54	1	-1,0966
INHBA	89	0,7975	17259	0,0056	72	1,1089	699	1	0,3114	129	1	-0,7919	52	1	-1,1033
CDYL2	3478	0,1386	10967	0,0167	824	0,4496	702	1	0,3109	4662	1	-0,1219	680	1	-0,4328
TPBG	3635	-0,1344	75	-0,4075	845	-0,4446	706	1	-0,3102	1414	1	-0,2730	11383	1	0,0372

NT5E	101	0,7699	867	0,1596	82	1,0787	713	1	0,3088	267	1	-0,6103	100	1	-0,9191
ITPR2	934	0,3123	4892	0,0555	415	0,6204	715	1	0,3081	1578	1	-0,2568	405	1	-0,5650
LPCAT1	3424	-0,1403	11244	-0,0157	829	-0,4480	719	1	-0,3077	4538	1	0,1246	682	1	0,4323
MARCH4	43	0,9864	3821	0,0692	43	1,2939	721	1	0,3075	75	1	-0,9172	37	1	-1,2248
KIF13B	1094	-0,2857	78	-0,4030	462	-0,5908	736	1	-0,3051	4860	1	-0,1173	2453	1	0,1878
ROCK1	6503	0,0819	277	0,2517	1059	0,3866	737	1	0,3047	3065	1	0,1698	3816	1	-0,1348
AKR1B10	33	-1,0575	4920	-0,0551	39	-1,3606	744	1	-0,3030	51	1	1,0024	30	1	1,3054
OAS1	1971	-0,1978	741	-0,1696	662	-0,5004	746	1	-0,3026	13041	1	0,0282	1079	1	0,3309
ETS2	571	0,4076	195	0,2907	304	0,7095	751	1	0,3018	4878	1	-0,1170	722	1	-0,4188
MYO18A	2117	-0,1898	494	-0,2015	689	-0,4916	752	1	-0,3018	18822	1	-0,0118	1314	1	0,2901
ENTPD7	4824	0,1075	7749	0,0315	986	0,4085	755	1	0,3011	7257	1	-0,0760	858	1	-0,3771
ACBD5	4207	-0,1204	744	-0,1694	918	-0,4213	756	1	-0,3010	9806	1	-0,0491	1617	1	0,2519
SLC45A3	864	0,3287	8208	0,0288	401	0,6295	759	1	0,3008	1170	1	-0,2999	346	1	-0,6007
PERP	4577	-0,1125	1087	-0,1440	961	-0,4132	760	1	-0,3007	12405	1	-0,0315	1472	1	0,2692
CACNG6	2010	-0,1949	352	-0,2277	680	-0,4952	762	1	-0,3003	12155	1	-0,0328	1492	1	0,2675
TRIM6	882	-0,3246	5390	-0,0504	410	-0,6247	764	1	-0,3001	1402	1	0,2742	387	1	0,5743
PAWR	1335	0,2543	1495	0,1240	535	0,5543	766	1	0,3000	4307	1	-0,1303	690	1	-0,4303
SOX8	3776	0,1311	14730	0,0084	889	0,4308	767	1	0,2998	4623	1	-0,1226	714	1	-0,4224
ADRA1B	1580	-0,2287	2198	-0,1006	599	-0,5276	774	1	-0,2989	4386	1	0,1281	695	1	0,4270
RP11-688I9.4	12657	0,0304	6691	0,0391	1389	0,3272	782	1	0,2967	21684	1	0,0087	1326	1	-0,2881
MSN	819	0,3401	1569	0,1206	394	0,6364	788	1	0,2963	2051	1	-0,2196	493	1	-0,5159
ALOX12P2	1416	-0,2466	863	-0,1598	575	-0,5410	798	1	-0,2944	6546	1	0,0868	842	1	0,3812
UBB	8574	-0,0583	1481	-0,1247	1226	-0,3526	799	1	-0,2944	8084	1	-0,0664	1890	1	0,2280
TMC5	340	-0,5217	1949	-0,1077	210	-0,8157	801	1	-0,2940	648	1	0,4141	243	1	0,7080
UCP2	6120	-0,0872	4041	-0,0657	1088	-0,3802	810	1	-0,2930	14784	1	0,0216	1160	1	0,3146
HSPB8	766	0,3495	1448	0,1261	382	0,6424	811	1	0,2929	1990	1	-0,2234	490	1	-0,5163
ANPEP	196	0,6516	855	0,1603	140	0,9444	812	1	0,2928	453	1	-0,4914	176	1	-0,7841
COL4A2	45	0,9815	188	0,2956	48	1,2739	816	1	0,2925	203	1	-0,6858	80	1	-0,9783
DLG3	1560	-0,2317	233	-0,2688	614	-0,5212	836	1	-0,2895	11375	1	-0,0372	1613	1	0,2524
LPAR1	380	-0,4938	1658	-0,1170	235	-0,7830	839	1	-0,2892	773	1	0,3768	280	1	0,6660
SCRN2	9805	-0,0480	422	-0,2138	1313	-0,3371	842	1	-0,2891	3168	1	-0,1658	4253	1	0,1233
CALD1	481	0,4424	3194	0,0788	279	0,7315	843	1	0,2891	824	1	-0,3635	296	1	-0,6527
HRH1	960	0,3066	4497	0,0599	455	0,5950	847	1	0,2884	1684	1	-0,2467	455	1	-0,5351

LSMD1	1209	-0,2687	467	-0,2055	529	-0,5567	855	1	-0,2880	8352	1	0,0632	980	1	0,3512
SALL4	6502	-0,0819	818	-0,1629	1149	-0,3697	856	1	-0,2877	6923	1	-0,0809	2126	1	0,2068
COL21A1	1289	-0,2590	453	-0,2074	559	-0,5466	857	1	-0,2875	9505	1	0,0516	1039	1	0,3391
ICT1	2354	-0,1781	489	-0,2023	762	-0,4652	860	1	-0,2871	14056	1	-0,0242	1533	1	0,2629
MAST4	2499	-0,1718	610	-0,1837	783	-0,4588	861	1	-0,2870	18796	1	-0,0119	1420	1	0,2751
CHST7	1450	0,2428	3816	0,0692	598	0,5285	869	1	0,2857	2946	1	-0,1736	604	1	-0,4592
AP1S2	446	0,4602	485	0,2031	270	0,7455	872	1	0,2852	1575	1	-0,2571	443	1	-0,5423
LRRC23	9901	-0,0473	2999	-0,0825	1352	-0,3321	877	1	-0,2848	11729	1	-0,0351	1642	1	0,2497
SEPT9	1967	-0,1981	265	-0,2550	725	-0,4820	881	1	-0,2839	8939	1	-0,0568	1903	1	0,2270
GALM	1206	-0,2696	234	-0,2683	542	-0,5519	893	1	-0,2823	28691	1	0,0013	1368	1	0,2835
SRPX	242	0,5936	3012	0,0822	176	0,8757	895	1	0,2821	413	1	-0,5114	169	1	-0,7935
CDH1	244	-0,5923	930	-0,1542	178	-0,8742	897	1	-0,2819	584	1	0,4380	227	1	0,7199
SHMT1	12121	-0,0335	1618	-0,1184	1482	-0,3131	909	1	-0,2796	6674	1	-0,0849	2322	1	0,1947
OLFML3	4195	0,1207	83	0,3918	1011	0,4001	910	1	0,2795	1434	1	0,2711	21832	1	-0,0083
AMZ2	2584	-0,1686	586	-0,1869	830	-0,4478	913	1	-0,2792	16017	1	-0,0184	1549	1	0,2609
ANXA8L1	2620	0,1672	3457	0,0746	837	0,4464	916	1	0,2791	6189	1	-0,0926	886	1	-0,3717
CYB5A	712	-0,3621	6508	-0,0406	384	-0,6407	923	1	-0,2785	1033	1	0,3215	348	1	0,6001
CAMK2D	363	-0,5103	673	-0,1770	228	-0,7874	938	1	-0,2770	973	1	0,3334	334	1	0,6104
TGFB1I1	459	0,4484	263	0,2566	288	0,7225	958	1	0,2741	2550	1	-0,1918	588	1	-0,4659
CPM	4802	-0,1078	5494	-0,0494	1083	-0,3813	964	1	-0,2736	8812	1	0,0584	1071	1	0,3320
MTMR4	6103	-0,0874	462	-0,2061	1192	-0,3610	965	1	-0,2735	4802	1	-0,1186	3172	1	0,1549
C1orf116	180	-0,6674	222	-0,2732	142	-0,9404	969	1	-0,2730	721	1	0,3942	279	1	0,6671
FAM107B	952	-0,3072	2732	-0,0873	482	-0,5800	971	1	-0,2728	2043	1	0,2200	529	1	0,4927
VEGFA	103	0,7671	123	0,3499	99	1,0396	975	1	0,2725	641	1	-0,4172	256	1	-0,6897
CHP1	824	-0,3392	865	-0,1598	425	-0,6112	981	1	-0,2720	2819	1	0,1795	628	1	0,4515
RPPH1	518	0,4259	666	0,1779	4089	0,1549	990	1	-0,2710	1669	1	-0,2480	14489	1	0,0230
SLC39A13	901	0,3199	3547	0,0732	464	0,5907	991	1	0,2708	1682	1	-0,2467	488	1	-0,5175
KLF5	2225	-0,1843	312	-0,2403	807	-0,4536	1002	1	-0,2693	9020	1	-0,0560	2048	1	0,2133
MPP1	7430	-0,0706	559	-0,1913	1299	-0,3389	1010	1	-0,2683	4713	1	-0,1206	3374	1	0,1476
LARGE	1922	0,2015	2504	0,0928	755	0,4689	1021	1	0,2674	5280	1	-0,1087	864	1	-0,3760
IGDCC4	2152	-0,1880	1157	-0,1407	798	-0,4553	1022	1	-0,2673	10010	1	0,0472	1161	1	0,3145
FGL1	6655	-0,0799	1296	-0,1328	1258	-0,3468	1028	1	-0,2669	9365	1	-0,0529	2041	1	0,2141
ALDH3B1	724	-0,3597	7809	-0,0310	406	-0,6260	1033	1	-0,2663	1001	1	0,3287	353	1	0,5950

ANKEF1	378	-0,4955	5151	-0,0527	255	-0,7614	1035	1	-0,2659	570	1	0,4428	240	1	0,7088
SETBP1	552	0,4130	1610	0,1189	335	0,6779	1043	1	0,2648	1215	1	-0,2941	418	1	-0,5590
SNORA23	962	0,3062	6742	0,0386	11626	0,0422	1049	1	-0,2640	1457	1	-0,2676	24881	1	-0,0036
RP11-674N23.1	872	-0,3265	1309	-0,1320	466	-0,5904	1051	1	-0,2639	2499	1	0,1945	609	1	0,4584
PECR	5774	-0,0923	3877	-0,0681	1212	-0,3561	1053	1	-0,2638	14054	1	0,0242	1329	1	0,2879
FAM126A	2004	0,1953	1082	0,1442	784	0,4586	1058	1	0,2632	9575	1	-0,0511	1163	1	-0,3143
THRA	10243	-0,0448	696	-0,1744	1523	-0,3075	1065	1	-0,2627	4331	1	-0,1296	3880	1	0,1332
PDGFD	702	-0,3650	1849	-0,1106	404	-0,6266	1070	1	-0,2616	1600	1	0,2544	492	1	0,5160
HR	7866	-0,0659	6523	-0,0404	1386	-0,3273	1074	1	-0,2615	13771	1	0,0254	1337	1	0,2869
MCFD2	1835	0,2077	1368	0,1297	756	0,4687	1077	1	0,2610	7123	1	-0,0780	1043	1	-0,3391
FCGBP	6260	-0,0851	2110	-0,1029	1264	-0,3460	1082	1	-0,2608	16193	1	-0,0177	1712	1	0,2431
EVA1C	1101	-0,2843	6302	-0,0421	563	-0,5448	1087	1	-0,2605	1742	1	0,2421	508	1	0,5026
IFT81	1610	-0,2253	1648	-0,1173	712	-0,4844	1098	1	-0,2591	5316	1	0,1080	911	1	0,3671
GPX4	5469	-0,0970	2799	-0,0860	1214	-0,3557	1101	1	-0,2587	19086	1	0,0110	1469	1	0,2697
PLAU	47	0,9648	368	0,2241	58	1,2225	1111	1	0,2577	160	1	-0,7407	73	1	-0,9984
KALRN	566	0,4094	4472	0,0602	348	0,6671	1112	1	0,2577	892	1	-0,3493	337	1	-0,6069
ZBTB38	1270	0,2614	386	0,2205	623	0,5183	1120	1	0,2569	10806	1	-0,0409	1274	1	-0,2978
AVPI1	263	-0,5743	987	-0,1500	201	-0,8309	1124	1	-0,2565	623	1	0,4243	268	1	0,6808
SOX2	910	-0,3176	1029	-0,1472	497	-0,5737	1128	1	-0,2561	3047	1	0,1705	696	1	0,4266
NTRK3	7661	0,0682	9	0,7829	1408	0,3241	1129	1	0,2560	185	1	0,7147	607	1	0,4588
FA2H	175	-0,6742	913	-0,1555	145	-0,9291	1140	1	-0,2549	401	1	0,5187	183	1	0,7735
KDSR	979	0,3036	7687	0,0319	525	0,5582	1145	1	0,2546	1427	1	-0,2717	468	1	-0,5263
SLC16A5	1552	-0,2321	1358	-0,1300	708	-0,4858	1160	1	-0,2537	5629	1	0,1021	961	1	0,3558
VCL	1189	0,2712	139	0,3334	604	0,5248	1161	1	0,2537	8456	1	0,0622	2388	1	-0,1914
SYNDIG1	3760	0,1314	5501	0,0493	1064	0,3848	1164	1	0,2534	6852	1	-0,0820	1056	1	-0,3355
NXN	564	-0,4096	4708	-0,0575	353	-0,6627	1166	1	-0,2531	874	1	0,3521	340	1	0,6053
DUSP4	429	-0,4688	3790	-0,0696	289	-0,7214	1170	1	-0,2526	700	1	0,3992	297	1	0,6518
MRPS23	2547	-0,1701	309	-0,2417	912	-0,4225	1171	1	-0,2525	7606	1	-0,0717	2588	1	0,1808
KCTD11	461	0,4480	20042	0,0038	314	0,7004	1173	1	0,2524	565	1	-0,4443	253	1	-0,6966
FXYD5	627	0,3879	3772	0,0699	385	0,6402	1174	1	0,2523	1054	1	-0,3180	397	1	-0,5702
FSTL4	2808	-0,1599	1507	-0,1234	969	-0,4117	1177	1	-0,2519	11509	1	0,0364	1324	1	0,2883
ZC3H12A	2599	0,1680	1191	0,1387	921	0,4198	1178	1	0,2518	12830	1	-0,0293	1386	1	-0,2811
ITGB3	355	0,5140	5858	0,0460	249	0,7657	1180	1	0,2517	505	1	-0,4680	228	1	-0,7197

PFKFB4	1827	0,2084	14616	0,0087	780	0,4594	1190	1	0,2511	2395	1	-0,1997	631	1	-0,4508
CD274	2573	0,1690	1187	0,1389	927	0,4195	1196	1	0,2505	12675	1	-0,0302	1389	1	-0,2806
PIEZO2	8882	-0,0557	1558	-0,1211	1549	-0,3054	1207	1	-0,2497	8165	1	-0,0654	2527	1	0,1844
SELM	1377	0,2500	4674	0,0578	667	0,4994	1213	1	0,2495	2541	1	-0,1922	656	1	-0,4417
KDELC2	1566	0,2311	954	0,1520	732	0,4802	1215	1	0,2490	7037	1	-0,0791	1094	1	-0,3282
GPRIN2	2889	-0,1566	2753	-0,0867	997	-0,4043	1228	1	-0,2477	7768	1	0,0699	1147	1	0,3176
MUC5B	464	-0,4472	329	-0,2365	320	-0,6948	1231	1	-0,2477	2213	1	0,2107	610	1	0,4584
UNC5B	7235	0,0727	10990	0,0167	1437	0,3203	1232	1	0,2476	9029,5	1	-0,0559	1233	1	-0,3036
NAGLU	5974	-0,0896	770	-0,1666	1314	-0,3371	1234	1	-0,2475	7185	1	-0,0770	2811	1	0,1705
KANK1	1856	-0,2062	199	-0,2848	810	-0,4524	1258	1	-0,2462	7078	1	-0,0786	2864	1	0,1676
RP11-479G22.8	1365	0,2508	514	0,1978	675	0,4968	1260	1	0,2460	9362	1	-0,0529	1266	1	-0,2989
C3orf52	132	0,7230	14618	0,0087	127	0,9689	1263	1	0,2459	186	1	-0,7144	84	1	-0,9603
G3BP1	4227	0,1200	946	0,1529	1174	0,3649	1270	1	0,2449	12131	1	0,0329	2062	1	-0,2120
SIRT7	2594	-0,1683	1141	-0,1413	965	-0,4125	1280	1	-0,2442	13413	1	0,0270	1453	1	0,2712
MRPS7	3573	-0,1361	274	-0,2525	1089	-0,3801	1284	1	-0,2440	4906	1	-0,1164	4082	1	0,1276
TC2N	2578	-0,1688	1298	-0,1327	967	-0,4118	1292	1	-0,2430	11573	1	0,0360	1395	1	0,2791
TRAPPC6A	17072	-0,0161	1227	-0,1365	1989	-0,2587	1293	1	-0,2427	4722	1	-0,1204	4312	1	0,1222
TMEM184A	8503	-0,0589	5701	-0,0475	1576	-0,3013	1297	1	-0,2424	18957	1	0,0114	1602	1	0,2538
SQRDL	1641	-0,2223	4465	-0,0602	763	-0,4647	1298	1	-0,2424	3263	1	0,1621	759	1	0,4044
ASF1A	4759	0,1088	4250	0,0628	1237	0,3506	1301	1	0,2419	10158	1	-0,0460	1331	1	-0,2878
ANKRD13A	1439	0,2442	1671	0,1166	711	0,4853	1310	1	0,2411	4407	1	-0,1277	900	1	-0,3688
PLA2G16	1125	-0,2807	388	-0,2200	613	-0,5213	1316	1	-0,2406	8589	1	0,0607	1248	1	0,3013
GDF15	139	-0,7167	187	-0,2971	132	-0,9572	1317	1	-0,2406	633	1	0,4196	285	1	0,6601
IL15	606	0,3943	483	0,2034	396	0,6347	1322	1	0,2404	2571	1	-0,1909	688	1	-0,4313
AXIN2	16749	-0,0174	4912	-0,0552	2011	-0,2571	1333	1	-0,2397	11285	1	-0,0378	2212	1	0,2019
ABHD15	9472	-0,0506	1283	-0,1335	1687	-0,2900	1341	1	-0,2394	6783	1	-0,0829	3128	1	0,1565
UPP1	279	-0,5623	4818	-0,0562	219	-0,8012	1349	1	-0,2389	429	1	0,5062	208	1	0,7450
MXD1	1877	0,2049	17234	0,0056	847	0,4435	1352	1	0,2386	2404	1	-0,1993	665	1	-0,4379
ZNF697	545	0,4160	6444	0,0411	366	0,6545	1353	1	0,2385	779	1	-0,3750	329	1	-0,6134
SEC14L1	4718	-0,1098	442	-0,2099	1254	-0,3474	1363	1	-0,2376	5721	1	-0,1002	3728	1	0,1374
AP000479.1	6267	0,0851	24711	0,0021	1419	0,3224	1367	1	0,2373	6782	1	-0,0829	1129	1	-0,3203
MB21D2	1748	0,2148	601	0,1853	814	0,4517	1371	1	0,2370	12816	1	-0,0294	1503	1	-0,2664
GATM	9606	-0,0493	833	-0,1622	1724	-0,2860	1374	1	-0,2367	5093	1	-0,1130	4237	1	0,1238

KCNMA1	995	0,3014	1747	0,1139	581	0,5377	1378	1	0,2363	2642	1	-0,1874	709	1	-0,4238
PRKD3	1749	0,2147	1013	0,1480	822	0,4500	1390	1	0,2353	8052	1	-0,0667	1240	1	-0,3020
MRPL10	4949	-0,1054	203	-0,2817	1294	-0,3405	1395	1	-0,2350	2882	1	-0,1763	8578	1	0,0588
CASC15	6299	0,0847	10557	0,0182	1446	0,3193	1404	1	0,2346	8072	1	-0,0665	1250	1	-0,3011
NDST1	510	0,4295	1687	0,1159	352	0,6638	1409	1	0,2343	1084	1	-0,3136	432	1	-0,5479
FGA	3030	-0,1523	948	-0,1526	1058	-0,3866	1410	1	-0,2342	29594	1	-0,0002	1820	1	0,2340
ANXA8L2	2095	0,1909	5667	0,0478	906	0,4248	1412	1	0,2339	3844	1	-0,1431	859	1	-0,3770
CACNA1G	374	-0,4994	736	-0,1703	278	-0,7330	1417	1	-0,2337	999	1	0,3291	411	1	0,5628
PFKFB3	3483	-0,1386	231	-0,2691	1131	-0,3722	1419	1	-0,2336	4296	1	-0,1305	5259	1	0,1031
LINGO2	20936	0,0093	6753	0,0385	2199	0,2428	1421	1	0,2335	12838	1	0,0292	2169	1	-0,2042
CUEDC1	3445	-0,1396	597	-0,1857	1126	-0,3731	1422	1	-0,2335	10144	1	-0,0461	2461	1	0,1874
GDF6	218	0,6249	12044	0,0136	185	0,8573	1439	1	0,2324	265	1	-0,6113	137	1	-0,8437
SLC6A17	888	0,3230	4288	0,0624	533	0,5549	1443	1	0,2319	1536	1	-0,2606	531	1	-0,4925
SEMA4B	791	-0,3439	5041	-0,0539	490	-0,5756	1448	1	-0,2317	1252	1	0,2900	477	1	0,5217
SLC25A1	4757	-0,1089	506	-0,1993	1295	-0,3401	1454	1	-0,2313	6318	1	-0,0904	3593	1	0,1409
ACP2	662	0,3777	9505	0,0223	430	0,6089	1456	1	0,2312	860	1	-0,3554	364	1	-0,5866
AGFG2	445	-0,4610	470	-0,2048	322	-0,6917	1464	1	-0,2307	1588	1	0,2562	548	1	0,4869
FAM102A	2344	-0,1785	319	-0,2389	982	-0,4092	1465	1	-0,2307	8623	1	-0,0604	2816	1	0,1703
ROBO1	802	0,3429	2773	0,0864	498	0,5731	1471	1	0,2302	1582	1	-0,2565	550	1	-0,4867
FGG	6346	-0,0841	1536	-0,1222	1476	-0,3142	1474	1	-0,2301	11234	1	-0,0381	2379	1	0,1920
C8orf47	7263	-0,0725	2713	-0,0877	1568	-0,3026	1475	1	-0,2301	17226	1	-0,0151	2031	1	0,2149
ARHGAP27	4878	-0,1068	4677	-0,0577	1317	-0,3367	1480	1	-0,2299	9810	1	0,0490	1397	1	0,2789
GSTM3	5172	-0,1017	1552	-0,1213	1370	-0,3302	1494	1	-0,2285	15312	1	-0,0196	2103	1	0,2089
C1orf115	2163	-0,1877	3265	-0,0774	948	-0,4157	1505	1	-0,2280	5202	1	0,1103	1050	1	0,3383
SLC39A11	10251	-0,0447	2473	-0,0935	1847	-0,2719	1517	1	-0,2272	9840	1	-0,0488	2643	1	0,1784
SSBP3	219	0,6246	2721	0,0876	190	0,8513	1530	1	0,2267	373	1	-0,5370	187	1	-0,7637
COL5A1	73	0,8317	4229	0,0630	93	1,0577	1544	1	0,2259	146	1	-0,7687	74	1	-0,9946
BLVRB	3290	-0,1438	9750	-0,0212	1154	-0,3692	1552	1	-0,2253	4621	1	0,1227	993	1	0,3480
SLFNL1	1062	0,2906	9195	0,0235	630	0,5157	1555	1	0,2251	1465	1	-0,2671	532	1	-0,4922
CAV2	1283	-0,2594	2466	-0,0937	718	-0,4837	1572	1	-0,2243	3171	1	0,1657	803	1	0,3901
MAMLD1	6028	0,0886	1159	0,1405	1484	0,3127	1578	1	0,2240	9478	1	0,0518	2767	1	-0,1722
CASP4	3182	-0,1471	1562	-0,1207	1147	-0,3701	1590	1	-0,2230	13568	1	0,0264	1647	1	0,2494
DRD1	10522	-0,0428	2763	-0,0866	1917	-0,2655	1593	1	-0,2228	10443	1	-0,0438	2626	1	0,1789

SNAI2	2948	0,1547	3313	0,0767	1103	0,3773	1595	1	0,2226	7122	1	-0,0780	1254	1	-0,3006
PADI2	1284	-0,2594	727	-0,1711	726	-0,4818	1600	1	-0,2225	6446	1	0,0882	1191	1	0,3107
AK3P5	12666	0,0303	12824	0,0118	2066	0,2526	1607	1	0,2223	15684	1	-0,0186	1731	1	-0,2408
PGPEP1	7101	-0,0741	5269	-0,0515	1629	-0,2962	1614	1	-0,2221	14464	1	0,0226	1692	1	0,2447
PDGFRL	1065	-0,2901	1098	-0,1434	639	-0,5119	1621	1	-0,2218	3736	1	0,1467	904	1	0,3685
DFNA5	7181	0,0733	717	0,1727	1643	0,2945	1626	1	0,2212	5764	1	0,0994	4328	1	-0,1218
BCL3	1981	-0,1969	4481	-0,0600	937	-0,4181	1629	1	-0,2211	4067	1	0,1369	951	1	0,3580
TMEM178B	7414	-0,0709	1885	-0,1096	1668	-0,2918	1631	1	-0,2209	11148	1	-0,0387	2560,5	1	0,1822
LRIG3	1234	0,2653	6938	0,0371	707	0,4860	1636	1	0,2207	1923	1	-0,2282	636	1	-0,4489
FBXO6	7043	-0,0748	4535	-0,0595	1640	-0,2947	1644	1	-0,2199	17043	1	0,0153	1800	1	0,2352
TMC6	985	-0,3031	889	-0,1573	610	-0,5219	1660	1	-0,2188	3761	1	0,1458	919	1	0,3646
TLCD1	1820	-0,2090	6269	-0,0425	898	-0,4275	1666	1	-0,2185	3148	1	0,1666	826	1	0,3851
URB1	703	0,3649	5921	0,0454	479	0,5833	1669	1	0,2184	1043	1	-0,3195	448	1	-0,5379
UNC93B1	798	-0,3435	356	-0,2269	516	-0,5613	1677	1	-0,2178	4899	1	0,1166	1061	1	0,3344
PPM1H	726	-0,3593	19310	-0,0042	488	-0,5767	1683	1	-0,2173	862	1	0,3551	392	1	0,5725
NHS	382	0,4914	806	0,1639	305	0,7087	1686	1	0,2172	1008	1	-0,3275	437	1	-0,5448
ACSS2	2851	-0,1580	706	-0,1734	1114	-0,3746	1697	1	-0,2166	17034	1	-0,0153	2221	1	0,2012
BIN1	69	-0,8431	424	-0,2135	90	-1,0597	1698	1	-0,2166	240	1	0,6296	135	1	0,8462
RAB3D	1434	-0,2446	2899	-0,0843	776	-0,4611	1700	1	-0,2165	3318	1	0,1603	860	1	0,3768
MCU	1239	-0,2647	753	-0,1681	729	-0,4809	1708	1	-0,2162	5938	1	0,0965	1174	1	0,3127
SAMD4A	12411	0,0319	291	0,2462	2131	0,2479	1711	1	0,2160	2144	1	0,2143	28485	1	-0,0017
PIK3C2B	1404	-0,2476	6383	-0,0416	771	-0,4631	1721	1	-0,2155	2285	1	0,2060	717	1	0,4215
LIMS1	602	0,3954	685	0,1756	426	0,6108	1722	1	0,2154	2046	1	-0,2197	670	1	-0,4352
SPTSSA	7613	-0,0688	632	-0,1817	1749	-0,2836	1733	1	-0,2148	5094	1	-0,1129	5334	1	0,1018
HMGCS1	5879	-0,0910	118	-0,3540	1544	-0,3055	1740	1	-0,2145	1515	1	-0,2630	9788	1	-0,0485
CEP170	840	0,3345	19246	0,0043	555	0,5483	1755	1	0,2139	990	1	-0,3302	440	1	-0,5440
KMT2C	5795	0,0921	828	0,1624	1553	0,3049	1779	1	0,2129	7727	1	0,0703	3554	1	-0,1425
CTGF	10	1,4510	1554	0,1213	18	1,6637	1783	1	0,2127	20	1	-1,3297	12	1	-1,5425
FLOT2	3507	-0,1381	6063	-0,0442	1236	-0,3507	1784	1	-0,2126	6099	1	0,0939	1215	1	0,3065
ABCG2	859	-0,3299	1421	-0,1273	572	-0,5420	1793	1	-0,2120	2347	1	0,2026	733	1	0,4147
KCNK1	2046	-0,1930	778	-0,1658	996	-0,4050	1794	1	-0,2120	13353	1	0,0272	1749	1	0,2392
G6PD	833	-0,3372	4546	-0,0594	551	-0,5490	1798	1	-0,2118	1372	1	0,2779	539	1	0,4896
RBM47	642	-0,3834	2347	-0,0966	454	-0,5951	1799	1	-0,2117	1287	1	0,2867	518	1	0,4985

ITGB8	946	-0,3087	538	-0,1946	620	-0,5193	1809	1	-0,2107	5035	1	0,1141	1105	1	0,3247
NCOA7	120	-0,7438	338	-0,2329	136	-0,9540	1815	1	-0,2102	414	1	0,5109	226	1	0,7211
TJP2	894	-0,3221	4579	-0,0589	592	-0,5319	1821	1	-0,2099	1514	1	0,2632	568	1	0,4731
SMAD7	870	0,3270	10860	0,0170	583	0,5366	1826	1	0,2096	1114	1	-0,3100	484	1	-0,5196
PSMB3	20056	-0,0100	157	-0,3158	2562	-0,2189	1836	1	-0,2089	1136	1	-0,3058	5606	1	-0,0969
ADAM12	786	0,3446	1369	0,1296	539	0,5535	1837	1	0,2089	2128	1	-0,2150	708	1	-0,4239
ZC4H2	8802	0,0563	14420	0,0091	1922	0,2649	1841	1	0,2087	10020	1	-0,0472	1588	1	-0,2558
IL32	5436	0,0975	1090	0,1438	1551	0,3051	1858	1	0,2076	10103	1	0,0464	3013	1	-0,1613
MINK1	12262	-0,0327	1025	-0,1475	2248	-0,2398	1866	1	-0,2072	5002	1	-0,1148	5910	1	0,0924
PCNX	717	0,3607	5791	0,0467	509	0,5678	1867	1	0,2072	1081	1	-0,3140	479	1	-0,5212
IFI44	9612	0,0493	9491	0,0223	2019	0,2563	1870	1	0,2071	13418	1	-0,0269	1821	1	-0,2340
MED15	763	0,3499	3754	0,0701	527	0,5570	1871	1	0,2070	1349	1	-0,2798	549	1	-0,4869
RAET1E	1151	0,2765	6345	0,0418	721	0,4835	1872	1	0,2070	1828	1	-0,2347	655	1	-0,4417
COA3	3759	-0,1314	212	-0,2764	1310	-0,3375	1890	1	-0,2061	3794	1	-0,1449	8335	1	0,0612
RAB13	25392	-0,0053	7601	-0,0324	2690	-0,2113	1893	1	-0,2060	13393	1	-0,0271	2627	1	0,1789
RPL19	9800	-0,0481	262	-0,2570	2052	-0,2539	1894	1	-0,2058	2248	1	-0,2089	26171	1	-0,0031
RBMS3	1052	0,2913	413	0,2153	674	0,4968	1902	1	0,2055	7256	1	-0,0760	1383	1	-0,2815
TM7SF2	10104	-0,0459	2969	-0,0830	2083	-0,2513	1905	1	-0,2054	11377	1	-0,0371	2853	1	0,1682
CUL9	3414	-0,1406	2422	-0,0949	1265	-0,3457	1908	1	-0,2051	10202	1	0,0457	1631	1	0,2508
FERMT2	342	0,5214	751	0,1682	283	0,7263	1909	1	0,2049	868	1	-0,3532	420	1	-0,5581
CHPF2	1522	0,2353	1581	0,1203	855	0,4400	1912	1	0,2047	4991	1	-0,1150	1133	1	-0,3197
FAM64A	914	-0,3173	1572	-0,1205	612	-0,5215	1919	1	-0,2041	2448	1	0,1968	772	1	0,4009
SSH3	7035	-0,0748	2168	-0,1014	1792	-0,2785	1928	1	-0,2037	13500	1	-0,0266	2675	1	0,1771
SLC16A1	4354	0,1172	1258	0,1347	1439	0,3202	1944	1	0,2030	16399	1	0,0175	2497	1	-0,1855
ZEB1	1408	0,2472	4075	0,0653	819	0,4502	1946	1	0,2030	2770	1	-0,1820	828	1	-0,3849
EML2	3237	-0,1452	4923	-0,0551	1249	-0,3482	1951	1	-0,2029	6334	1	0,0901	1298	1	0,2930
NOG	1272	0,2612	25327	0,0019	768	0,4636	1959	1	0,2025	1548	1	-0,2593	597	1	-0,4618
NT5C3B	4971	-0,1051	414	-0,2148	1522	-0,3075	1960	1	-0,2024	5232	1	-0,1097	5885	1	0,0927
RAB15	1829	-0,2083	243	-0,2640	980	-0,4100	1983	1	-0,2016	9056	1	-0,0557	3435	1	0,1460
THBD	1200	0,2700	1822	0,1113	750	0,4704	2017	1	0,2003	3364	1	-0,1587	948	1	-0,3591
KCNA7	1470	0,2408	27142	0,0006	851	0,4410	2021	1	0,2002	1763	1	-0,2402	657	1	-0,4404
RP11-806O11.1	5811	-0,0918	2333	-0,0970	1667	-0,2918	2031	1	-0,2000	24159	1	-0,0052	2321	1	0,1948
CHST11	537	0,4188	17153	0,0058	420	0,6173	2063	1	0,1985	654	1	-0,4129	331	1	-0,6115

FCHO1	28271	-0,0002	1294	-0,1329	2922	-0,1987	2064	1	-0,1985	4214	1	-0,1328	7889	1	0,0657
IRS2	619	-0,3903	924	-0,1547	473	-0,5886	2070	1	-0,1982	1815	1	0,2357	675	1	0,4339
PPFIBP2	93	-0,7945	382	-0,2212	117	-0,9919	2089	1	-0,1974	314	1	0,5733	184	1	0,7707
OSBPL8	728	0,3590	1661	0,1170	530	0,5563	2092	1	0,1973	1743	1	-0,2421	660	1	-0,4393
SHROOM2	975	0,3040	7218	0,0350	658	0,5009	2096	1	0,1968	1449	1	-0,2691	587	1	-0,4659
ARHGAP24	3398	-0,1411	1829	-0,1111	1307	-0,3377	2100	1	-0,1966	12710	1	0,0300	1907	1	0,2266
SLC35F2	1479	0,2393	4220	0,0631	865	0,4358	2105	1	0,1964	2883	1	-0,1762	879	1	-0,3727
CLIC4	1501	0,2372	641	0,1814	875	0,4334	2110	1	0,1962	9045	1	-0,0558	1616	1	-0,2520
STEAP3	595	0,3972	2061	0,1042	460	0,5931	2119	1	0,1959	1225	1	-0,2930	541	1	-0,4889
CHD5	740	0,3566	1583	0,1202	541	0,5522	2127	1	0,1956	1809	1	-0,2363	684	1	-0,4320
NCR3LG1	3362	0,1425	2469	0,0936	1306	0,3378	2133	1	0,1953	9828	1	-0,0489	1698	1	-0,2443
ALDH3A2	609	-0,3937	815	-0,1631	471	-0,5890	2139	1	-0,1952	1885	1	0,2307	698	1	0,4259
CHD7	3629	0,1346	2384	0,0956	1371	0,3298	2140	1	0,1951	11085	1	-0,0391	1815	1	-0,2342
LMTK3	2246	-0,1834	1845	-0,1107	1096	-0,3785	2144	1	-0,1951	7520	1	0,0728	1489	1	0,2678
CTD-2267D19.3	9122	-0,0535	2313	-0,0975	2128	-0,2484	2145	1	-0,1949	10424	1	-0,0439	3294	1	0,1510
MEX3B	425	0,4708	3036	0,0818	349	0,6656	2147	1	0,1948	735	1	-0,3890	368	1	-0,5838
COBL	1321	-0,2558	3952	-0,0669	820	-0,4501	2159	1	-0,1943	2603	1	0,1890	832	1	0,3833
SPATA17	26888	-0,0024	5861	-0,0460	2987	-0,1958	2179	1	-0,1934	10466	1	-0,0436	3314	1	0,1499
GALNT18	701	0,3652	860	0,1599	526	0,5580	2192	1	0,1928	2301	1	-0,2053	777	1	-0,3981
MPP4	12483	0,0313	8476	0,0273	2476	0,2236	2204	1	0,1923	25890	1	-0,0040	2289	1	-0,1963
PHF17	1337	0,2542	782	0,1655	838	0,4464	2208	1	0,1921	6418	1	-0,0887	1388	1	-0,2808
SYNGR2	915	-0,3172	96	-0,3789	645	-0,5092	2213	1	-0,1920	8505	1	-0,0617	3975	1	0,1303
CPNE4	3810	-0,1303	1501	-0,1239	1424	-0,3220	2219	1	-0,1917	23243	1	0,0064	2263	1	0,1981
EVA1A	594	0,3977	1722	0,1147	470	0,5891	2224	1	0,1914	1320	1	-0,2829	565	1	-0,4744
SLC9C1	2350	0,1782	25518	0,0018	1151	0,3696	2228	1	0,1914	2879	1	-0,1764	910	1	-0,3678
RP4-794H19.1	1436	0,2445	18449	0,0050	867	0,4357	2233	1	0,1912	1775	1	-0,2394	689	1	-0,4306
PEA15	293	0,5532	617	0,1833	271	0,7441	2237	1	0,1909	799	1	-0,3699	417	1	-0,5608
PLEKHH3	2961	-0,1543	4555	-0,0592	1273	-0,3448	2247	1	-0,1905	6029	1	0,0951	1346	1	0,2856
SH3KBP1	150	0,7051	1210	0,1377	165	0,8950	2263	1	0,1900	321	1	-0,5674	193	1	-0,7574
C18orf25	2107	0,1904	10762	0,0174	1091	0,3795	2276	1	0,1892	2968	1	-0,1730	931	1	-0,3621
PYCARD	27981	-0,0011	3941	-0,0670	3149	-0,1890	2307	1	-0,1879	8117	1	-0,0659	4325	1	0,1220
QPCT	249	0,5883	721	0,1718	242	0,7761	2308	1	0,1878	642	1	-0,4165	341	1	-0,6043
PLEKHO1	1742	0,2152	1918	0,1088	1000	0,4030	2313	1	0,1878	5385	1	-0,1064	1293	1	-0,2941

RPH3AL	3451	-0,1395	716	-0,1728	1391	-0,3270	2322	1	-0,1874	12082	1	-0,0332	3191	1	0,1542
CMBL	3309	-0,1435	4863	-0,0558	1362	-0,3309	2323	1	-0,1874	6481	1	0,0877	1419	1	0,2751
LTBR	993	-0,3016	4034	-0,0657	700	-0,4883	2337	1	-0,1867	1812	1	0,2358	712	1	0,4226
SH2D3A	2266	-0,1822	11460	-0,0151	1155	-0,3687	2339	1	-0,1865	3135	1	0,1672	969	1	0,3537
WDR91	3135	0,1489	9993	0,0201	1330	0,3348	2346	1	0,1859	4359	1	-0,1288	1158	1	-0,3147
GSTA4	10863	-0,0404	11380	-0,0154	2430	-0,2261	2350	1	-0,1857	13865	1	0,0251	2078	1	0,2108
LGR6	2833	-0,1587	2048	-0,1045	1279	-0,3443	2351	1	-0,1856	9236	1	0,0541	1741	1	0,2397
FAM102B	1421	0,2461	5105	0,0531	887	0,4315	2360	1	0,1853	2527	1	-0,1930	854	1	-0,3783
SLC16A2	579	0,4047	232	0,2690	468	0,5900	2361	1	0,1853	4110	1	-0,1356	1122	1	-0,3209
ZNF462	5015	0,1042	216	0,2753	1693	0,2894	2363	1	0,1853	3025	1	0,1711	17621	1	-0,0142
CTD-2207P18.2	6462	0,0824	8390	0,0277	1902	0,2669	2387	1	0,1846	9163	1	-0,0546	1747	1	-0,2392
OSBP2	600	-0,3958	9044	-0,0243	484	-0,5795	2403	1	-0,1837	790	1	0,3715	425	1	0,5552
DKK1	431	-0,4677	130	-0,3399	369	-0,6507	2415	1	-0,1829	4394	1	0,1279	1190	1	0,3108
RDH10	621	-0,3889	147	-0,3228	499	-0,5718	2416	1	-0,1828	8092	1	0,0662	1649	1	0,2490
TACO1	2831	-0,1588	602	-0,1853	1300	-0,3388	2473	1	-0,1800	13557	1	-0,0264	3209	1	0,1536
CYB5B	841	-0,3342	119	-0,3534	634	-0,5141	2475	1	-0,1799	15518	1	-0,0192	3034	1	0,1607
EPHB2	671	0,3748	2944	0,0835	534	0,5545	2479	1	0,1797	1241	1	-0,2913	571	1	-0,4710
DBP	2302	-0,1803	3715	-0,0706	1197	-0,3596	2489	1	-0,1794	5235	1	0,1097	1319	1	0,2890
CREB3L2	1534	0,2339	181	0,2994	963	0,4130	2501	1	0,1791	8147	1	0,0656	4724	1	-0,1136
ABCC1	533	-0,4203	5018	-0,0542	443	-0,5991	2511	1	-0,1788	810	1	0,3661	436	1	0,5449
CCDC50	779	0,3458	707	0,1733	605	0,5246	2512	1	0,1788	2978	1	-0,1725	979	1	-0,3513
SSR3	969	0,3049	2220	0,1000	722	0,4833	2521	1	0,1784	2312	1	-0,2049	831	1	-0,3833
AMOTL1	14541	-0,0225	191	-0,2924	2892	-0,2007	2527	1	-0,1782	1445	1	-0,2699	5949	1	-0,0916
GLP2R	727	-0,3592	1268	-0,1341	585	-0,5362	2553	1	-0,1769	1973	1	0,2251	767	1	0,4020
GCSAM	655	0,3800	25633	0,0016	528	0,5569	2554	1	0,1769	766	1	-0,3784	424	1	-0,5553
TMEM165	1599	0,2262	2889	0,0845	1002	0,4027	2559	1	0,1765	3904	1	-0,1417	1143	1	-0,3182
EVPL	1344	-0,2536	2938	-0,0835	891	-0,4300	2564	1	-0,1764	3057	1	0,1700	1002	1	0,3465
PLEKHH2	7653	-0,0683	1972	-0,1068	2173	-0,2447	2566	1	-0,1764	11182	1	-0,0385	3713	1	0,1379
IFNLR1	9796	-0,0481	3856	-0,0686	2467	-0,2244	2571	1	-0,1762	15118	1	-0,0204	3141	1	0,1558
RASSF9	5746	-0,0927	2202	-0,1005	1883	-0,2689	2572	1	-0,1761	22068	1	-0,0078	2851	1	0,1684
HIVEP2	303	0,5486	3155	0,0796	284	0,7247	2573	1	0,1761	498	1	-0,4690	302	1	-0,6451
RAP1GAP	1055	-0,2910	196	-0,2875	760	-0,4657	2605	1	-0,1747	26712	1	0,0035	2649	1	0,1782
PIM1	5037	-0,1038	7496	-0,0331	1794	-0,2785	2608	1	-0,1746	7706	1	0,0707	1682	1	0,2453

KLF7	845	0,3332	10455	0,0185	647	0,5077	2612	1	0,1745	1075	1	-0,3147	540	1	-0,4892
STON2	92	0,7955	14136	0,0097	126	0,9689	2646	1	0,1734	133	1	-0,7858	85	1	-0,9592
MAMDC2	16252	0,0185	16535	0,0066	3093	0,1912	2664	1	0,1727	18741,5	1	-0,0119	2523	1	-0,1847
MTHFR	5546	-0,0958	3505	-0,0739	1890	-0,2683	2671	1	-0,1725	14711	1	0,0219	2330	1	0,1944
B4GALT1	891	0,3224	3000	0,0824	682	0,4945	2679	1	0,1721	1768	1	-0,2399	742	1	-0,4120
TYRO3	551	0,4135	1484	0,1246	477	0,5855	2685	1	0,1720	1267	1	-0,2888	599	1	-0,4609
PALLD	151	0,7023	91	0,3828	179	0,8736	2702	1	0,1713	1042	1	-0,3195	535	1	-0,4909
LRP11	624	0,3882	3684	0,0710	521	0,5590	2714	1	0,1708	1060	1	-0,3172	544	1	-0,4880
GNG4	444	0,4624	5233	0,0519	397	0,6329	2718	1	0,1705	657	1	-0,4105	374	1	-0,5810
PMEPA1	316	0,5382	349	0,2292	306	0,7086	2719	1	0,1704	1123	1	-0,3090	557	1	-0,4794
LAMA4	2432	-0,1743	1955	-0,1075	1278	-0,3443	2735	1	-0,1700	8047	1	0,0668	1778	1	0,2367
EPPK1	26283	0,0033	6667	0,0393	3558	0,1730	2750	1	0,1697	11575	1	0,0360	3862	1	-0,1336
BCHE	17264	-0,0151	3898	-0,0677	3253	-0,1843	2763	1	-0,1693	9381	1	-0,0527	4567	1	0,1166
TPM1	87	0,7980	295	0,2455	129	0,9672	2764	1	0,1692	352	1	-0,5525	225	1	-0,7217
CAV1	283	-0,5611	3412	-0,0752	280	-0,7303	2767	1	-0,1692	466	1	0,4859	292	1	0,6551
STARD13	1243	0,2645	5772	0,0468	877	0,4329	2789	1	0,1684	2080	1	-0,2177	822	1	-0,3861
AC017002.2	7213	0,0729	7406	0,0337	2230	0,2412	2793	1	0,1684	11065	1	-0,0391	2121	1	-0,2075
ACBD4	10017	-0,0465	1974	-0,1068	2621	-0,2148	2795	1	-0,1683	8636	1	-0,0603	4994	1	0,1081
AC106801.1	7851	0,0660	12672	0,0120	2333	0,2338	2811	1	0,1678	9255,5	1	-0,0540	1956	1	-0,2218
TXNDC17	1852	-0,2067	419	-0,2145	1116	-0,3741	2821	1	-0,1675	22053	1	-0,0079	3065	1	0,1596
AC006262.6	705	0,3641	8320	0,0282	595	0,5307	2847	1	0,1666	955	1	-0,3359	509	1	-0,5025
AC012613.2	1014	0,2984	10966	0,0167	764	0,4643	2868	1	0,1660	1332	1	-0,2816	639	1	-0,4476
LIPG	10235	-0,0449	2541	-0,0919	2709	-0,2106	2877	1	-0,1657	10038	1	-0,0470	4466	1	0,1187
JARID2	181	0,6665	2794	0,0861	199	0,8316	2891	1	0,1650	305	1	-0,5805	207	1	-0,7455
VSTM4	117	0,7472	26435	0,0012	480	0,5831	2914	1	-0,1640	155	1	-0,7460	371	1	-0,5819
STK17B	1027	0,2952	4957	0,0549	781	0,4593	2915	1	0,1640	1761	1	-0,2404	760	1	-0,4044
APOC1	9840	-0,0477	3406	-0,0753	2693	-0,2112	2931	1	-0,1635	13172	1	-0,0276	3778	1	0,1359
EGLN3	4858	-0,1071	3488	-0,0742	1865	-0,2703	2944	1	-0,1632	12120	1	0,0330	2293	1	0,1962
ANKRD40	8108	-0,0630	1196	-0,1383	2429	-0,2261	2946	1	-0,1632	7305	1	-0,0753	6170	1	0,0879
HECTD2	1473	0,2404	8304	0,0283	1001	0,4029	2957	1	0,1625	2191	1	-0,2121	874	1	-0,3746
SACS	1565	0,2312	1545	0,1215	1043	0,3933	2965	1	0,1621	5237	1	-0,1096	1446	1	-0,2718
DGKI	15043	0,0213	19045	0,0044	3287	0,1832	2972	1	0,1619	16558	1	-0,0168	2633	1	-0,1787
LTBP2	381	0,4920	8148	0,0291	367	0,6537	2985	1	0,1617	524	1	-0,4629	318	1	-0,6246

ABLIM3	30	1,0930	1968	0,1070	54	1,2544	2995	1	0,1614	55	1	-0,9860	45	1	-1,1474
LPCAT2	128	0,7290	976	0,1507	167	0,8903	3000	1	0,1613	306	1	-0,5783	213	1	-0,7396
C11orf45	7042	-0,0748	2654	-0,0891	2298	-0,2361	3001	1	-0,1613	17635	1	-0,0143	3403	1	0,1470
ITGAV	148	0,7067	2641	0,0894	182	0,8674	3022	1	0,1607	261	1	-0,6173	180	1	-0,7780
TFCP2L1	2386	-0,1767	3491	-0,0741	1318	-0,3367	3039	1	-0,1600	5596	1	0,1026	1535	1	0,2626
FGF2	123	0,7390	527	0,1961	163	0,8989	3041	1	0,1600	363	1	-0,5429	249	1	-0,7028
MAP2K4	1135	-0,2784	556	-0,1917	859	-0,4384	3042	1	-0,1600	6549	1	0,0868	1666	1	0,2467
NT5C2	792	-0,3439	2571	-0,0912	654	-0,5036	3051	1	-0,1597	1621	1	0,2527	738	1	0,4125
HOOK1	5827	-0,0916	4813	-0,0562	2080	-0,2513	3052	1	-0,1597	11686	1	0,0354	2313	1	0,1951
CHDH	4154	-0,1217	3518	-0,0737	1769	-0,2812	3060	1	-0,1595	9936	1	0,0480	2120	1	0,2075
TRIM69	3026	-0,1524	8038	-0,0297	1492	-0,3116	3076	1	-0,1592	4614	1	0,1227	1381	1	0,2819
LMO7	507	-0,4306	2123	-0,1026	469	-0,5897	3078	1	-0,1591	1004	1	0,3281	547	1	0,4872
MAP1B	1908	0,2022	335	0,2335	1191	0,3611	3089	1	0,1588	12436	1	0,0313	4084	1	-0,1275
CD38	659	-0,3783	1748	-0,1139	584	-0,5364	3114	1	-0,1580	1503	1	0,2644	713	1	0,4225
VAMP8	11202	-0,0382	3278	-0,0772	2988	-0,1958	3121	1	-0,1576	11087	1	-0,0391	4472	1	0,1186
PPP1R13L	393	0,4856	3386	0,0756	379	0,6431	3129	1	0,1574	659	1	-0,4100	401	1	-0,5675
E2F7	924	0,3151	6604	0,0398	745	0,4723	3130	1	0,1572	1392	1	-0,2752	681	1	-0,4324
SP140L	10648	0,0420	895	0,1570	2923	0,1986	3145	1	0,1567	4985	1	0,1151	10732	1	-0,0416
NUAK1	323	0,5303	2039	0,1048	329	0,6870	3146	1	0,1567	619	1	-0,4255	370	1	-0,5822
ATP10D	2414	0,1753	2781	0,0863	1364	0,3308	3180	1	0,1556	6399	1	-0,0890	1695	1	-0,2445
TRIM47	2810	-0,1598	2302	-0,0977	1464	-0,3150	3191	1	-0,1553	8475	1	0,0621	2011	1	0,2174
FAH	1667	-0,2208	3458	-0,0746	1110	-0,3758	3206,5	1	-0,1549	3746	1	0,1462	1249	1	0,3012
RHPN1	1668	-0,2208	1428	-0,1270	1111	-0,3758	3206,5	1	-0,1549	6102	1	0,0938	1650	1	0,2487
CLSTN1	345	0,5186	3297	0,0770	342	0,6731	3221	1	0,1545	574	1	-0,4416	352	1	-0,5961
CA11	5961	-0,0898	9097	-0,0240	2181	-0,2439	3240	1	-0,1541	8124	1	0,0658	1979	1	0,2199
CTTNBP2	2793	0,1606	6794	0,0382	1481	0,3132	3294	1	0,1526	4637	1	-0,1224	1422	1	-0,2750
ARHGEF37	5129	-0,1022	3221	-0,0783	2047	-0,2543	3305	1	-0,1521	14176	1	0,0239	2696	1	0,1759
METTL16	2561	-0,1697	259	-0,2581	1426	-0,3216	3309	1	-0,1520	6436	1	-0,0884	8087	1	0,0636
PIR	1256	-0,2630	9987	-0,0202	951	-0,4150	3310	1	-0,1520	1734	1	0,2428	791	1	0,3948
IGSF11	1866	-0,2056	1215	-0,1374	1205	-0,3575	3313	1	-0,1519	7916	1	0,0682	1975	1	0,2201
INA	785	-0,3447	3112	-0,0804	676	-0,4965	3314	1	-0,1519	1505	1	0,2642	726	1	0,4161
FOXA2	470	-0,4456	14689	-0,0086	448	-0,5974	3315	1	-0,1518	588	1	0,4370	362	1	0,5888
RASGEF1A	664	-0,3773	838	-0,1618	597	-0,5288	3324	1	-0,1515	2118	1	0,2156	912	1	0,3671

MIR4435-1HG	565	0,4095	2217	0,1002	518	0,5607	3345	1	0,1512	1120	1	-0,3093	601	1	-0,4605
UPK3B	577	-0,4054	1958	-0,1072	531	-0,5563	3360	1	-0,1509	1184	1	0,2981	634	1	0,4490
ZFHX3	2455	0,1732	1619	0,1183	1415	0,3232	3390	1	0,1500	9141	1	-0,0548	2162	1	-0,2048
SULF2	121	0,7409	1784	0,1126	168	0,8898	3429	1	0,1489	244	1	-0,6284	181	1	-0,7773
ENDOD1	203	0,6397	3969	0,0667	692	0,4910	3437	1	-0,1488	316	1	-0,5731	705	1	-0,4243
HHIP	176	0,6707	20327	0,0033	207	0,8193	3444	1	0,1487	218	1	-0,6673	156	1	-0,8160
PALMD	4331	-0,1179	2518	-0,0925	1908	-0,2664	3449	1	-0,1486	13798	1	0,0254	2734	1	0,1739
SUSD2	162	-0,6882	539	-0,1944	198	-0,8360	3475	1	-0,1478	447	1	0,4938	305	1	0,6415
CACHD1	719	0,3604	8422	0,0275	648	0,5075	3496	1	0,1470	978	1	-0,3329	556	1	-0,4799
LIMCH1	9438	0,0507	318	0,2389	2943	0,1977	3498	1	0,1470	2628	1	0,1882	10798	1	0,0412
OSBPL6	7141	0,0737	3511	0,0738	2543	0,2203	3513	1	0,1466	29676	1	0,0001	3422	1	-0,1465
IGFL2	201	0,6411	9111	0,0240	227	0,7875	3520	1	0,1464	262	1	-0,6171	188	1	-0,7635
PNMAL1	1032	-0,2942	2484	-0,0933	854	-0,4403	3541	1	-0,1461	2379	1	0,2009	999	1	0,3469
FDXR	1382	-0,2495	393	-0,2194	1033	-0,3953	3550	1	-0,1458	12683	1	0,0302	2693	1	0,1760
SNAPC5	1409	-0,2472	2311	-0,0975	1045	-0,3928	3557	1	-0,1456	3625	1	0,1497	1288	1	0,2953
ZNF117	7286	0,0722	5382	0,0505	2575	0,2178	3562	1	0,1455	14735	1	-0,0218	2869	1	-0,1673
PKD1L2	6911	-0,0764	4062	-0,0654	2508	-0,2218	3565	1	-0,1454	19093	1	0,0109	3130	1	0,1564
GLIPR2	745	0,3545	1587	0,1201	666	0,4996	3581	1	0,1451	1832	1	-0,2344	848	1	-0,3795
PODXL	104	0,7666	2876	0,0846	158	0,9114	3597	1	0,1448	208	1	-0,6820	148	1	-0,8268
KCNAB2	2962	-0,1543	7938	-0,0302	1594	-0,2987	3605	1	-0,1445	4557	1	0,1240	1481	1	0,2685
CDH2	267	0,5733	941	0,1532	292	0,7176	3610	1	0,1443	632	1	-0,4200	407	1	-0,5643
NGF	3512	0,1380	10405	0,0187	1757	0,2823	3611	1	0,1443	4772	1	-0,1193	1528	1	-0,2636
PCDHAC2	1918	-0,2017	1657	-0,1171	1268	-0,3452	3639	1	-0,1436	6692	1	0,0846	1886	1	0,2282
MN1	641	0,3835	2121	0,1027	600	0,5271	3642	1	0,1435	1339	1	-0,2809	704	1	-0,4244
IER5	157	-0,6967	5520	-0,0492	537	-0,5537	3662	1	0,1429	226	1	0,6475	506	1	0,5046
DLC1	13389	0,0276	14009	0,0098	3633	0,1703	3671	1	0,1427	16181,5	1	-0,0178	3045	1	-0,1605
NIPSNAP1	2825	-0,1590	1812	-0,1116	1574	-0,3017	3674	1	-0,1427	9994	1	0,0474	2417	1	0,1901
NTF4	3463	0,1392	17269	0,0056	1779	0,2807	3710	1	0,1416	4193	1	-0,1336	1418	1	-0,2751
ILDR2	16230	0,0185	14016	0,0098	3933	0,1597	3721	1	0,1412	21653,5	1	-0,0087	3315	1	-0,1498
ITGB6	86	0,8014	3078	0,0810	141	0,9419	3747	1	0,1404	179	1	-0,7204	124	1	-0,8609
GRAMD4	5135	-0,1021	2119	-0,1027	2208	-0,2425	3751	1	-0,1404	29173	1	-0,0006	3641	1	0,1398
GRIN2B	11050	-0,0392	4071	-0,0653	3428	-0,1780	3811	1	-0,1389	13609	1	-0,0262	4767	1	0,1127
CFD	1528	-0,2346	1097	-0,1434	1123	-0,3734	3814	1	-0,1388	6274	1	0,0912	1867	1	0,2300

BMPR2	172	0,6799	4541	0,0595	209	0,8177	3846	1	0,1378	258	1	-0,6204	190	1	-0,7582
NOTCH3	25391	-0,0053	6017	-0,0447	4486	-0,1425	3866	1	-0,1372	11021	1	-0,0394	5549	1	0,0978
CCDC34	7301	-0,0720	1102	-0,1432	2739	-0,2090	3878	1	-0,1369	7663	1	-0,0712	7882	1	0,0658
MMD	5315	-0,0995	705	-0,1734	2290	-0,2365	3879	1	-0,1369	7437	1	-0,0738	8144	1	0,0631
MIR3167	9729	0,0484	8475	0,0273	3249	0,1844	3922	1	0,1360	14885	1	-0,0211	3110	1	-0,1572
ZPLD1	10183	0,0454	16826	0,0066	3349	0,1808	3953	1	0,1354	11130	1	-0,0388	2728	1	-0,1742
SEC24D	1478	0,2394	1062	0,1452	1115	0,3743	3972	1	0,1349	6073	1	-0,0942	1876	1	-0,2291
TENM3	336	0,5234	237	0,2659	360	0,6576	4005	1	0,1342	1567	1	-0,2576	796	1	-0,3917
CXCL16	2069	-0,1920	3240	-0,0780	1394	-0,3262	4008	1	-0,1341	5036	1	0,1141	1657	1	0,2482
ENGASE	5092	-0,1028	7467	-0,0333	2289	-0,2367	4018	1	-0,1339	7805	1	0,0695	2182	1	0,2034
BCAM	1293	-0,2586	8284	-0,0284	1046	-0,3924	4025	1	-0,1338	1891	1	0,2302	922	1	0,3640
CES3	6946	-0,0758	4118	-0,0646	2734	-0,2092	4045	1	-0,1334	19006	1	0,0111	3485	1	0,1446
FGF1	6881	0,0768	18587	0,0047	2727	0,2096	4071	1	0,1328	7565	1	-0,0721	2160	1	-0,2049
NR4A3	700	0,3655	14917	0,0080	2342	0,2329	4081	1	-0,1326	852	1	-0,3574	1925	1	-0,2249
LINC00152	246	0,5911	2385	0,0955	286	0,7235	4085	1	0,1325	442	1	-0,4955	316	1	-0,6280
GPR176	1662	0,2210	1352	0,1303	1225	0,3533	4093	1	0,1322	6299	1	-0,0907	1944	1	-0,2230
CCDC106	1924	-0,2014	1549	-0,1213	1342	-0,3335	4101	1	-0,1321	6967	1	0,0801	2060	1	0,2122
MALT1	2333	0,1789	3130	0,0801	1502	0,3108	4109	1	0,1320	5797	1	-0,0988	1856	1	-0,2308
C17orf89	1586	-0,2277	206	-0,2800	1198	-0,3594	4115	1	-0,1317	9432	1	-0,0523	6770	1	0,0795
TRPC6	468	-0,4460	448	-0,2092	487	-0,5776	4120	1	-0,1316	1806	1	0,2368	905	1	0,3684
CD83	5551	0,0958	3700	0,0708	2422	0,2265	4158	1	0,1307	13878	1	-0,0250	3145	1	-0,1557
HSPA1A	1584	-0,2281	11881	-0,0140	1202	-0,3588	4159	1	-0,1307	2148	1	0,2141	1012	1	0,3448
JAKMIP3	3129	-0,1492	6025	-0,0447	1786	-0,2798	4161	1	-0,1306	5476	1	0,1045	1801	1	0,2352
HECW2	1072	0,2896	4542	0,0595	922	0,4197	4191	1	0,1300	1893	1	-0,2302	944	1	-0,3602
TACC2	362	-0,5106	395	-0,2188	386	-0,6402	4204	1	-0,1296	1237	1	0,2918	718	1	0,4214
RP11-1069G10.2	1948	0,1998	26798	0,0007	1376	0,3292	4217	1	0,1294	2408	1	-0,1991	1092	1	-0,3285
EPB41L4B	695	-0,3676	8393	-0,0277	679	-0,4956	4274	1	-0,1280	935	1	0,3399	580	1	0,4679
RP11-820L6.1	16740	0,0176	2787	0,0862	4382	0,1455	4276	1	0,1279	7879	1	0,0686	8519	1	-0,0594
ZP3	3303	-0,1436	1919	-0,1088	1852	-0,2712	4301	1	-0,1275	11794	1	0,0348	2984	1	0,1624
AC005077.14	8180	-0,0624	6136	-0,0436	3128	-0,1897	4308	1	-0,1273	15638	1	0,0188	3429	1	0,1462
MAML2	1116	0,2816	3663	0,0713	984	0,4089	4312	1	0,1273	2220	1	-0,2103	1052	1	-0,3375
TFAP2C	320	-0,5330	775	-0,1660	356	-0,6603	4313	1	-0,1272	808	1	0,3671	524	1	0,4943
RNF43	963	-0,3058	1835	-0,1109	879	-0,4328	4322	1	-0,1270	2488	1	0,1950	1119	1	0,3219

STAT3	1833	-0,2080	227	-0,2715	1333	-0,3347	4336	1	-0,1267	8335	1	-0,0635	8132	1	0,0632
USP36	2180	-0,1869	1288	-0,1332	1480	-0,3132	4352	1	-0,1263	9295	1	0,0537	2602	1	0,1800
TTC39A	10236	-0,0449	11163	-0,0161	3603	-0,1711	4354	1	-0,1263	12932	1	0,0288	3164	1	0,1551
ALOX5AP	970	0,3047	8991	0,0246	888	0,4309	4357	1	0,1262	1348	1	-0,2802	756	1	-0,4064
MST1R	1277	-0,2605	3110	-0,0805	1057	-0,3867	4358	1	-0,1262	2807	1	0,1801	1217	1	0,3063
ALDH1A3	6127	-0,0871	4101	-0,0650	2675	-0,2121	4403	1	-0,1250	14686	1	0,0221	3399	1	0,1471
SERPINF2	13651	-0,0274	1705	-0,1154	4167	-0,1522	4415	1	-0,1248	6465	1	-0,0880	11457	1	0,0369
ZNF827	2462	0,1730	3103	0,0806	1622	0,2964	4474	1	0,1234	6199	1	-0,0924	2022	1	-0,2158
ELK3	177	0,6705	2243	0,0993	225	0,7938	4480	1	0,1233	318	1	-0,5712	254	1	-0,6945
RNFT2	665	-0,3766	6491	-0,0407	663	-0,4998	4482	1	-0,1233	956	1	0,3359	606	1	0,4592
RASA1	2667	0,1655	787	0,1653	1705	0,2880	4528	1	0,1224	29592	1	-0,0002	4291	1	-0,1227
STARD8	3459	-0,1393	3482	-0,0742	1964	-0,2614	4549	1	-0,1221	8189	1	0,0651	2465	1	0,1872
FOXD1	13115	0,0288	13581	0,0104	4217	0,1506	4559	1	0,1218	15959	1	-0,0185	3620	1	-0,1403
RAD51C	1523	-0,2352	881	-0,1585	1211	-0,3563	4592	1	-0,1211	7207	1	0,0767	2268	1	0,1978
OBFC1	1574	-0,2296	688	-0,1752	1235	-0,3507	4593	1	-0,1210	9199	1	0,0544	2703	1	0,1754
HEXIM1	3911	-0,1272	2579	-0,0910	2129	-0,2481	4598	1	-0,1209	11538	1	0,0363	3109	1	0,1572
MYH15	253	0,5842	8338	0,0280	769	0,4633	4600	1	-0,1209	345	1	-0,5561	669	1	-0,4353
ANKRD1	65	-0,8566	208	-0,2786	122	-0,9768	4631	1	-0,1201	307	1	0,5780	251	1	0,6982
PALM2	6545	0,0814	21034	0,0033	2873	0,2015	4632	1	0,1201	7114	1	-0,0781	2261	1	-0,1983
PPL	535	-0,4194	896	-0,1569	580	-0,5394	4636	1	-0,1201	1518	1	0,2624	833	1	0,3825
ROS1	4080	0,1235	10631	0,0178	2202	0,2427	4675	1	0,1192	5425	1	-0,1057	1923	1	-0,2249
ARMC7	4293	-0,1186	9543	-0,0221	2277	-0,2376	4686	1	-0,1190	5940	1	0,0965	2023	1	0,2155
HLA-B	2539	0,1702	1208	0,1378	1701	0,2885	4727	1	0,1183	12212	1	-0,0325	3301	1	-0,1508
UBE2O	3722	-0,1324	4683	-0,0577	2091	-0,2506	4736	1	-0,1183	7361	1	0,0747	2356	1	0,1930
SLCO4A1	654	-0,3803	9509	-0,0222	1956	-0,2622	4741	1	0,1181	849	1	0,3580	1738	1	0,2399
STARD13-AS	8342	0,0607	4705	0,0575	3416	0,1786	4752	1	0,1179	27867	1	-0,0032	4363	1	-0,1211
ITGB1	825	0,3390	862	0,1598	790	0,4569	4755	1	0,1179	2826	1	-0,1792	1275	1	-0,2970
MICAL2	333	0,5247	1666	0,1167	383	0,6424	4767	1	0,1177	665	1	-0,4079	469	1	-0,5256
STOM	325	-0,5295	144	-0,3274	377	-0,6470	4777	1	-0,1175	2355	1	0,2021	1134	1	0,3196
RGAG1	27400	0,0017	15955	0,0071	5483	0,1184	4817	1	0,1167	23904,5	1	0,0054	4837	1	-0,1113
SYNPO2	1366	-0,2507	2112	-0,1028	1164	-0,3670	4840	1	-0,1163	3687	1	0,1479	1523	1	0,2642
ALDH6A1	5361	-0,0989	7774	-0,0313	2620	-0,2149	4854	1	-0,1159	7964	1	0,0677	2537	1	0,1836
SEMA3B	6700	-0,0793	22339	-0,0025	3015	-0,1950	4873	1	-0,1156	7200	1	0,0768	2367	1	0,1924

PTPRH	4386	-0,1166	5343	-0,0508	2363	-0,2317	4897	1	-0,1151	8127	1	0,0658	2583	1	0,1809
C1QBP	1093	-0,2858	416	-0,2146	1008	-0,4008	4900	1	-0,1151	7670	1	0,0711	2485	1	0,1862
TOX2	1350	-0,2525	2582	-0,0909	1161	-0,3672	4914	1	-0,1147	3280	1	0,1616	1414	1	0,2763
FIBCD1	122	0,7394	12121	0,0132	188	0,8537	4941	1	0,1143	176	1	-0,7262	139	1	-0,8404
KCNE4	17240	0,0151	1415	0,1276	5013	0,1289	4965	1	0,1138	5108	1	0,1125	28546	1	-0,0013
FUT8	1533	0,2341	22171	0,0029	1251	0,3479	4966	1	0,1138	1878	1	-0,2313	1010	1	-0,3450
PKP3	365	-0,5093	10108	-0,0198	1029	-0,3957	4971	1	0,1136	457	1	0,4895	865	1	0,3759
FES	7232	-0,0727	2062	-0,1042	3215	-0,1860	4983	1	-0,1133	12404	1	-0,0315	6585	1	0,0818
GATS	1178	-0,2732	4195	-0,0635	1060	-0,3864	4988	1	-0,1132	2232	1	0,2097	1114	1	0,3229
RP6-99M1.3	26596	0,0030	6475	0,0408	5618	0,1155	5030	1	0,1124	11276	1	0,0378	7159	1	-0,0746
SCN9A	14020	0,0253	1538	0,1220	4673	0,1377	5031	1	0,1124	5918	1	0,0967	17065	1	-0,0157
KCNK5	3598	-0,1353	3454	-0,0747	2133	-0,2473	5056	1	-0,1120	8603	1	0,0606	2757	1	0,1726
KHK	1806	-0,2098	3049	-0,0815	1430	-0,3209	5093	1	-0,1111	4377	1	0,1283	1745	1	0,2394
CYB561	1554	-0,2320	8480	-0,0273	1287	-0,3424	5139	1	-0,1104	2319	1	0,2047	1155	1	0,3151
COL12A1	13989	0,0253	174	0,3042	4758	0,1354	5161	1	0,1101	1356	1	0,2788	2846	1	0,1688
IL1A	7320	0,0720	27131	0,0006	3331	0,1819	5166	1	0,1100	7645	1	-0,0713	2576	1	-0,1813
RP11-757F18.5	687	0,3700	14464	0,0090	736	0,4787	5241	1	0,1087	834	1	-0,3610	574	1	-0,4697
CITED2	1743	-0,2151	10378	-0,0188	1416	-0,3231	5278	1	-0,1080	2456	1	0,1964	1227	1	0,3043
GRID1	4755	0,1089	9974	0,0203	2594	0,2162	5299	1	0,1073	6419	1	-0,0886	2300	1	-0,1959
NLRP3	21054	0,0093	20990	0,0033	5584	0,1162	5319	1	0,1069	23538	1	-0,0060	4759,5	1	-0,1129
OTUB2	760	0,3513	978	0,1505	789	0,4570	5385	1	0,1058	2381	1	-0,2008	1213	1	-0,3066
SEPT10	543	0,4169	977	0,1506	609	0,5225	5399	1	0,1056	1477	1	-0,2663	885	1	-0,3719
HS3ST6	5846	-0,0913	4615	-0,0584	2985	-0,1958	5461	1	-0,1045	12128	1	0,0329	3726,5	1	0,1374
VPS37D	4961	-0,1053	7597	-0,0324	2725	-0,2097	5464	1	-0,1044	7507	1	0,0729	2670	1	0,1773
TRIO	892	0,3221	687	0,1753	900	0,4263	5471	1	0,1042	3734	1	-0,1468	1629	1	-0,2510
ANKLE2	959	0,3066	4412	0,0608	976	0,4101	5513	1	0,1035	1697	1	-0,2458	986	1	-0,3493
DHRS11	2439	-0,1738	1832	-0,1110	1807	-0,2771	5525	1	-0,1033	8395	1	0,0628	2899	1	0,1661
WBSCR27	8050	-0,0638	6573	-0,0400	3721	-0,1666	5557	1	-0,1028	14199	1	0,0237	4110	1	0,1266
DZIP1L	1553	0,2321	8180	0,0289	1328	0,3349	5565	1	0,1028	2341	1	-0,2032	1218	1	-0,3059
SLC16A9	4855	-0,1072	3499	-0,0741	2735	-0,2092	5604	1	-0,1020	12108	1	0,0331	3804	1	0,1351
TGFBR3	192	-0,6587	8302	-0,0283	257	-0,7606	5607	1	-0,1020	239	1	0,6304	220	1	0,7324
ZNF516	648	0,3820	1584	0,1202	717	0,4838	5618	1	0,1018	1523	1	-0,2617	924	1	-0,3636
OBSCN	2126	0,1895	884	0,1581	1674	0,2912	5627	1	0,1017	12422	1	-0,0314	3886	1	-0,1331

ACSM3	1184	-0,2715	2890	-0,0845	1134	-0,3718	5709	1	-0,1003	2657	1	0,1870	1335	1	0,2873
PARD6A	2017	-0,1946	2796	-0,0861	1641	-0,2947	5723	1	-0,1001	5288	1	0,1085	2109	1	0,2086
PADI3	2937	-0,1551	5438	-0,0500	2035	-0,2551	5730	1	-0,1000	5450	1	0,1051	2158	1	0,2051
ANK1	23	-1,1787	2683	-0,0884	47	-1,2781	5765	1	-0,0995	36	1	1,0902	39	1	1,1897
NT5C	1740	-0,2152	2607	-0,0903	1473	-0,3146	5774	1	-0,0994	4526	1	0,1249	1930	1	0,2243
AC007099.1	22139	0,0080	22050	0,0030	6055	0,1069	5802,5	1	0,0989	24191,5	1	-0,0050	5218	1	-0,1039
MAP2K3	973	-0,3041	2282	-0,0983	2794	-0,2058	5837	1	0,0983	2291	1	0,2058	5031	1	0,1075
AC131025.8	16118	0,0185	20473	0,0033	5583	0,1162	5878	1	0,0977	17141	1	-0,0152	4759,5	1	-0,1129
CMTM3	95	0,7867	933	0,1540	172	0,8843	5880	1	0,0977	237	1	-0,6327	222	1	-0,7304
RP11-82L18.2	10317	0,0443	7165	0,0353	4502	0,1419	5890	1	0,0976	21550	1	-0,0090	5070	1	-0,1066
ERV3-1	5866	0,0911	8507	0,0271	3179	0,1877	5943	1	0,0966	8284	1	-0,0640	3038	1	-0,1606
LASP1	807	-0,3422	7461	-0,0334	857	-0,4386	5954	1	-0,0964	1125	1	0,3088	757	1	0,4053
LUZP1	1456	0,2421	1154	0,1408	1303	0,3383	5964	1	0,0963	5668	1	-0,1013	2271	1	-0,1975
TYSND1	1305	-0,2574	3990	-0,0664	1224	-0,3534	5982	1	-0,0959	2569	1	0,1910	1336	1	0,2869
FOXA1	15796	-0,0197	520	-0,1972	5645	-0,1150	6041	1	-0,0952	2858	1	-0,1775	6547	1	-0,0822
MALL	511	-0,4283	9354	-0,0229	1345	-0,3333	6048	1	0,0951	678	1	0,4054	1195	1	0,3104
DCAKD	2498	-0,1718	497	-0,2008	1910	-0,2663	6081	1	-0,0945	12882	1	-0,0290	7906	1	0,0655
CDH4	248	0,5900	1537	0,1221	332	0,6843	6106	1	0,0943	506	1	-0,4679	414	1	-0,5622
ZIC5	865	0,3285	1541	0,1217	914	0,4223	6145	1	0,0937	2273	1	-0,2068	1255	1	-0,3005
ATXN1	408	0,4768	14867	0,0082	1073	0,3831	6146	1	-0,0937	502	1	-0,4686	872	1	-0,3749
PER1	1834	-0,2079	4070	-0,0653	1577	-0,3013	6161	1	-0,0934	3866	1	0,1425	1789	1	0,2359
RP11-438D8.2	7648	0,0683	12027	0,0136	3875	0,1613	6189	1	0,0930	9156	1	-0,0547	3371	1	-0,1477
AP000462.2	15835	0,0194	13382	0,0105	5783	0,1120	6210,5	1	0,0926	21552,5	1	-0,0090	5351	1	-0,1015
CNTNAP3	812	-0,3409	1037	-0,1463	876	-0,4332	6224	1	-0,0923	2498	1	0,1945	1338	1	0,2868
PLD6	2528	-0,1707	4821	-0,0562	1951	-0,2627	6240	1	-0,0920	5022	1	0,1145	2130	1	0,2066
RP11-160O5.1	3220	-0,1456	3443	-0,0748	2274	-0,2376	6241	1	-0,0920	7699	1	0,0708	2978	1	0,1628
TLN2	113	0,7502	1387	0,1288	196	0,8422	6248	1	0,0919	255	1	-0,6214	235	1	-0,7133
SOX13	4018	-0,1247	2829	-0,0855	2596	-0,2161	6286	1	-0,0914	11066	1	0,0391	3966	1	0,1306
FAM83H	2694	-0,1645	1660	-0,1170	2032	-0,2551	6338	1	-0,0907	9988	1	0,0475	3705	1	0,1381
PGD	749	-0,3537	9109	-0,0240	846	-0,4438	6376	1	-0,0902	993	1	0,3297	720	1	0,4198
NOXA1	2786	-0,1609	5949	-0,0452	2085	-0,2511	6380	1	-0,0901	4939	1	0,1158	2146	1	0,2059
RBFOX3	9965	-0,0468	4854	-0,0559	4717	-0,1367	6402	1	-0,0899	21544	1	-0,0090	6655	1	0,0809
GATA5	17288	-0,0150	7869	-0,0307	6193	-0,1045	6429	1	-0,0895	16986	1	-0,0157	7213	1	0,0738

ZDHHC8P1	4724	-0,1097	3337	-0,0763	2925	-0,1985	6472	1	-0,0888	12029	1	0,0334	4314	1	0,1222
NKX6-1	3194	0,1463	12270	0,0131	2313	0,2351	6478	1	0,0888	4201	1	-0,1333	1953	1	-0,2221
RASSF6	8555	-0,0585	5321	-0,0510	4321	-0,1472	6481,5	1	-0,0888	22146	1	0,0074	5647	1	0,0962
DMBX1	1446	0,2431	7395	0,0338	1356	0,3319	6485	1	0,0887	2242	1	-0,2093	1270	1	-0,2981
IRF2BP2	1467	-0,2409	4236	-0,0629	1374	-0,3296	6487	1	-0,0887	2854	1	0,1779	1500	1	0,2666
DCBLD1	268	0,5720	2865	0,0848	714	0,4840	6547	1	-0,0880	464	1	-0,4872	774	1	-0,3992
NR1D2	557	0,4119	640	0,1814	665	0,4997	6569	1	0,0877	1889	1	-0,2305	1142	1	-0,3183
TGFB2	899	0,3201	390	0,2198	2326	0,2344	6716	1	-0,0856	5718	1	-0,1002	17485	1	-0,0146
SELENBP1	16616	-0,0183	7174	-0,0352	6284	-0,1032	6776	1	-0,0849	16516	1	-0,0169	7702	1	0,0679
NOTCH2	2392	0,1765	5862	0,0459	1969	0,2610	6796	1	0,0845	4293	1	-0,1305	2029	1	-0,2150
ST6GALNAC5	27845	0,0017	12073	0,0136	7420	0,0852	6880,5	1	0,0835	18802	1	0,0119	7380,5	1	-0,0716
UGDH	890	-0,3224	3486	-0,0742	995	-0,4052	6943	1	-0,0827	1666	1	0,2483	1077	1	0,3310
MTAP	7423	0,0707	18301	0,0051	4154	0,1525	7018	1	0,0818	8149	1	-0,0655	3383	1	-0,1474
FLYWCH2	834	-0,3369	3125	-0,0802	932	-0,4187	7025	1	-0,0818	1580	1	0,2568	1049	1	0,3385
SLAIN1	9777	-0,0482	5763	-0,0469	4997	-0,1293	7090	1	-0,0810	28673	1	0,0013	6542	1	0,0824
A4GALT	9833	-0,0477	6862	-0,0377	5028	-0,1286	7098	1	-0,0809	19648	1	0,0100	5985	1	0,0909
IGFBP1	7267	-0,0724	13692	-0,0101	4136	-0,1531	7120	1	-0,0807	8449	1	0,0623	3528	1	0,1430
DAGLA	2632	-0,1667	8587	-0,0267	2158	-0,2458	7242	1	-0,0791	3966	1	0,1400	1989	1	0,2191
RHPN2	25396	-0,0052	1335	-0,1307	7588	-0,0830	7351	1	-0,0778	4495	1	-0,1256	9887	1	-0,0477
COPRS	5346	-0,0991	545	-0,1932	3463	-0,1766	7377	1	-0,0775	6087	1	-0,0940	16728	1	-0,0166
AC005355.2	3963	-0,1259	9518	-0,0222	2836	-0,2030	7409	1	-0,0771	5523	1	0,1038	2585	1	0,1809
GUCY1A2	4213	-0,1202	3588	-0,0725	2967	-0,1968	7453	1	-0,0766	9955	1	0,0477	4213,5	1	0,1243
TPPP3	8570	-0,0583	8220	-0,0287	4799	-0,1344	7499,5	1	-0,0761	12787	1	0,0296	5112	1	0,1057
IFIH1	8564	-0,0583	7690	-0,0318	4796	-0,1344	7499,5	1	-0,0761	13511	1	0,0265	5285	1	0,1026
CA12	210	-0,6346	8938	-0,0249	300	-0,7102	7537	1	-0,0756	270	1	0,6098	264	1	0,6853
SULT2B1	5090	-0,1029	5143	-0,0527	3429	-0,1780	7556	1	-0,0752	9694	1	0,0501	4162	1	0,1253
PCDH10	5985	0,0895	25440	0,0018	3794	0,1643	7580	1	0,0748	6484	1	-0,0877	2983	1	-0,1625
FAM195A	558	-0,4118	14482	-0,0089	1311	-0,3373	7605	1	0,0746	689	1	0,4029	1093	1	0,3284
ADAM11	10290	-0,0445	11746	-0,0144	5452	-0,1190	7606	1	-0,0745	12688	1	0,0301	5177	1	0,1047
ZNF525	9741	0,0484	4321	0,0618	5278	0,1229	7610,5	1	0,0745	17908	1	0,0134	8341	1	-0,0611
PCDHAC1	5349	-0,0991	6814	-0,0380	3606	-0,1711	7805,5	1	-0,0720	8554,5	1	0,0611	3887,5	1	0,1331
MDGA1	307	0,5478	2801	0,0860	738	0,4759	7820	1	-0,0719	525	1	-0,4618	804	1	-0,3899
WRAP53	3725	-0,1324	321	-0,2382	2819	-0,2039	7841	1	-0,0715	5420	1	-0,1059	11909	1	-0,0343

TRAF4	3318	-0,1433	3864	-0,0683	2639	-0,2141	7907	1	-0,0708	7337	1	0,0749	3449	1	0,1457
PPM1B	1214	-0,2683	3287	-0,0771	1301	-0,3388	7917	1	-0,0705	2561	1	0,1912	1538	1	0,2617
AIFM3	11734	-0,0358	9300	-0,0231	6107	-0,1058	7969,5	1	-0,0700	18529	1	0,0127	6509	1	0,0827
SPRY1	10754	-0,0413	11277	-0,0156	5906	-0,1095	8126	1	-0,0682	13728	1	0,0257	5803	1	0,0939
GPR37	9070	-0,0541	5879	-0,0458	5319	-0,1221	8145	1	-0,0680	21950	1	0,0083	7024,5	1	0,0762
NCKAP5	14948	0,0220	228	0,2703	7117	0,0893	8217	1	0,0673	1662	1	0,2484	2579	1	0,1811
LIMD1	394	-0,4853	11034	-0,0163	936	-0,4181	8229	1	0,0672	499	1	0,4690	768	1	0,4018
AGMO	2616	0,1675	9025	0,0244	6417	0,1008	8278	1	-0,0667	3849	1	-0,1431	7012	1	-0,0764
C4orf26	4442	0,1156	15906	0,0071	3330	0,1819	8312	1	0,0664	5291	1	-0,1084	2717	1	-0,1748
CTD-2560E9.5	13385	0,0276	16471	0,0066	6844	0,0938	8323,5	1	0,0662	14918,5	1	-0,0210	6205,5	1	-0,0873
CACNG7	5330	-0,0993	5022	-0,0542	3762	-0,1653	8346	1	-0,0660	10287	1	0,0451	4842	1	0,1111
PYGL	209	0,6353	7443	0,0335	504	0,5699	8385	1	-0,0654	276	1	-0,6018	452	1	-0,5364
FRYL	2713	0,1637	5282	0,0514	2390	0,2291	8388	1	0,0653	5111	1	-0,1123	2664	1	-0,1777
CRIP1	28269	-0,0002	6567	-0,0401	8956	-0,0654	8393	1	-0,0653	10954	1	-0,0399	13795	1	0,0254
NPTX1	1	1,7606	1143	0,1411	15	1,6954	8398	1	-0,0652	8	1	-1,6195	11	1	-1,5543
HLA-DMB	3339	-0,1427	3807	-0,0694	2754	-0,2079	8399	1	-0,0652	7462	1	0,0734	3689	1	0,1386
RP11-807H7.1	16468	0,0185	9391	0,0227	7618	0,0825	8493	1	0,0640	25814,5	1	0,0042	8471	1	-0,0598
RP11-131L23.1	19023	0,0123	10787	0,0173	8105	0,0758	8542	1	0,0635	24203	1	0,0050	8609	1	-0,0585
TSEN54	1131	-0,2792	856	-0,1603	1288	-0,3423	8594	1	-0,0630	4785	1	0,1190	2567	1	0,1820
VLDLR	1117	0,2815	1614	0,1186	1280	0,3440	8647	1	0,0625	3237	1	-0,1629	1916	1	-0,2254
MIR22HG	276	0,5645	5056	0,0537	655	0,5026	8710	1	-0,0619	415	1	-0,5108	635	1	-0,4489
PPP1R14C	588	0,4008	11224	0,0157	772	0,4627	8719	1	0,0619	745	1	-0,3851	642	1	-0,4470
DNAAF3	5775	-0,0923	5551	-0,0488	4123	-0,1536	8793	1	-0,0613	10472	1	0,0435	5171	1	0,1047
NPTXR	388	0,4873	4609	0,0585	556	0,5483	8829	1	0,0609	609	1	-0,4289	538	1	-0,4898
SEMA3C	217	0,6268	934	0,1537	328	0,6874	8853	1	0,0606	491	1	-0,4731	459	1	-0,5337
MLXIPL	14372	-0,0234	8386	-0,0277	7534	-0,0837	8903	1	-0,0602	25804	1	-0,0043	8893	1	0,0560
TAS2R14	6941	0,0758	6399	0,0414	17882	0,0156	8907	1	-0,0602	11840	1	-0,0344	13633	1	0,0258
ANGPT1	10450	-0,0432	6768	-0,0384	6278	-0,1032	8943,5	1	-0,0599	24224,5	1	0,0049	7975,5	1	0,0648
SPTBN4	14437	-0,0231	7382	-0,0339	7649	-0,0822	9040	1	-0,0591	19124	1	-0,0108	9809	1	0,0483
PTPRE	581	0,4041	1263	0,1343	1271	0,3451	9049	1	-0,0591	1446	1	-0,2698	2079	1	-0,2107
IFI35	4189	-0,1207	6869	-0,0377	3387	-0,1797	9064	1	-0,0590	6774	1	0,0831	3565	1	0,1420
PER2	284	-0,5595	573	-0,1891	659	-0,5007	9082	1	0,0588	794	1	0,3704	1182	1	0,3116
RP11-265N7.1	11326	0,0377	15988	0,0071	6690	0,0964	9085	1	0,0587	12602,5	1	-0,0305	6075	1	-0,0893

SNX8	1551	-0,2322	886	-0,1579	1680	-0,2906	9100	1	-0,0585	7402	1	0,0743	3891	1	0,1328
ANXA13	4246	-0,1196	4853	-0,0559	3427	-0,1780	9102	1	-0,0584	8315	1	0,0637	4317	1	0,1222
LRP1	578	0,4052	4566	0,0591	1256	0,3470	9126	1	-0,0583	904	1	-0,3461	1330	1	-0,2879
RP11-1002K11.1	1147	0,2766	1260	0,1345	1334	0,3347	9138	1	0,0581	3885	1	-0,1422	2230	1	-0,2003
RNF144B	2863	-0,1575	4988	-0,0545	2612	-0,2153	9162	1	-0,0578	5573	1	0,1030	3028	1	0,1608
ELMO1	27931	-0,0015	8739	-0,0258	9562	-0,0591	9185,5	1	-0,0576	14004	1	-0,0244	12111	1	0,0332
NRG1	1420	0,2464	4476	0,0601	1560	0,3039	9190	1	0,0575	2672	1	-0,1863	1703	1	-0,2438
PM20D2	983	-0,3034	5688	-0,0476	1194	-0,3600	9273	1	-0,0566	1591	1	0,2557	1176	1	0,3123
BEAN1	111	0,7508	4485	0,0600	216	0,8060	9422	1	0,0553	198	1	-0,6908	204	1	-0,7461
TUBG2	2702	-0,1641	766	-0,1668	2557	-0,2193	9429	1	-0,0552	28169	1	-0,0026	9278	1	0,0526
GCHFR	2277	-0,1818	4736	-0,0572	2303	-0,2357	9586	1	-0,0539	4540	1	0,1246	2642	1	0,1785
CDC42BPG	3146	-0,1485	4987	-0,0545	2862	-0,2019	9646	1	-0,0534	6094	1	0,0940	3382	1	0,1474
GOLT1A	4905	-0,1063	5301	-0,0512	3948	-0,1593	9682	1	-0,0530	9123	1	0,0550	4995	1	0,1081
SLC25A10	298	-0,5504	1506	-0,1234	673	-0,4977	9714	1	0,0528	616	1	0,4270	875	1	0,3742
MSC	575	0,4067	3280	0,0772	786	0,4584	9885	1	0,0516	995	1	-0,3295	843	1	-0,3812
TSPAN5	709	0,3626	5472	0,0497	1498	0,3111	9902	1	-0,0515	1090	1	-0,3129	1544	1	-0,2614
LRP5	455	-0,4523	2852	-0,0851	1006	-0,4014	9953	1	0,0509	807	1	0,3672	1152	1	0,3163
WISP2	5561	-0,0957	6595	-0,0399	4350	-0,1466	9967	1	-0,0508	9041	1	0,0558	5068	1	0,1067
TNFRSF9	26548	0,0033	564	0,1907	10103	0,0541	9978	1	0,0508	2646	1	0,1873	3753	1	0,1366
REEP1	3702	0,1328	9749	0,0212	3290	0,1831	10050	1	0,0503	5142	1	-0,1116	2998	1	-0,1619
EPB41L1	1108	-0,2825	7490	-0,0332	1348	-0,3327	10062	1	-0,0502	1653	1	0,2493	1262	1	0,2995
ADAMTS9-AS1	12292	0,0325	10480	0,0183	7666	0,0819	10163	1	0,0494	17640,5	1	-0,0142	8085	1	-0,0636
RP1-74M1.3	227	0,6154	2329	0,0971	351	0,6640	10265	1	0,0486	402	1	-0,5182	403	1	-0,5669
SNAI1	282	0,5615	7228	0,0349	428	0,6100	10288	1	0,0485	387	1	-0,5266	385	1	-0,5751
DUSP10	48	0,9566	1604	0,1191	113	1,0050	10306	1	0,0483	106	1	-0,8376	115	1	-0,8859
CYP4F12	3336	-0,1428	4702	-0,0575	3109	-0,1906	10370	1	-0,0478	6644	1	0,0853	3885	1	0,1331
PREX1	997	-0,3011	3562	-0,0729	1248	-0,3482	10470	1	-0,0471	1922	1	0,2282	1417	1	0,2753
PRODH	1458	-0,2418	3903	-0,0677	1696	-0,2889	10479	1	-0,0470	2933	1	0,1742	1967	1	0,2212
SLC22A4	2030	0,1939	2947	0,0834	2239	0,2405	10529	1	0,0466	5193	1	-0,1105	3112	1	-0,1571
ACTBL2	7315	0,0720	26917	0,0006	5482	0,1184	10553	1	0,0464	7645	1	-0,0713	4500	1	-0,1178
U8	12680	0,0302	4023	0,0659	8051	0,0765	10589	1	0,0462	11632	1	0,0356	20084	1	-0,0106
AC096677.1	886	0,3230	10499	0,0183	1152	0,3692	10593	1	0,0462	1143	1	-0,3048	983	1	-0,3510
EPHA10	2838	-0,1585	7707	-0,0317	2830	-0,2033	10768	1	-0,0448	4446	1	0,1268	2782	1	0,1716

TGFA	790	-0,3440	6355	-0,0417	1052	-0,3888	10770	1	-0,0448	1151	1	0,3023	998	1	0,3471
GLIPR1	222	0,6208	637	0,1815	350	0,6656	10776	1	0,0447	583	1	-0,4394	553	1	-0,4841
PHLDB1	26	1,1293	6969	0,0368	59	1,1739	10793	1	0,0446	35	1	-1,0925	47	1	-1,1371
CD14	1099	-0,2844	1349	-0,1304	1378	-0,3289	10810	1	-0,0445	3510	1	0,1540	2255	1	0,1985
TMOD2	666	0,3765	6429	0,0411	925	0,4195	11018	1	0,0431	960	1	-0,3353	853	1	-0,3784
CUTC	835	-0,3368	1622	-0,1182	1090	-0,3798	11023	1	-0,0430	2064	1	0,2186	1540	1	0,2616
ANKRD30A	7188	-0,0732	6156	-0,0434	5604	-0,1158	11077	1	-0,0427	12770	1	0,0298	7314	1	0,0725
CYP4F3	1431	-0,2450	3348	-0,0761	1721	-0,2861	11319	1	-0,0411	3093	1	0,1688	2089	1	0,2100
HMCN1	6525	0,0817	437	0,2109	5297	0,1225	11355	1	0,0409	4345	1	0,1292	6137	1	0,0883
CTNNAL1	105	-0,7644	7037	-0,0362	285	-0,7237	11374	1	0,0407	172	1	0,7282	260	1	0,6875
DNAJB4	371	0,5008	9110	0,0240	775	0,4612	11524	1	-0,0396	483	1	-0,4769	667	1	-0,4372
CBLN2	3266	0,1444	17189	0,0057	3268	0,1839	11547	1	0,0395	4007	1	-0,1387	2651	1	-0,1782
INHBB	353	-0,5151	1135	-0,1415	538	-0,5536	11683	1	-0,0385	784	1	0,3736	740	1	0,4122
RP11-53B2.2	1880	0,2047	16711	0,0066	2200	0,2428	11756	1	0,0380	2426	1	-0,1982	1785	1	-0,2362
FMN2	1623	-0,2243	7701	-0,0318	1966	-0,2613	11946	1	-0,0370	2536	1	0,1925	1873	1	0,2295
HIP1	538	-0,4186	932	-0,1541	797	-0,4554	11969	1	-0,0368	1501	1	0,2646	1245	1	0,3014
SNORD112	3765	0,1312	12463	0,0125	6764	0,0950	12060	1	-0,0362	4797	1	-0,1187	6526	1	-0,0825
ACOX2	2110	-0,1901	6275	-0,0424	2435	-0,2258	12138	1	-0,0357	3695	1	0,1477	2541	1	0,1834
G0S2	1039	-0,2931	3886	-0,0679	2004	-0,2575	12188	1	0,0356	1969	1	0,2252	2428	1	0,1896
LTBP3	136	0,7183	2635	0,0896	264	0,7535	12322	1	0,0352	242	1	-0,6286	282	1	-0,6638
S100P	17374	-0,0150	12819	-0,0118	10602	-0,0500	12380	1	-0,0350	27857,5	1	0,0032	11242,5	1	0,0382
NFASC	2418	0,1749	11801	0,0141	2728	0,2096	12471	1	0,0347	3305	1	-0,1607	2308	1	-0,1955
SPTLC2	978	0,3038	11141	0,0162	1304	0,3382	12533	1	0,0344	1277	1	-0,2876	1117	1	-0,3221
KCTD12	8376	-0,0604	7577	-0,0325	6791	-0,0947	12588	1	-0,0343	13135	1	0,0279	8241	1	0,0622
TTLL6	8381	-0,0604	6320	-0,0420	6794	-0,0947	12588	1	-0,0343	15995	1	0,0184	9256	1	0,0527
AQP3	3548	-0,1369	4228	-0,0630	3622	-0,1708	12686	1	-0,0339	7429	1	0,0739	5009	1	0,1078
TES	426	0,4707	7364	0,0340	656	0,5024	13103	1	0,0317	589	1	-0,4367	578	1	-0,4684
TRERF1	674	-0,3741	3494	-0,0741	1286	-0,3427	13136	1	0,0314	1168	1	0,3000	1476	1	0,2686
TSPAN1	1229	-0,2661	3254	-0,0776	1608	-0,2974	13137	1	-0,0313	2623	1	0,1885	1980	1	0,2198
RP11-1055B8.7	921	-0,3156	28099	0,0000	1740	-0,2843	13142	1	0,0313	1070	1	0,3155	1360	1	0,2842
C5	699	-0,3658	1002	-0,1487	1024	-0,3968	13171	1	-0,0309	2091	1	0,2171	1660	1	0,2481
SH2D5	685	-0,3718	10081	-0,0199	1003	-0,4023	13230	1	-0,0305	877	1	0,3518	836	1	0,3824
AREG	1813	-0,2095	9037	-0,0244	2252	-0,2395	13356	1	-0,0300	2686	1	0,1851	2028	1	0,2151

RP11-392B6.1	5925	0,0903	7729	0,0316	5404	0,1202	13380	1	0,0299	8772	1	-0,0587	6120	1	-0,0886
TJP3	2073	-0,1918	3590	-0,0725	2519	-0,2214	13424	1	-0,0296	4771	1	0,1193	3343,5	1	0,1489
HSPA1B	2229	-0,1842	18993	-0,0045	2646	-0,2138	13434	1	-0,0296	2816	1	0,1797	2096	1	0,2093
AIF1L	5826	-0,0916	3855	-0,0686	9258	-0,0621	13452	1	0,0295	14367	1	0,0230	23038	1	-0,0064
RP11-36N20.1	5951	0,0900	22462	0,0024	9407	0,0608	13494	1	-0,0292	6486	1	-0,0876	8619,5	1	-0,0584
STXBP5-AS1	4838	0,1073	12887	0,0115	4728	0,1364	13514	1	0,0291	5991	1	-0,0958	4187,5	1	-0,1248
EZR	312	-0,5420	2434	-0,0945	502	-0,5705	13601	1	-0,0285	552	1	0,4475	561	1	0,4760
KRT15	4730	-0,1096	5129	-0,0529	4665	-0,1379	13620	1	-0,0284	8954	1	0,0567	6345	1	0,0851
HTRA1	229	0,6108	9430	0,0226	387	0,6390	13630	1	0,0283	292	1	-0,5882	326	1	-0,6165
RP11-170N16.3	4025	0,1245	14149	0,0097	4166	0,1523	13763	1	0,0278	4997	1	-0,1149	3546	1	-0,1426
SPRY3	4067	0,1238	7086	0,0358	4218	0,1506	14050	1	0,0268	6463	1	-0,0880	4661	1	-0,1148
RASSF7	1232	-0,2658	1416	-0,1276	1661	-0,2925	14062	1	-0,0267	4030	1	0,1382	2932	1	0,1649
METRNL	631	0,3872	6797	0,0382	957	0,4136	14121	1	0,0264	893	1	-0,3490	867	1	-0,3754
PHLDB2	767	0,3490	5294	0,0513	1418	0,3226	14154	1	-0,0263	1189	1	-0,2977	1452	1	-0,2713
LPHN3	20940	0,0093	8149	0,0291	12600	0,0356	14176	1	0,0263	15271,5	1	0,0198	22986	1	-0,0065
CTD-2066L21.1	1250	0,2637	22408	0,0024	1689	0,2898	14263	1	0,0261	1527	1	-0,2613	1332	1	-0,2874
ST6GALNAC3	17029	0,0165	4960	0,0548	11591	0,0425	14296	1	0,0260	11200	1	0,0383	18742	1	0,0123
UNC13A	5410	-0,0980	19226	-0,0044	5245	-0,1238	14506	1	-0,0258	6111	1	0,0937	4431	1	0,1195
SLC25A45	5283	-0,1001	8495	-0,0271	5146	-0,1258	14511,5	1	-0,0257	7504	1	0,0729	5489	1	0,0987
MB	2540	-0,1702	4616	-0,0584	2986	-0,1958	14520	1	-0,0256	5130	1	0,1118	3726,5	1	0,1374
SLC22A3	984	0,3032	9171	0,0237	1802	0,2777	14535	1	-0,0254	1350	1	-0,2795	1599	1	-0,2541
CD151	379	0,4945	4156	0,0641	619	0,5197	14632	1	0,0252	607	1	-0,4304	621	1	-0,4556
RHOV	1156	-0,2757	13138	-0,0110	1589	-0,2998	14813	1	-0,0241	1499	1	0,2647	1321	1	0,2888
RP11-70J12.1	7787	0,0668	7769	0,0313	11552	0,0427	14828	1	-0,0240	11671	1	-0,0354	19808	1	-0,0114
AC006262.4	124	0,7346	20334	0,0033	260	0,7582	15182	1	0,0236	168	1	-0,7313	197	1	-0,7549
PVR	1016	0,2983	985	0,1502	1425	0,3217	15365	1	0,0234	3675	1	-0,1481	2784	1	-0,1715
AKAP12	328	0,5279	258	0,2583	546	0,5504	15565	1	0,0225	1447	1	-0,2696	1302	1	-0,2921
C17orf96	3475	-0,1388	608	-0,1838	3880	-0,1612	15576	1	-0,0224	10307	1	-0,0450	14605	1	-0,0226
FAM84B	1150	-0,2765	12133	-0,0132	1593	-0,2987	15594	1	-0,0222	1512	1	0,2634	1347	1	0,2856
FGFBP1	477	-0,4436	4953	-0,0549	761	-0,4657	15602	1	-0,0221	736	1	0,3887	746	1	0,4108
MMP24	161	-0,6884	6258	-0,0426	301	-0,7101	15651	1	-0,0217	228	1	0,6459	278	1	0,6676
SLCO1B1	7056	0,0747	2060	0,1042	6700	0,0963	15680	1	0,0216	12801	1	0,0295	21915	1	0,0079
CDC42EP4	694	-0,3676	4429	-0,0607	1253	-0,3474	15992	1	0,0202	1130	1	0,3070	1339	1	0,2867

CNTNAP3B	815	-0,3407	177	-0,3027	1432	-0,3207	16019	1	0,0200	11264	1	0,0379	16230	1	0,0179
ANKRD2	2505	-0,1714	9910	-0,0206	3102	-0,1909	16084	1	-0,0195	3593	1	0,1509	2817	1	0,1703
PPAPDC1A	3406	0,1408	16572	0,0066	3934	0,1597	16242	1	0,0189	4165	1	-0,1342	3228	1	-0,1531
RP11-292F22.7	1532	0,2343	16958	0,0062	2603	0,2158	16301	1	-0,0185	1925	1	-0,2281	2092	1	-0,2096
PLCD3	498	-0,4354	7822	-0,0310	808	-0,4530	17076	1	-0,0176	684	1	0,4045	716	1	0,4220
BCL2L15	9135	-0,0534	16156	-0,0068	8541	-0,0704	17238	1	-0,0170	10074	1	0,0466	8079,5	1	0,0636
LIN28B	7765	-0,0668	7837	-0,0309	7533	-0,0837	17366,5	1	-0,0169	11593,5	1	0,0360	9244,5	1	0,0528
EXOC3L4	6170	-0,0865	13736	-0,0100	6280	-0,1032	17448	1	-0,0166	7218	1	0,0765	5855	1	0,0932
EDIL3	9056	0,0541	1602	0,1192	8616	0,0696	17932	1	0,0155	8187	1	0,0651	9659	1	0,0497
PDLIM5	644	-0,3826	53	-0,4625	1159	-0,3677	18211	1	0,0149	6991	1	-0,0798	5742	1	-0,0947
MGAM	6341	-0,0841	6468	-0,0409	6577	-0,0982	18758,5	1	-0,0141	10495	1	0,0432	8744	1	0,0573
DSE	243	0,5933	3142	0,0798	485	0,5792	18762	1	-0,0141	408	1	-0,5134	516	1	-0,4993
RNF135	5232	-0,1009	723	-0,1717	5653	-0,1148	18776	1	-0,0139	7695	1	-0,0709	8787	1	-0,0569
SLC12A2	370	-0,5019	29	-0,5243	629	-0,5158	18784	1	-0,0139	14519	1	-0,0224	21774	1	-0,0085
AKR1B10P1	729	-0,3589	11219	-0,0157	1127	-0,3728	18787	1	-0,0139	919	1	0,3432	957	1	0,3571
SGSM1	3673	-0,1335	5656	-0,0479	4322	-0,1472	18803	1	-0,0137	6631	1	0,0856	5464	1	0,0993
BCL6	4173	-0,1211	1734	-0,1145	4785	-0,1347	18819	1	-0,0136	22954	1	0,0067	15425	1	0,0203
PLAUR	231	0,6087	7089	0,0358	412	0,6222	18836	1	0,0135	317	1	-0,5729	365	1	-0,5864
ZNF467	2153	-0,1880	9406	-0,0227	2888	-0,2009	18907	1	-0,0129	3181	1	0,1653	2648	1	0,1782
PAPLN	4982	-0,1048	28050	-0,0002	5522	-0,1176	19006	1	-0,0127	5470	1	0,1047	4521	1	0,1174
GDA	3429	-0,1401	4645	-0,0581	4152	-0,1525	19142	1	-0,0124	6854	1	0,0820	5763	1	0,0945
TERT	1912	-0,2021	5008	-0,0543	3131	-0,1897	19144	1	0,0124	3690	1	0,1478	3797	1	0,1354
GCLC	636	-0,3863	771	-0,1664	1118	-0,3740	19202	1	0,0123	2044	1	0,2199	2117	1	0,2076
RP11-114F10.3	2640	0,1664	28066	0,0001	3447	0,1774	20909	1	0,0109	3154	1	-0,1663	2671	1	-0,1772
RP11-170N16.2	8385	0,0604	12024	0,0136	8536	0,0704	21109	1	0,0100	10060	1	-0,0468	8796,5	1	-0,0568
LIMK2	572	0,4075	7613	0,0323	939	0,4172	21213	1	0,0097	777	1	-0,3752	829	1	-0,3849
KCNF1	1031	-0,2945	13137	-0,0110	1710	-0,2874	23457	1	0,0071	1311	1	0,2835	1412	1	0,2764
CSF2RA	675	-0,3740	13101	-0,0111	1087	-0,3805	24139	1	-0,0065	828	1	0,3629	898	1	0,3694
NR2F6	1136	-0,2784	1126	-0,1420	1844	-0,2721	24473	1	0,0063	4086	1	0,1364	3980	1	0,1301
NEDD4L	517	-0,4260	2501	-0,0929	884	-0,4318	24681	1	-0,0058	976	1	0,3331	1044	1	0,3388
SPDEF	194	-0,6531	285	-0,2472	374	-0,6494	25346	1	0,0038	673	1	0,4059	766	1	0,4021
FRMD3	3310	-0,1435	1399	-0,1280	4547	-0,1408	26398	1	0,0027	17027	1	0,0155	18257	1	0,0127
RP11-361F15.2	1498	0,2375	13249	0,0107	2284	0,2372	28764	1	-0,0003	1946	1	-0,2268	1908	1	-0,2265

MBOAT1	9416	-0,0510	3422	-0,0751	10423	-0,0512	29357	1	-0,0002	14116	1	-0,0240	14293	1	-0,0238

[1] order of identified genes

[2] indicates agreement or difference in expression change relative to A549scr
