# Supplementary material for: Identification of AGR2 Gene-Specific Expression Patterns Associated with Epithelial-Mesenchymal Transition
Source: Int J Mol Sci. 2022 Sep 16;23(18):10845. doi: 10.3390/ijms231810845 (PMC9504245; doi:10.3390/ijms231810845)
Supplement: Supplementary file 1 [file ijms-23-10845-s001.zip › Table S2 - analysis summary.pdf]

Table S2. Summary of all identified pathway results with analysis conditions depicted in Figure 2, their corresponding number of genes, and P-level

Experimental definition	Kegg pathways	p value	Number of genes
A: scr TGFβ vs scr ; 0.67 > FC > 1.5; p < 0.05	Focal adhesion	7,50E-10*	33
	Proteoglycans in cancer	2,60E-07*	29
	ECM-receptor interaction	4,90E-07*	18
	AGE-RAGE signaling pathway in diabetic complications	6,80E-07*	19
	Pathways in cancer	1,20E-06*	51
	Small cell lung cancer	4,50E-06*	17
	PI3K-Akt signaling pathway	7,60E-06*	37
	Human papillomavirus infection	1,10E-05*	35
	Hippo signaling pathway	1,20E-05*	22
	Axon guidance	1,20E-05*	24
	MAPK signaling pathway	1,70E-05*	32
	Rap1 signaling pathway	4,20E-05*	25
	Regulation of actin cytoskeleton	7,70E-05*	25
	Arrhythmogenic right ventricular cardiomyopathy	2,10E-04*	13
	Transcriptional misregulation in cancer	6,20E-04*	21
	Amoebiasis	8,80E-04*	14
	Hypertrophic cardiomyopathy	9,20E-04*	13
	EGFR tyrosine kinase inhibitor resistance	1,00E-03*	12
	TGF-beta signaling pathway (KEGG)	1,40E-03*	13
	Glycosaminoglycan biosynthesis - chondroitin sulfate / dermatan sulfate	1,90E-03*	6
	Fluid shear stress and atherosclerosis	2,00E-03*	16
	TNF signaling pathway	2,10E-03*	14
	Tight junction	2,30E-03*	18
	Pancreatic cancer	2,70E-03*	11
	Relaxin signaling pathway	2,70E-03*	15

	Dilated cardiomyopathy	5,00E-03	12
	MicroRNAs in cancer	5,30E-03	26
	Melanoma	6,10E-03	10
	TGF beta signaling pathway (Biocarta)	6,80E-03	6
	Gastric cancer	9,70E-03	15
	Lipid and atherosclerosis	1,20E-02	19
	Platelet Amyloid Precursor Protein Pathway	1,50E-02	5
	Hematopoietic cell lineage	1,70E-02	11
	Calcium signaling pathway	1,70E-02	20
	VEGF signaling pathway	1,90E-02	8
	p53 signaling pathway	2,00E-02	9
	Chronic myeloid leukemia	2,50E-02	9
	Glycosaminoglycan biosynthesis - keratan sulfate	2,50E-02	4
	Bacterial invasion of epithelial cells	2,70E-02	9
	Estrogen signaling pathway	2,80E-02	13
	114.Genomic_reformatting_Brain_Ischemia	2,80E-02	4
	Integrin Signaling Pathway	3,40E-02	7
	Role of Tob in T-cell activation	3,40E-02	5
	Signaling pathways regulating pluripotency of stem cells	3,50E-02	13
	Toxoplasmosis	3,60E-02	11
	Inflammatory mediator regulation of TRP channels	3,90E-02	10
	Chemical carcinogenesis - receptor activation	3,90E-02	17
	Bladder cancer	4,10E-02	6
	Complement and coagulation cascades	4,40E-02	9
	Colorectal cancer	4,70E-02	9
B: KOAGR2 vs scr; 0.67 > FC > 1.5; p < 0.05	Focal adhesion	7,30E-06*	17
	AGE-RAGE signaling pathway in diabetic complications	1,00E-05*	12

	Pathways in cancer	5,00E-04*	25
	ECM-receptor interaction	6,40E-04*	9
	TNF signaling pathway	7,30E-04*	10
	Platelet Amyloid Precursor Protein Pathway	1,70E-03	5
	Regulation of actin cytoskeleton	2,90E-03	13
	Proteoglycans in cancer	5,20E-03	12
	Amoebiasis	6,80E-03	8
	Relaxin signaling pathway	7,10E-03	9
	Leukocyte transendothelial migration	1,20E-02	8
	Human papillomavirus infection	1,30E-02	15
	Hypertrophic cardiomyopathy	1,40E-02	7
	Small cell lung cancer	1,50E-02	7
	Rheumatoid arthritis	1,60E-02	7
	Dilated cardiomyopathy	1,80E-02	7
	Chagas disease	2,40E-02	7
	Pancreatic cancer	2,50E-02	6
	Arrhythmogenic right ventricular cardiomyopathy	2,60E-02	6
	Cytokine-cytokine receptor interaction	2,80E-02	13
	TGF beta signaling pathway	3,10E-02	4
	Role of Tob in T-cell activation	3,10E-02	4
	Tight junction	3,10E-02	9
	MicroRNAs in cancer	3,80E-02	13
	Arachidonic acid metabolism	4,40E-02	5
	Axon guidance	4,60E-02	9
	PI3K-Akt signaling pathway	4,60E-02	14
	Regulators of Bone Mineralization	5,00E-02	3
C: KOAGR2 ∩ scr TGFβ; $0.67 > FC > 1.5$; $p < 0.05$	Pathways in cancer	1,70E-04*	15

	Proteoglycans in cancer	3,90E-04*	9
	Relaxin signaling pathway	8,90E-04	7
	AGE-RAGE signaling pathway in diabetic complications	1,80E-03	6
	Focal adhesion	1,80E-03	8
	Arachidonic acid metabolism	1,90E-03	5
	Human papillomavirus infection	8,00E-03	9
	MAPK signaling pathway	1,40E-02	8
	MicroRNAs in cancer	1,80E-02	8
	Platelet Amyloid Precursor Protein Pathway	2,40E-02	3
	Kaposi sarcoma-associated herpesvirus infection	2,70E-02	6
	Pancreatic cancer	2,90E-02	4
	PI3K-Akt signaling pathway	3,40E-02	8
	Chemical carcinogenesis - receptor activation	3,80E-02	6
	Hypertrophic cardiomyopathy	4,40E-02	4
	Small cell lung cancer	4,60E-02	4
	GnRH signaling pathway	4,80E-02	4
	Rheumatoid arthritis	4,80E-02	4
	Bladder cancer	5,00E-02	3
D: KOAGR2 TGF-β vs scr; 0.67 > FC > 1.5; $p < 0.05$	Focal adhesion	4,40E-10*	41
	Axon guidance	1,60E-07*	34
	ECM-receptor interaction	2,80E-07*	22
	Pathways in cancer	8,00E-07*	67
	Proteoglycans in cancer	2,70E-06*	34
	AGE-RAGE signaling pathway in diabetic complications	2,70E-06*	22
	MAPK signaling pathway	2,90E-06*	43
	PI3K-Akt signaling pathway	5,90E-06*	48
	Hippo signaling pathway	6,20E-06*	28

Human papillomavirus infection	1,10E-05*	45
Regulation of actin cytoskeleton	2,70E-05*	33
Small cell lung cancer	3,80E-05*	19
Hypertrophic cardiomyopathy	9,90E-05*	18
Arrhythmogenic right ventricular cardiomyopathy	1,80E-04*	16
Rap1 signaling pathway	1,90E-04*	30
TNF signaling pathway	5,10E-04*	19
Amoebiasis	1,30E-03*	17
114.Genomic_reformatting_Brain_Ischemia	1,60E-03*	6
Dilated cardiomyopathy	2,00E-03*	16
Tight junction	2,40E-03*	23
EGFR tyrosine kinase inhibitor resistance	2,40E-03*	14
Fluid shear stress and atherosclerosis	2,70E-03*	20
Other types of O-glycan biosynthesis	3,90E-03	10
TGF-beta signaling pathway (KEGG)	4,30E-03	15
Complement and coagulation cascades	4,60E-03	14
Transcriptional misregulation in cancer	5,50E-03	24
Calcium signaling pathway	6,60E-03	28
Hematopoietic cell lineage	6,80E-03	15
Melanoma	8,90E-03	12
Glycosaminoglycan biosynthesis - chondroitin sulfate / dermatan sulfate	9,40E-03	6
p53 signaling pathway	9,90E-03	12
Lipid and atherosclerosis	1,10E-02	25
Gastric cancer	1,20E-02	19
Relaxin signaling pathway	1,40E-02	17
Glyoxylate and dicarboxylate metabolism	1,40E-02	7
Chemical carcinogenesis - receptor activation	1,70E-02	24
Toxoplasmosis	1,90E-02	15

	Ferroptosis	1,90E-02	8
	Glycosaminoglycan biosynthesis - heparan sulfate / heparin	2,10E-02	6
	Arginine and proline metabolism	2,10E-02	9
	Platelet activation	2,10E-02	16
	Rheumatoid arthritis	2,30E-02	13
	TGF beta signaling pathway (Biocarta)	2,40E-02	6
	Cell adhesion molecules	2,40E-02	18
	Basal cell carcinoma	2,60E-02	10
	Pancreatic cancer	3,20E-02	11
	Signaling pathways regulating pluripotency of stem cells	3,30E-02	17
	Inflammatory mediator regulation of TRP channels	3,30E-02	13
	Cellular senescence	3,60E-02	18
	Hepatocellular carcinoma	3,60E-02	19
	Legionellosis	3,80E-02	9
	Platelet Amyloid Precursor Protein Pathway	4,10E-02	5
	Breast cancer	4,10E-02	17
	Glutathione metabolism	4,10E-02	9
	Ras signaling pathway	4,30E-02	24
	Yersinia infection	4,50E-02	16
	Integrin Signaling Pathway	4,50E-02	8
	Metabolic pathways	4,60E-02	121
	Protein digestion and absorption	4,60E-02	13
	Antifolate resistance	5,00E-02	6
E: KOAGR2 ∩ scr TGFβ ∩ KOAGR2 TGF β; 0.67 > FC > 1.5; p < 0.05	Pathways in cancer	1,70E-04*	15
	Proteoglycans in cancer	3,90E-04*	9
	Relaxin signaling pathway	8,90E-04	7
	AGE-RAGE signaling pathway in diabetic complications	1,80E-03	6

Focal adhesion	1,80E-03	8
Arachidonic acid metabolism	1,90E-03	5
Human papillomavirus infection	8,00E-03	9
MAPK signaling pathway	1,40E-02	8
MicroRNAs in cancer	1,80E-02	8
Platelet Amyloid Precursor Protein Pathway	2,40E-02	3
Kaposi sarcoma-associated herpesvirus infection	2,70E-02	6
Pancreatic cancer	2,90E-02	4
PI3K-Akt signaling pathway	3,40E-02	8
Chemical carcinogenesis - receptor activation	3,80E-02	6
Hypertrophic cardiomyopathy	4,40E-02	4
Small cell lung cancer	4,60E-02	4
GnRH signaling pathway	4,80E-02	4
Rheumatoid arthritis	4,80E-02	4
Bladder cancer	5,00E-02	3

* $p_{HB} < 0.05$
