# Supplementary material for: Identification of AGR2 Gene-Specific Expression Patterns Associated with Epithelial-Mesenchymal Transition
Source: Int J Mol Sci. 2022 Sep 16;23(18):10845. doi: 10.3390/ijms231810845 (PMC9504245; doi:10.3390/ijms231810845)
Supplement: Supplementary file 1 [file ijms-23-10845-s001.zip › Table S3 primer sequences.pdf]

Table S3: List of RT-qPCR primers and their sequences

Primer	Sequence
COL4A1_F	TCTTCCTTTGAAAGACTGTGCTGTCC
COL4A1_R	ACCTGGGTTTGGTTTTGGCAACA
COLRA2_F	CCCGGGCTAAAAGGAGATAG
COL4A2_R	GACCGGGTATTCCGAAAAAT
FLNA_F	CCATGACAACACCTACACAGTCA
FLNA_R	CTTGGAGATACTGCCACTGAGAA
VAV3_F	GGACCAATGGACTGCGAAGAA
VAV3_R	CTGGAGCTGTAAAGGGGGTC
VEGFA_F	CTGCTGTCTTGGGTGCATTGG
VEGFA_R	CACCGCCTCGGCTTGTCACAT
VINC_F	AAACACAGTTACACTTGTGCACCC
VINC_R	AACAGAGGGAAGTGTCCCCT
GPX2_F	GGTAGATTTCAATACGTTCCGGG
GPX2_R	TGACAGTTCTCCTGATGTCCAAA
PLA2G4A_F	ATGGCCTTGGTGAGTGATTC
PLA2G4A_R	TCAGGATCTGCTACAGCTGC
PTGS2_F	CCTGTGCCTGATGATTGC
PTGS2_R	CTGATGCGTGAAGTGCTG
GAPDH_F	TACTAGCGGTTTTACGGGCG
GAPDH_R	TCGAACAGGAGGAGCAGAGAG
